# Mechanistic Investigations of Ruthenium Catalyzed Dehydrogenative Thioester Synthesis and Thioester Hydrogenation

**DOI:** 10.1021/acscatal.1c00418

**Published:** 2021-02-15

**Authors:** Michael Rauch, Jie Luo, Liat Avram, Yehoshoa Ben-David, David Milstein

**Affiliations:** † Department of Organic Chemistry, Weizmann Institute of Science, Rehovot 76100, Israel; ‡ Department of Chemical Research Support, Weizmann Institute of Science, Rehovot 76100, Israel

**Keywords:** thioester, dehydrogenative coupling, hydrogenation, DFT, Ruthenium catalyst, thiols, alcohols

## Abstract

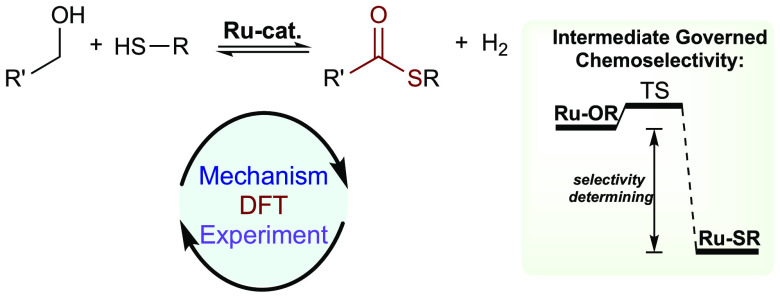

We have recently reported the previously unknown synthesis of thioesters by coupling thiols and alcohols (or aldehydes) with liberation of H_2_, as well as the reverse hydrogenation of thioesters, catalyzed by a well-defined ruthenium acridine-9H based pincer complex. These reactions are highly selective and are not deactivated by the strongly coordinating thiols. Herein, the mechanism of this reversible transformation is investigated in detail by a combined experimental and computational (DFT) approach. We elucidate the likely pathway of the reactions, and demonstrate experimentally how hydrogen gas pressure governs selectivity toward hydrogenation or dehydrogenation. With respect to the dehydrogenative process, we discuss a competing mechanism for ester formation, which despite being thermodynamically preferable, it is kinetically inhibited due to the relatively high acidity of thiol compared to alcohol and, accordingly, the substantial difference in the relative stabilities of a ruthenium thiolate intermediate as opposed to a ruthenium alkoxide intermediate. Accordingly, various additional reaction pathways were considered and are discussed herein, including the dehydrogenative coupling of alcohol to ester and the Tischenko reaction coupling aldehyde to ester. This study should inform future green, (de)hydrogenative catalysis with thiols and other transformations catalyzed by related ruthenium pincer complexes.

## Introduction

Molecules containing the thioester functional group are significant with respect to both synthetic chemistry and biochemistry. In particular, thioesters are utilized as precursors for preparing heterocycles and various materials,^1^ and the thioester functional group is prevalent biologically, most notably found in acetyl coenzyme A.^2^ Previous methods for the synthesis of thioesters rely on classical acylation of thiols with stoichiometric reagents such as carboxylic anhydrides or acyl chlorides. None of these reported processes are environmentally benign, in that they typically require activating agents or catalysts and generate large amounts of waste or byproducts.^3,4^ Relatedly, despite the fact that thioester reduction to thiols and alcohols is a well-known biosynthetic process,^5^ current chemical processes for thioester reduction similarly suffer from a lack of green methodology.^6^

Our group and others have reported several ruthenium catalysts capable of acceptorless dehydrogenative coupling (ADC) of alcohols to form esters with the only byproduct being green and utile H_2_.^7^ This reaction and the reverse reaction (hydrogenation of esters to alcohols)^8^ have garnered much interest both synthetically and mechanistically.^9^ A logical progression from these now well-studied systems would be to use the same approach to develop the dehydrogenative synthesis of thioesters from alcohols and thiols, and the reverse hydrogenation of thioesters with H_2_. However, clear challenges exist in that (i) the chemoselectivity of such processes may compete with thermodynamically preferable ester formation, whether from the dehydrocoupling of alcohols^7^ or the homocoupling of aldehydes;^10^ (ii) thiols are typically significantly more acidic than alcohols,^11^ as such, when utilized as substrates or generated as products, they are likely to poison classically utilized pincer catalysts;^12^ and (iii) generally, thiols exhibit strong coordination to metal centers,^13^ possibly inhibiting catalytic activity. Indeed, homogeneous catalysis with thiols represents an example of a more general challenge in developing catalytic systems with strongly coordinating species.

**Figure 1:**
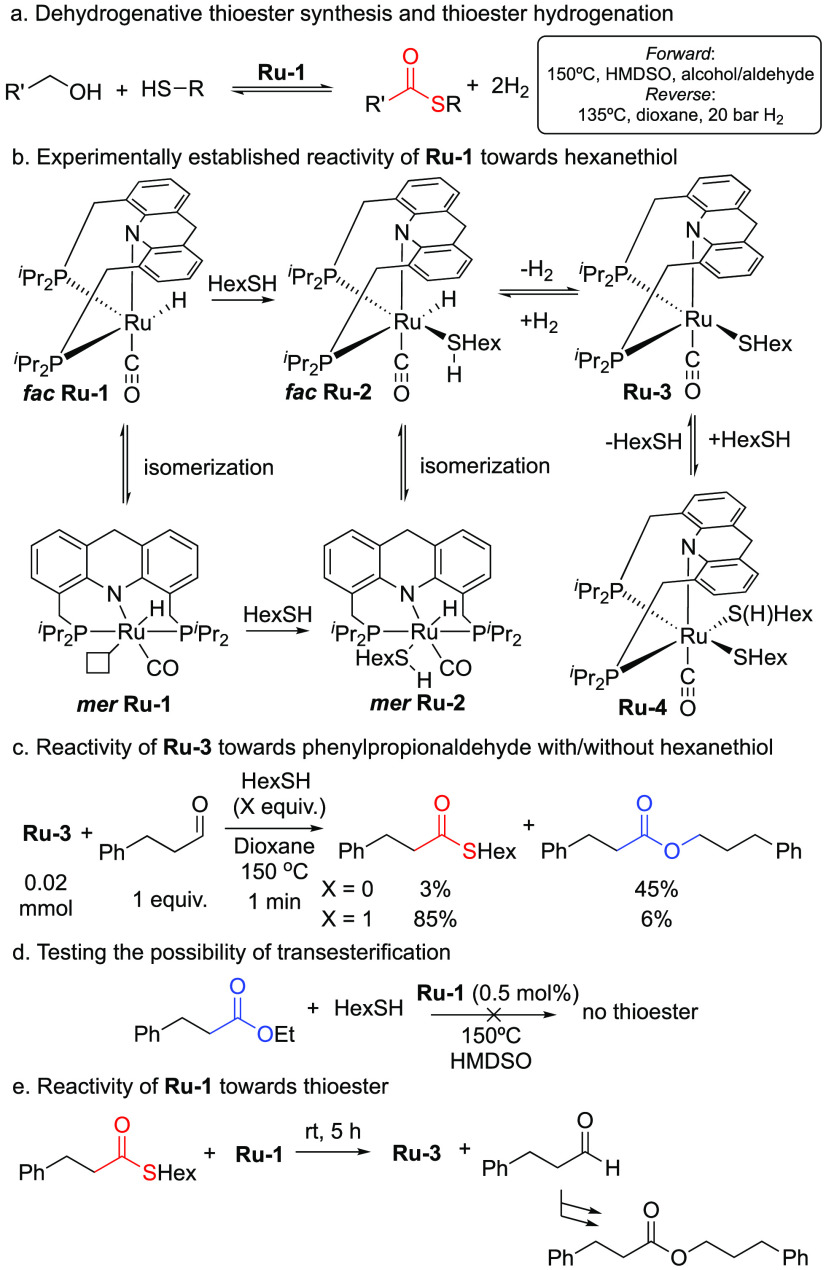
Catalytic processes and previously reported mechanistic findings.

Despite these challenges, our group recently developed the fundamentally new process for the highly selective ADC of alcohols and thiols to directly synthesize thioesters with H_2_ gas as the only byproduct.^14^ The ruthenium acridine-9H pincer complex, ^Acr^PNP^iPr^*RuH(CO) (**Ru-1**),^15^ catalyzes the reaction without any additives, and the C(=O)R source can be either alcohols or aldehydes (Figure 1a). In addition, the system can catalyze the reverse transformation, the selective hydrogenation of thioesters to thiols and alcohols under moderate hydrogen pressures (Figure 1a).^16^ Not only are these methodologies to construct and deconstruct thioesters unprecedented, but also the formation and consumption of H_2_ gas is of substantial interest with respect to atom economical synthesis, hydrogen storage, and a circular economy.^17^

Herein, we investigate the various possible productive pathways of the dehydrogenative thioester synthesis (defined here as the forward reaction) using a combined experimental and computational (DFT) approach. In doing so, the excellent chemoselectivity of the reaction toward thioester rather than ester is elucidated, which is surprising given that there is a substantial global thermodynamic preference for ester. We find that thiol and thiolate ligands serve a variety of unique selectivity-determining functions in the catalytic system. For example, the strong ruthenium affinity of sulfur as compared to oxygen drives the system toward the ruthenium hydrido thiol (**Ru-2**) and ruthenium thiolate (**Ru-3**) intermediates, preventing the persistence of the ruthenium alkoxide species (**Ru-6**) necessary for ester formation. Moreover, the thiolate ligand of **Ru-3** acts as a proton acceptor in a key outersphere alcohol dehydrogenation step (**TS**_**2,3″**_) and, in the presence of aldehyde, facilitates direct thioester formation from either a novel, concerted C–S bond forming and beta hydride elimination step (**TS**_**3,1**_) or a stepwise process (**TS**_**7,1**_). In developing a complete understanding of the forward dehydrogenative mechanism, we can also rationalize the ease at which the conditions can be manipulated to promote the reverse catalytic transformation, thioester hydrogenation to thiols and alcohols. Comprehensive analysis of the catalytic system with both experiment and computation accomplishes several related objectives: deriving the productive and competing mechanisms, rationalizing the observed chemoselectivity and informing future (de)hydrogenative catalysis employing thiols.

## Results and Discussion

### Experimental Observations

It is instructive to first briefly reiterate several critical mechanistic observations in our initial reports.^14,16^ The key findings are summarized as follows:

(i) Ruthenium acridine-9H pincer complex, **Ru-1** reacts with hexanethiol (HexSH) at room temperature to afford ^Acr^PNP^iPr^*RuH(HexSH)(CO) (**Ru-2**) and ^Acr^PNP^iPr^*RuSHex(CO) (**Ru-3**), which upon heating transforms exclusively to **Ru-3** with the elimination of H_2_ in nearly complete conversion. Under H_2_ pressure, an equilibrium with **Ru-2** is observable (Figure 1b).
(ii) **Ru-3** has been structurally characterized, and exhibits *fac* ligand coordination. While there is no explicit evidence for coordination of free alcohol to **Ru-3** in solution, **Ru-3** was demonstrated to coordinate another molecule of HexSH, as evidenced by the structural characterization of ^Acr^PNP^iPr^*RuSHex(HexSH)(CO), **Ru-4**, at low temperature, also with *fac* ligand conformation (Figure 1b). Notably, **Ru-3** exhibits similar catalytic competency as **Ru-1** for thioester synthesis and is operable in thioester hydrogenation.
(iii) Addition of a stoichiometric amount of 3-phenylpropionaldehyde to **Ru-3** in the absence of HexSH generates ester preferentially to thioester (∼15:1), whereas the same reaction in the presence of an equivalent of hexanethiol generates thioester preferentially to ester (∼14:1) (Figure 1c).
(iv) In the dehydrogenative synthesis, ester cannot be utilized as a substrate in lieu of alcohol or aldehyde. No reaction was observed from the reaction of ester and thiol under the catalytic conditions, indicating that the reaction does not proceed via transesterification (Figure 1d).
(v) Thioester reacts stoichiometrically with **Ru-1** at room temperature to afford **Ru-3** and aldehyde, which can proceed to form ester over time in the absence of hydrogen gas (Figure 1e).
(vi) In the hydrogenation of thioester, the presence of thiol does not inhibit the aldehyde hydrogenation step, whereas heating and/or higher pressures of hydrogen are needed to facilitate the conversion of thioester to aldehyde as thiol accumulates during catalysis.

We have performed several additional experiments. The specific significance of these reactions with respect to the mechanism and chemoselectivity will be realized in subsequent discussion. For comparative purposes, we have performed several catalytic reactions with alcohol or aldehyde in the absence or presence of HexSH to demonstrate the activity and selectivity of **Ru-1** for ester and thioester formation under otherwise identical conditions for a relatively short reaction time of 5 h (Table 1). Importantly, **Ru-1** is catalytically competent for dehydrogenative coupling of 3-phenyl-1-propanol to ester (58%), and ester formation directly from the corresponding aldehyde is quite facile (>99%). In the presence of HexSH, either 3-phenyl-1-propanol or 3-phenylpropionaldehyde is a suitable coupling partner to generate thioester, albeit with slightly higher yield and slightly lower selectivity for thioester in the case of aldehyde (Entries 3 and 4).

**Table 1:**
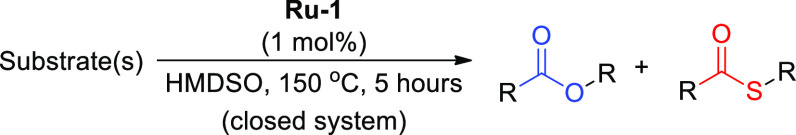

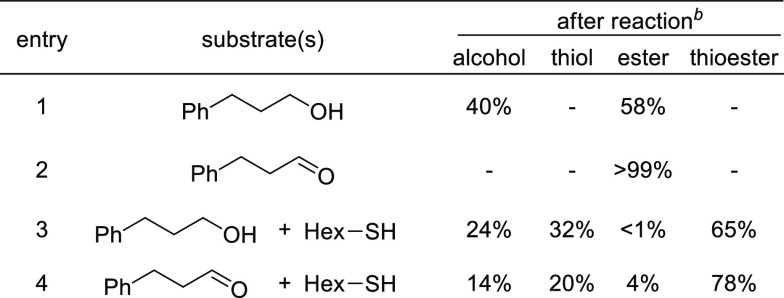
Catalytic Reactions of **Ru-1**^a^

a Conditions: substrate(s) (0.5 mmol each), catalyst (1 mol %), HMDSO (1 mL), and closed system heat for 5 h.

b Yields were determined by GC using benzyl benzoate as internal standard; yield in entries 1 and 2 based on a maximum 0.25 mmol product; yield in entries 3 and 4 based on a maximum 0.5 mmol product.

In the literature, **Ru-1** and the cyclohexyl-P-substituted analog of **Ru-1** have been utilized in ester forming reactions. Specifically, **Ru-1**, generated in situ, can catalyze dehydrogenative coupling of hexanol to hexyl hexanoate^18^ and the cyclohexyl analog of **Ru-1** is reported to homocouple benzaldehyde to benzyl benzoate.^19^ Moreover, **Ru-1** is an excellent catalyst for dehydrogenative coupling of ethylene glycol, the simplest vicinal diol, to 2-hydroxyethyl glycolate and higher order oligomers, and for the reverse hydrogenation of the mixture of oligomers back to ethylene glycol,^20,21^ leading to hydrogen carrier systems based on it.^20,21^ Thus, **Ru-1** is a known catalyst for transforming alcohols or aldehydes to esters, but the chemoselectivity is altered in the presence of thiols.

In addition, we have studied the effect of H_2_ pressure on the reaction by performing the dehydrogenative coupling of HexSH and 3-phenyl-1-propanol under varying initial H_2_ pressures (Table 2, Entries 1–5). Indeed, the yield of thioester is highly dependent on the pressure, as has been observed in related acceptorless dehydrogenation systems.^22^ Initial pressures of H_2_ gas in the system above 1.4 bar essentially prevent the dehydrogenative process, whereas when the reaction was performed in an open system, essentially quantitative yield was achieved (Table 2, Entry 6).With respect to the reverse reaction, and in accord with these findings, we reported that only 3 bar of H_2_ is required to promote thioester hydrogenation stoichiometrically, and as low as 10 bar of H_2_ is suitable for the catalytic hydrogenation.^16^ Clearly, the overall equilibrium is governed by the hydrogen pressure in the system.

**Table 2:**
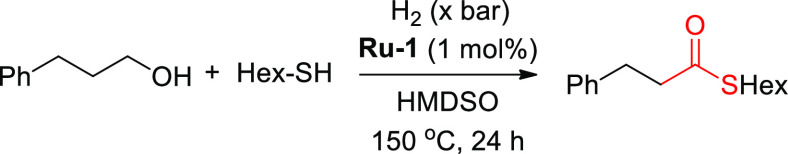
Effect of H_2_ Pressure on Thioester Yield^a^

entry	initial H_2_ pressure^b^ (bar)	thioester yields (%)^c^
1	0	93
2	0.3	82
3	0.8	60
4	1.4	31
5	1.9	<1
6	open system under Ar flow	>99

a Conditions: alcohol (0.5 mmol), Hex-SH (0.5 mmol), catalyst (1.0 mol %), HMDSO (2 mL), 24 h; and in 90 mL Fischer-porter tube with the addition of different pressures of H_2_ gas before the reaction.

b H_2_ pressure was corrected based on the collected hydrogen gas.

c Yields were determined by GC using benzyl benzoate as internal standard.

**Figure 2:**
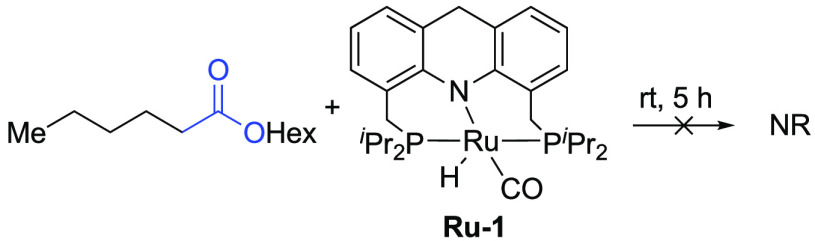
Reactivity of **Ru-1** toward ester.

Finally, with respect to the reversibility of the processes, we note that while **Ru-1** reacts with thioester at room temperature to afford **Ru-3**, aldehyde, and ester (Figure 1e), no change is observed from the corresponding reaction with ester (Figure 2).

### Computational Details

With consideration of the above experimental findings, computation (DFT) was employed to evaluate the various pathways relevant to the forward catalytic process. Ethanol and ethanethiol were studied as minimal models for the substrates in the system. Note that the various intermediates are numbered **Ru-X** (from here forward, **Ru-3**, for example, refers to the theoretical SEt derivative or the experimental SHex derivative for simplicity), and the discussed transition states are defined as **TS**_**X,Y**_ with the transition state connecting intermediates **Ru-X** and **Ru-Y**. Directionality of Δ*G* and Δ*G*_TS_ values are indicated by the ordering of X,Y and all energies are reported in kcal/mol.

DFT calculations were performed with Gaussian 16 (C.01 revision)^23^ using Truhlar’s M06-L functional,^24^ the triple-ξ def2-TZVP basis set,^25^ W06 density fitting,^26^ and Grimme’s D3(0) empirical dispersion correction.^27^ Frequency calculations at this level of theory were run at 393.15K (experimentally determined reaction temperature) to confirm stationary points and transition states and to obtain thermodynamic corrections. Single point energies of the M06-L optimized structures were computed with ORCA (4.2.1)^28^ using the range-separated meta-GGA hybrid functional ωB97M-V of the Head-Gordon group^29^ including dispersion correction,^30^ together with the triple-ξ def2-TZVPP basis set^25^ and the corresponding auxiliary basis sets, def2/J^26^ and def2-TZVPP/C^31^ for RIJCOSX density fitting. The functional and basis set selections are based on recent benchmark studies.^32^ The polarizable continuum model (IEFPCM) was used in all calculations (optimization and single point) with the SMD solvation (1,4-dioxane) model of Truhlar and co-workers.^33^ The use of 1,4-dioxane as the model solvent is justified because (i) dioxane gives similar experimental results to the experimentally optimal solvent, HMDSO, for the forward dehydrogenation reaction (ii) most mechanistic studies were performed in dioxane or HMDSO (iii) dioxane, as opposed to HMDSO, has been fully defined for the SMD model (iv) dioxane is the optimal solvent for the reverse hydrogenation. Regarding possible conformers of the acridine-9H based ruthenium complexes (in particular the orientations of the ^*i*^Pr groups) and geometries of the alkoxide, thiolate, hemiacetaloxide, and hemithioacetaloxide ligands (vide infra), several conformers for each intermediate and transition state were optimized but only the lowest energy results are presented herein.^34^ Standard state corrections^35^ were employed such that all species are treated as 1 M (using an ideal gas approximation), with the exception of H_2_ maintained as 1 atm.^36^ Other than these standard state corrections, the transformation of hydrogen from the condensed phase to the gas phase is not additionally corrected for in the free energy quantities provided. Nonetheless, the effect of H_2_ pressure on the system is studied experimentally and further discussion can be found in the Supporting Information (SI).

### Thiol and Alcohol to Thioester

**Figure 3:**
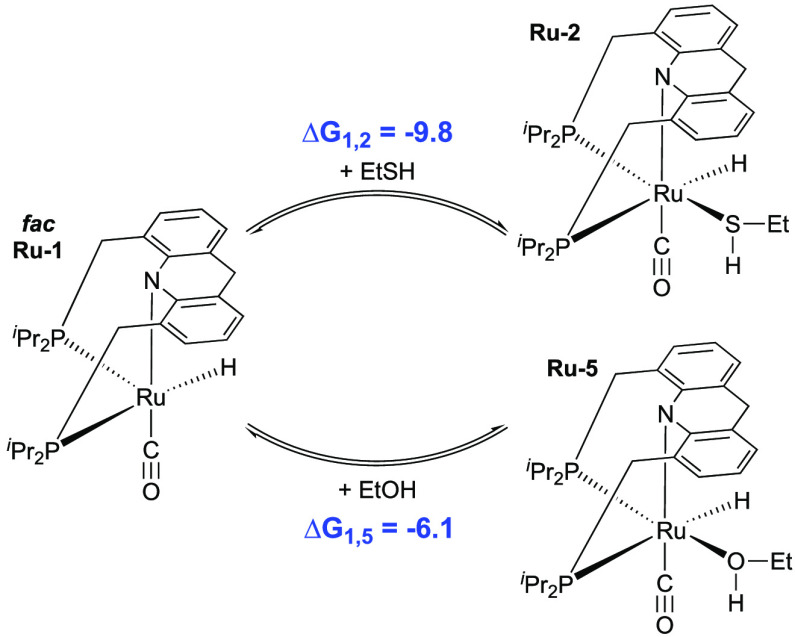
Thiol or alcohol binding to **Ru-1**.

**Ru-1** has been structurally characterized with a *mer* geometry (computationally supported as its most stable form),^37^ but previous detailed mechanistic work has shown that *fac***Ru-1** with a vacant site *cis* to the ruthenium hydride is typically the active catalytic species.^19,20,37^ Thus, the ruthenium hydride species, *fac***Ru-1**, is the presumed catalytically competent isomer, in accordance with our observation of *fac* ligand coordination for the key intermediates.^14^ Nonetheless, the zero point energy is taken as the energy of *mer***Ru-1**, which is optimized from the previously reported X-ray structure and is found to be the lowest energy conformation of the complex (10.6 kcal/mol lower than *fac***Ru-1**).^19,37^ Complex *fac***Ru-1** (referred from here forward as just **Ru-1**) can coordinate one molecule of thiol or alcohol to afford **Ru-2** or **Ru-5 (**^Acr^PNP^iPr^*RuH(EtOH)(CO)), respectively (Figure 3). In the presence of both ethanethiol and ethanol, the coordination of thiol is preferable, (ΔΔ*G*_1,2;1,5_ = −3.7 kcal/mol), supported by the experimental observation of the ruthenium hydrido thiol complex as opposed to the ruthenium hydrido alcohol complex when **Ru-1** is treated with both thiol and alcohol in the catalytic system, and in accordance with the stronger coordinative ability of thiol compared to alcohol.^13^

**Figure 4:**
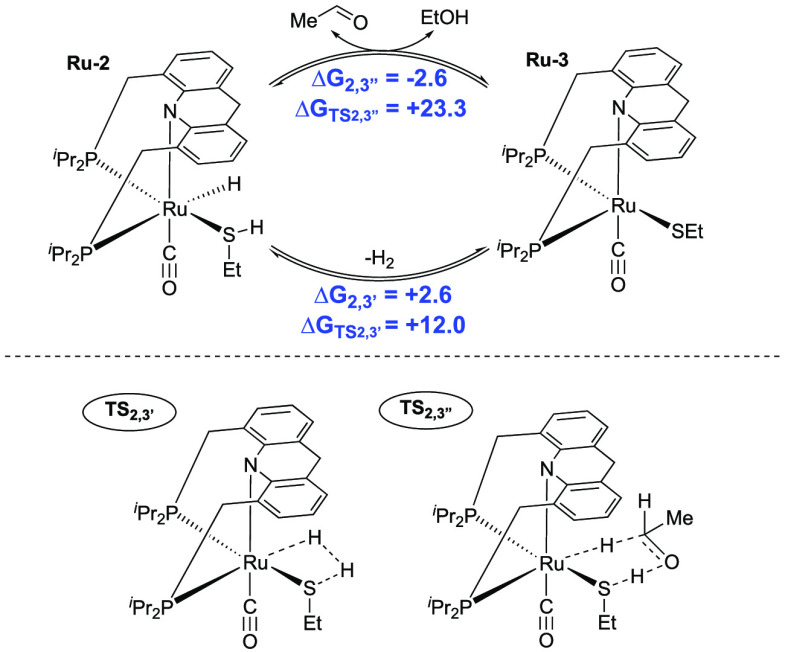
Thiol and alcohol dehydrogenation.

Complex **Ru-2** undergoes facile hydrogen elimination to afford the key ruthenium thiolate intermediate, **Ru-3** (Figure 4). This reaction has a low kinetic barrier (Δ*G*_TS2,3′_ = 12.0 kcal/mol), and the free energy of the interconversion is highly dependent on the pressure of hydrogen in the system (see SI). Specifically, the dehydrogenation of **Ru-2** to **Ru-3** is driven by H_2_ release to the headspace and then ultimately to the atmosphere (under typical conditions the vessel is opened after 5 h to drive the reaction to completion). The dependence of the yield of the overall reaction on the pressure of hydrogen is exemplified in the aforementioned H_2_ pressure experiment exhibited in Table 2, in which pressures of H_2_ above 1.4 bar significantly inhibit the reaction and in which performing the reaction in an open system results in essentially quantitative thioester formation. Note that the computed free energy for the dehydrogenation of **Ru-2** (Δ*G*_2,3′_ = +2.6 kcal/mol) assumes 1 M standard states for the non-hydrogen species and 1 atm for H_2_ gas, which should underestimate the experimental free energy benefit of hydrogen leaving the condensed phase.^38^

**Figure 5:**
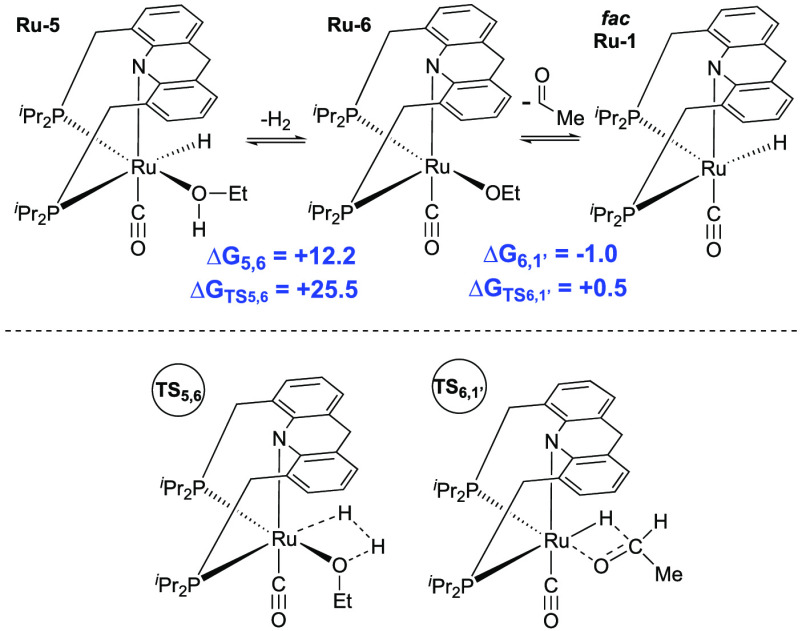
Alcohol dehydrogenation and hydride elimination.

In addition to undergoing the reverse reaction with hydrogen to afford **Ru-2**, complex **Ru-3** is alternatively capable of accepting proton and hydride from a molecule of alcohol to afford aldehyde and regenerate **Ru-2** (Figure 4). This key step is the likely source of alcohol dehydrogenation in the system, and most probably occurs through an outersphere transition state (**TS**_**2,3″**_) in which the thiolate ligand assists by accepting the proton. This concerted hydride and proton transfer resembles the proposed pathway of alcohol dehydrogenation at the related ruthenium alkoxide by Hofmann et al.^19,39^ Alternatively, there are other possible sources for the formation of aldehyde in the system. Rather than reacting with thiol to form **Ru-2**, the ruthenium hydride precursor could react with alcohol to form **Ru-5** which could also eliminate hydrogen and afford ^Acr^PNP^iPr^*RuOR(CO), **Ru-6**. The ruthenium alkoxide complex, **Ru-6**, could then undergo beta hydrogen elimination (**TS**_**6,1′**_) to release free aldehyde and reform **Ru-1** (Figure 5).^20^ However, the computation indicates that while this pathway seems plausible in the absence of thiol (see complete discussion in the ester pathway section below), in the presence of thiol, (i) **Ru-2** will form preferentially to **Ru-5** (ΔΔ*G*_1,2;1,5_ = −3.7 kcal/mol), (ii) **Ru-2** undergoes hydrogen elimination with a substantially lower kinetic barrier than does **Ru-5** (ΔΔ*G*_TS2,3′;5,6_ = −13.5 kcal/mol), and (iii) should **Ru-6** form, the desired beta hydride elimination to afford aldehyde will compete with the undesirable direct protonation of the alkoxide by thiol to afford **Ru-3** and free alcohol (via **TS**_**6,3**_, vide infra). As such, **Ru-3** (in an equilibrium with **Ru-2** dependent on H_2_ pressure) is the resting state of the system and likely operates as the alcohol dehydrogenation catalyst.

**Figure 6:**
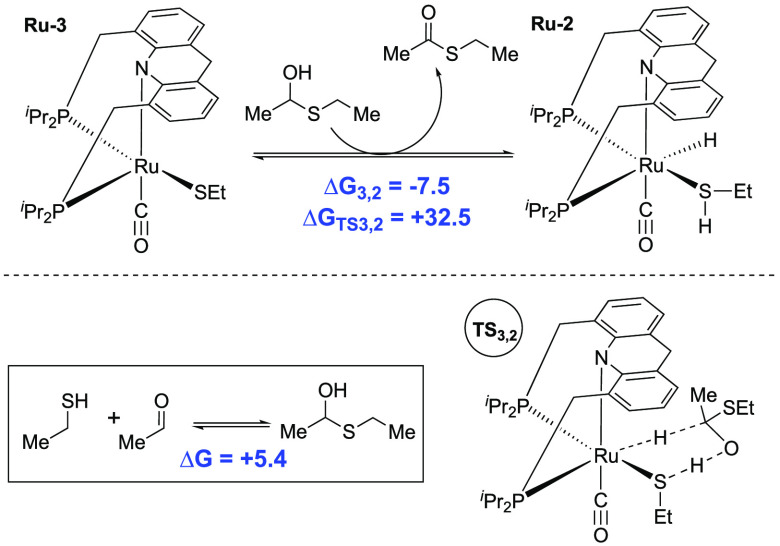
Hemithioacetal formation and dehydrogenation.

Nonetheless, aldehyde must be generated in the system, but is unlikely to build up in any substantial concentration. Indeed, alcohol dehydrogenation is calculated to be +5.1 kcal/mol endergonic (again noting standard states). Additionally, the key alcohol dehydrogenation step described above through **TS**_**2,3″**_ is readily reversible. Thus, it is unlikely that the reaction proceeds through the coupling of aldehyde and thiol to form a transient hemithioacetal (Figure 6).^40,41^ In addition to the likely low concentration of aldehyde present in the catalytic system, we note that the formation of a hemithioacetal from aldehyde and thiol is thermodynamically uphill (Δ*G* = +5.4 kcal/mol), and we also find no kinetically reasonable pathways to dehydrogenate the hemithioacetal to afford the product thioester (most notably, dehydrogenation at **Ru-3** via **TS**_**3,2**_, Figure 6). This conclusion contrasts our original proposal, but is in accord with other related dehydrogenative coupling mechanisms.^14,40^

**Figure 7:**
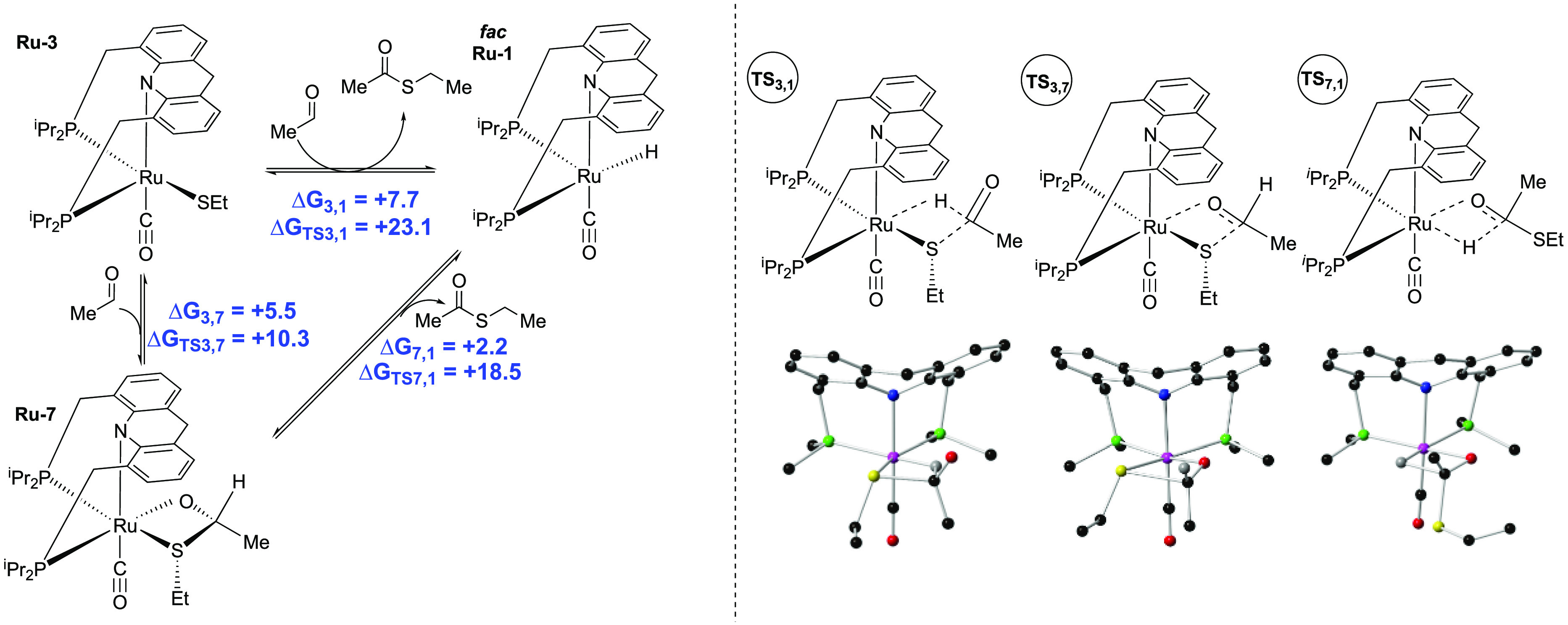
Thioester formation via C–S bond formation and beta hydride elimination.

Instead, computation suggests that two possible lower energy pathways toward product formation exist from **Ru-3** once aldehyde has been generated. Most favorably, we find that a seemingly novel transition state (**TS**_**3,1**_) exists in which aldehyde reacts with the ruthenium thiolate to directly form the new C–S bond and eliminate hydride to the ruthenium center in a single concerted process to afford the thioester (Figure 7). Analysis of the IRC indicates that the process is asynchronous, such that the C–S bond forms followed by the hydride elimination in a concerted step (see SI S8). Alternatively, the process can occur stepwise with an overall similar kinetic barrier (ΔΔ*G*_TS3,1;7,1_ = +0.9 kcal/mol).^42^ Aldehyde can formally insert into the Ru–S bond of **Ru-3** via a “click” transition state in which the oxygen coordinates to the vacant site of **Ru-3** and the new C–S bond is formed, affording **Ru-7**, a ruthenium κ^2^-hemithioacetaloxide complex.^43^**Ru-7** can then proceed to undergo beta hydride elimination (**TS**_**7,1**_) to afford product and **Ru-1**. An H-bound intermediate (**Ru-8**, vide infra) is not located as a minimum.

Regenerated **Ru-1** is again available to propagate the cycle. It is noteworthy that from the aforementioned experimental findings, **Ru-1** can react stoichiometrically with thioester to release aldehyde (and ester) and afford **Ru-3** (Figure 1e). As such, the overall dehydrogenation reaction is again driven by the strong preference for **Ru-1** to coordinate and then dehydrogenate thiol, preventing the reverse thioester insertion in the absence of H_2_ pressures. In other words, because the system rests at **Ru-2** and **Ru-3**, product formation is relatively irreversible, especially upon release of H_2_.

**Figure 8:**
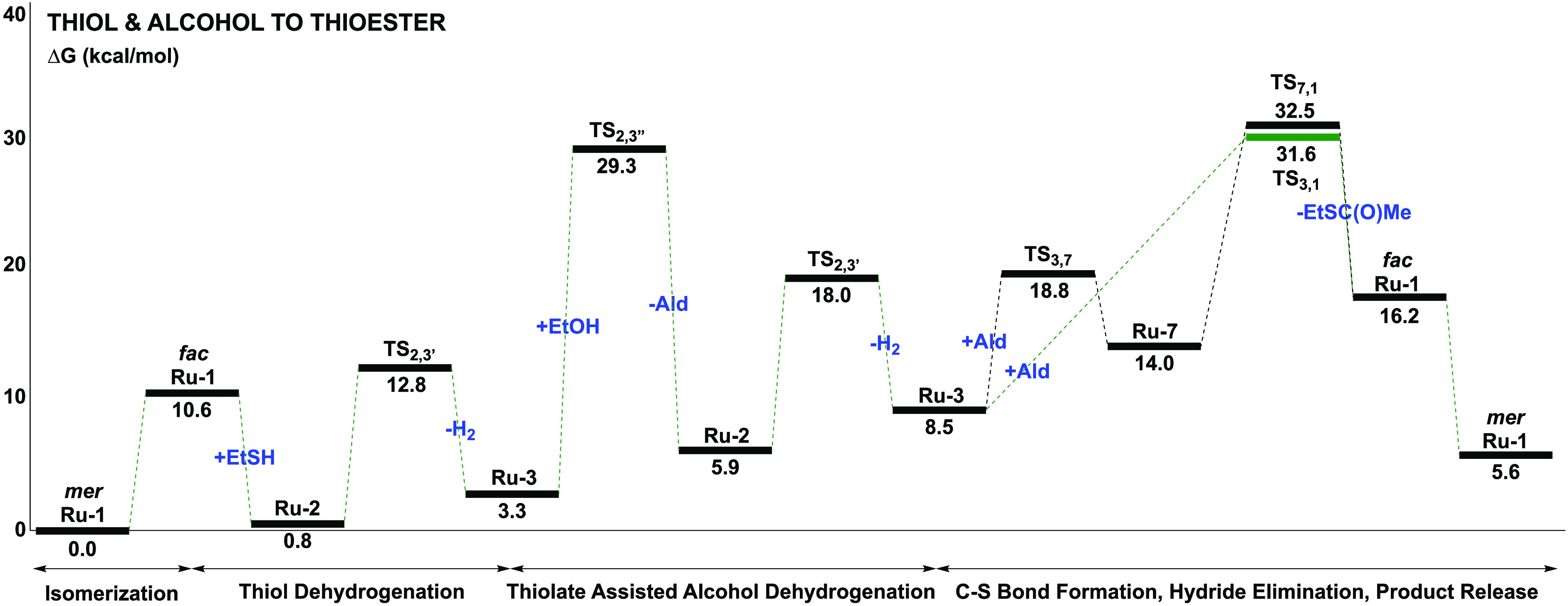
Potential energy surface for thioester formation from thiol and alcohol.

The main computed mechanism for the catalytic transformation is depicted in Figure 8, including the relative energies with respect to *mer***Ru-1**, ethanol and ethanethiol and the key transition states. It should be noted that despite a “linear” depiction of the reaction passing through **Ru-2** and **Ru-3** twice, the catalytic process can be understood as two cycles in which separate molecules of the ruthenium thiolate can operate independently, i.e., alcohol dehydrogenation can be happening simultaneously with product formation at separate catalyst molecules. Such independent but interlinked processes are common in dehydrogenative coupling reactions and have been discussed previously with respect to amide synthesis.^40^

### Thiol and Aldehyde to Thioester

**Figure 9:**
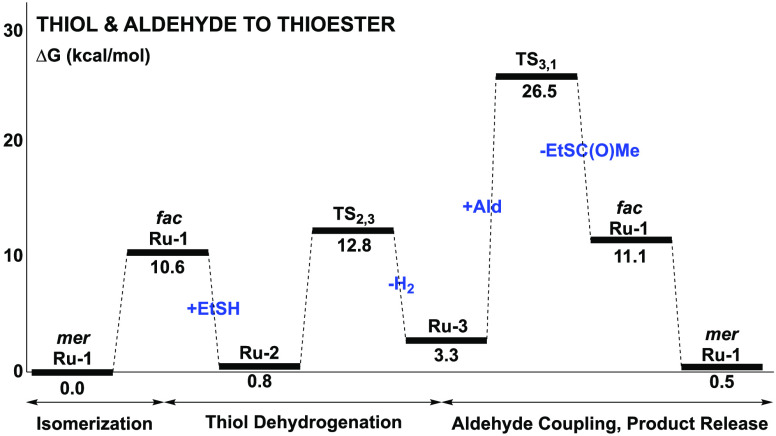
Potential energy surface for thioester formation from thiol and aldehyde.

Further supporting the proposed mechanism, we observe experimentally that aldehyde is a suitable substitute for alcohol in the system, providing slightly lower selectivity but a faster reaction (Table 1, 78% thioester, 4% ester). The computed lowest energy pathway from aldehyde and thiol to thioester is shown in Figure 9, and directly resembles the pathway from alcohol and thiol with the key difference being the thermodynamic favorability of bypassing the initial alcohol dehydrogenation. Nonetheless, the experimental chemoselectivity is slightly worse, presumably due to the ease at which aldehyde can undergo homocoupling to afford ester (vide infra). Interestingly, eventually all of the aldehyde substrate that is not directly taken to product is converted to alcohol from transfer hydrogenation (either proton and hydride transfer via **TS**_**2,3″**_ or proton transfer via **TS**_**6,3**_ vide infra, see Table 1 Entry 4), essentially resuming the main alcohol and thiol to thioester cycle (Figure 8). This explains the experimental observation in our initial report that aldehyde and thiol can give some thioester even at room temperature^14^ (overall kinetic barrier of 26.5 kcal/mol), but eventually the conversion slows substantially, indicating that any additional free aldehyde has been hydrogenated to alcohol, resulting in a larger overall kinetic barrier (31.6 kcal/mol) for the catalytic process with the added energetic cost of alcohol dehydrogenation (+5.1 kcal/mol, dehydrogenation of ethanol to acetaldehyde).

### Alcohol to Ester

As demonstrated in Table 1, as well as in previous reports,^18,20,21^ the dearomatized ruthenium acridine-9H catalyst is also competent for the dehydrogenative coupling of alcohols to esters. As compared to the dehydrogenative coupling of alcohol and thiol, the dehydrocoupling of alcohol to ester likely occurs via somewhat different corresponding pathways. First, alcohol can coordinate to the cis vacant site of **Ru-1**, generating **Ru-5**, which can release hydrogen and afford the ruthenium alkoxide complex, **Ru-6** (Figure 3 and Figure 5). It is worth noting that the kinetic barrier for this transformation via **TS**_**5,6**_ (overall barrier 30.0 kcal/mol) is likely an upper limit of the energy requirement, recognizing that additional molecules of alcohol can likely facilitate the process with hydrogen bonding interactions.^9a^ Nonetheless, the ruthenium alkoxide complex, **Ru-6**, undergoes essentially barrierless beta hydride elimination (0.5 kcal/mol kinetic barrier from **Ru-6** to **TS**_**6,1′**_) to regenerate the ruthenium hydride catalyst and eliminate a molecule of aldehyde (Figure 5). The dehydrogenation process can occur a second time, such that a free molecule of aldehyde is now available to react with **Ru-6**. It is worth noting that the second dehydrogenation could also occur outersphere at **Ru-6** through a proton and hydride concerted transfer (similar to **TS**_**3,2″**_), which Hoffman et al. calculate to occur via a similarly low kinetic barrier (+2.5 kcal/mol) in their system.^19^

**Figure 10:**
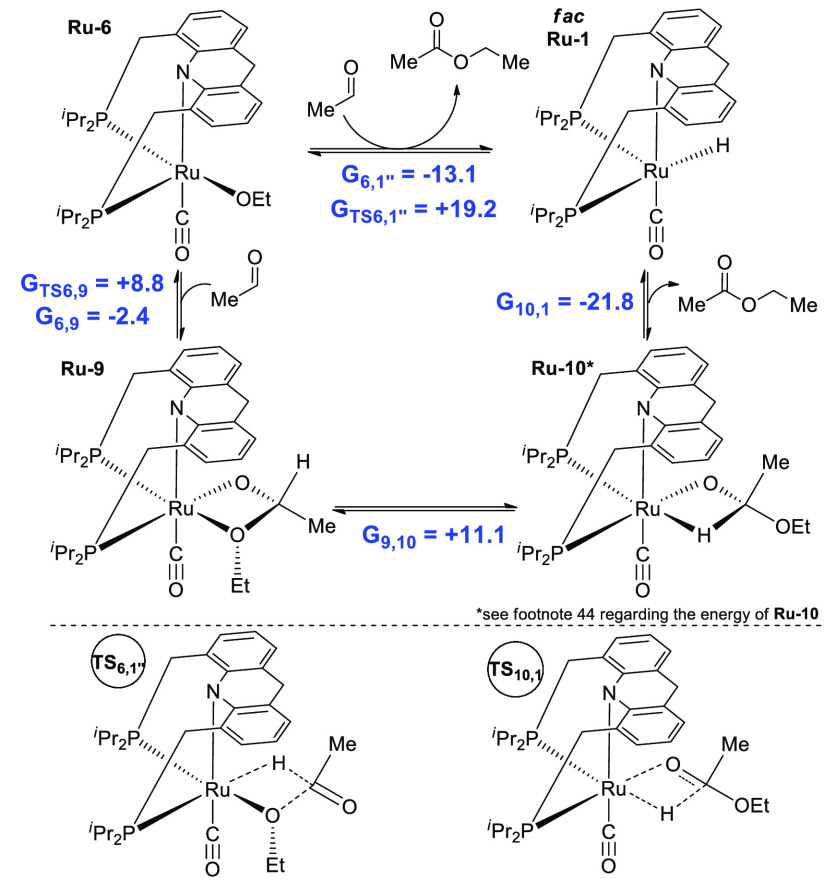
Ester formation via C–O bond formation and beta hydride elimination.

Similar to the scenario regarding the ruthenium thiolate complex, the aldehyde can couple with the ruthenium alkoxide complex either to directly afford the ester and regenerate **Ru-1** (via **TS**_**6,1″**_) or first can insert to generate a ruthenium hemiacetaloxide complex, **Ru-9** (Figure 10). In this case, the kinetic barrier to form the C–O bond and beta hydride eliminate in a concerted process through **TS**_**6,1″**_ is kinetically disfavored (overall barrier 41.0 kcal/mol), and the reaction likely proceeds first through the ruthenium hemiacetaloxide intermediate **Ru-9**. From **Ru-9**, product formation occurs via a rotation to give the H-bound ruthenium hemiacetaloxide isomer, **Ru-10**, followed by again essentially barrierless beta hydride elimination to afford the ester and regenerate **Ru-1**.^44^

Two noteworthy differences in the ester forming mechanism as compared to that of thioester formation are (i) whereas the ruthenium alkoxide complex undergoes beta hydride elimination quite readily to generate aldehyde, the corresponding ruthenium thiolate species does not do so to afford thioaldehyde, to any observable extent (vide infra) and (ii) the ruthenium thiolate complex can productively couple aldehyde to generate the thioester directly (**TS**_**3,1**_) or proceed through the hemithioacetaloxide complex (**TS**_**7,1**_), whereas the ruthenium alkoxide has a strong energetic preference to only proceed through the hemiacetaloxide pathway (**TS**_**10,1**_ not **TS**_**6,1″**_). Such differences underscore the strength of the coordinative ability of thiol and thiolate ligands to ruthenium as compared to alcohol and alkoxide ligands.

**Figure 11:**
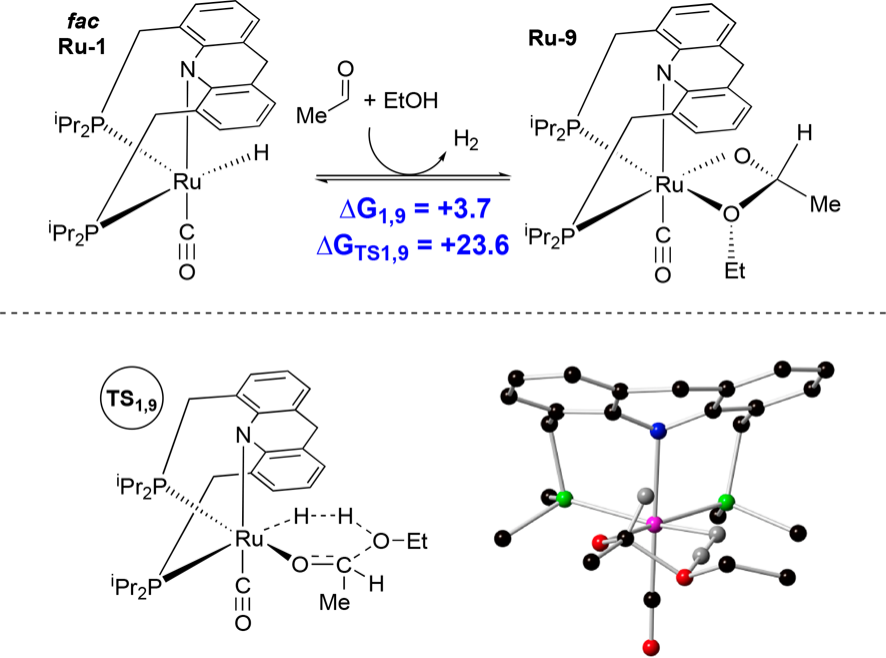
Alternative pathway for second dehydrogenation in an ester-forming mechanism.

It should be noted that additional pathways are worth considering with respect to the alcohol dehydrogenation steps. In the case of diols, we previously proposed that the second alcohol dehydrogenation occurrs via a Zimmerman–Traxler-like six-membered transition state to directly afford the ruthenium hemiacetaloxide.^20^ Here, we find that pathway (**TS**_**1,9**_) to be a bit higher in energy than a second innersphere dehydrogenation through **TS**_**5,6**_ (Figure 11). The energy difference is not too large (+4.2 kcal/mol), and the discrepancy may be due to the nuanced hydrogen bonding capabilities and π interactions observed in the diol system as opposed to the simple aliphatic alcohol system studied here.^20^

**Figure 12:**
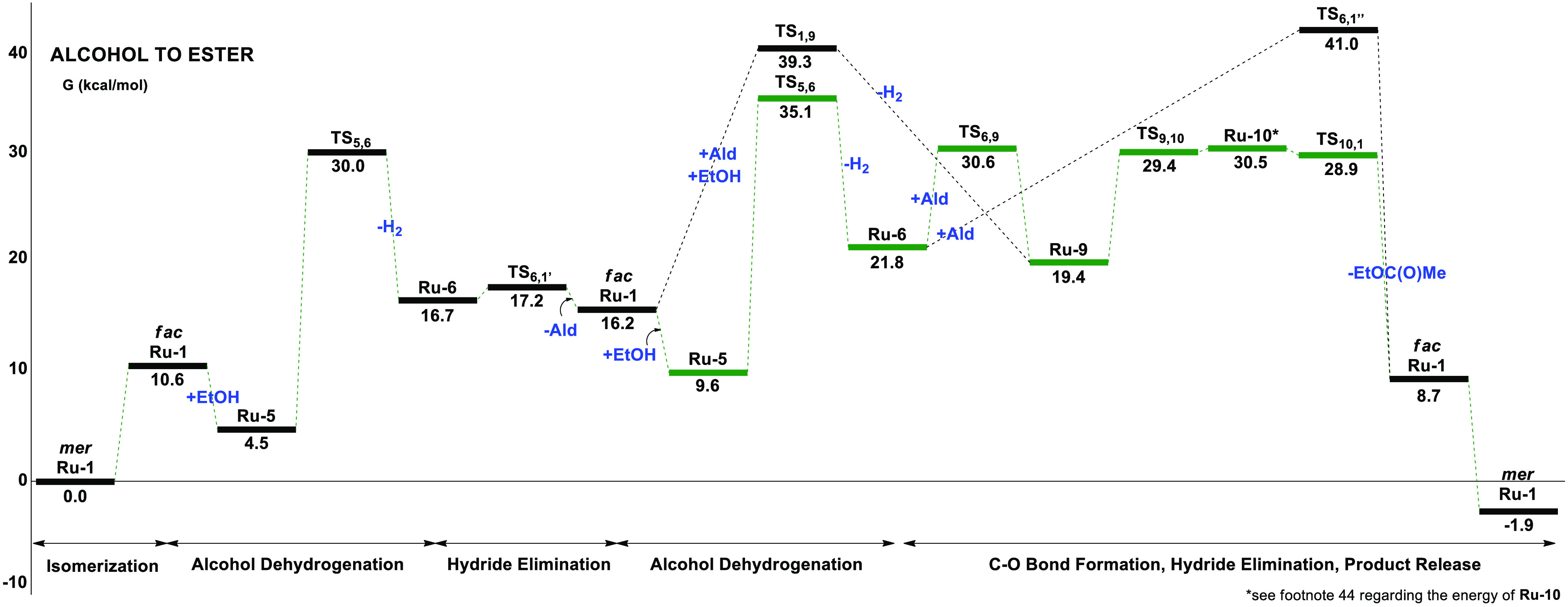
Potential energy surface for ester formation from alcohol.

The overall pathway for ester formation from alcohol is depicted in Figure 12. Whereas the reaction is similar to thioester formation in that it is driven by hydrogen elimination from the system, it is also worth noting that the global thermodynamic favorability of ester formation contributes to driving the reaction forward. Experimentally supporting this notion, unlike thioester, we demonstrate that ester does not react with **Ru-1** stoichiometrically at room temperature (Figure 2).

### Aldehyde to Ester

A final competing mechanism exists, particularly when aldehyde is employed as a substrate, which is the homocoupling of aldehyde to ester. It is noteworthy that while acceptorless alcohol dehydrogenative coupling to ester has a similar overall kinetic barrier to thioester formation from alcohol and thiol, the homocoupling of aldehyde, or the so-called Tischenko reaction, appears experimentally to be the most facile transformation herein (Table 1). The pathway for the homocoupling of aldehyde to ester is depicted in the SI (page S10), and without the relatively higher energy dehydrogenation steps, the overall kinetic barrier for ester formation is just 20.3 kcal/mol and the global thermodynamics are extremely favorable (−12.1 kcal/mol). Indeed, this supports our experimental finding in the original report,^14^ recapped in Figure 1c, in which we observe predominantly ester formation from the stoichiometric reaction of **Ru-3** and 3-phenylpropionaldehyde in the absence of additional hexanethiol. Specifically, we propose that trace thioester is formed initially, regenerating the ruthenium hydride catalyst, which is readily able to catalyze the facile homocoupling of aldehyde. However, when stoichiometric hexanethiol is also added to the reaction mixture, the homocoupling of aldehyde is prevented and thioester is the dominant product (see subsequent discussion on chemoselectivity).

### Catalytic Hydrogenation: Thioester to Thiol and Alcohol

While the discussion thus far has been tailored for understanding the forward reaction of thioester synthesis, the mechanism of the reverse hydrogenation process can be inferred from the above experimental and computational framework (Figure 8, read right to left). Specifically, hydrogenation is initiated at the ruthenium hydride catalyst, **Ru-1**, which in the presence of thioester can release aldehyde and afford **Ru-3** via either (i) concerted Ru–S bond formation, hydride transfer and aldehyde elimination through **TS**_**3,1**_ or (ii) insertion of the thioester carbonyl moiety into the ruthenium hydride (**TS**_**7,1**_) to afford the ruthenium hemithioacetaloxide species, **Ru-7**, which can eliminate aldehyde (**TS**_**3,7**_). Under hydrogen pressure, the equilibrium between **Ru-3** and **Ru-2** should strongly favor **Ru-2**, which can release free thiol, availing the ruthenium center to repeat the hydrogenative process. Indeed, experimentally, **Ru-2** is observed as the sole resting state during the hydrogenation cycle. Simultaneously, aldehyde hydrogenation to alcohol can occur outersphere at a molecule of **Ru-2** via the concerted proton and hydride transfer of **TS**_**2,3″**_, affording **Ru-3**, which again can rapidly uptake H_2_. It should be noted that the overall thioester hydrogenation process is computed to be exoergic (Δ*G* = −5.6 kcal/mol), bearing in mind the same standard state considerations alluded to throughout. Finally, we wish to emphasize that this computationally supported mechanism is in good agreement with our experimental observations regarding thiol inhibition (observation vi above), specifically that accumulation of thiol inhibits the initial conversion of thioester to aldehyde but not the hydrogenation of aldehyde to alcohol.^16^

### Chemoselectivity

**Figure 13:**
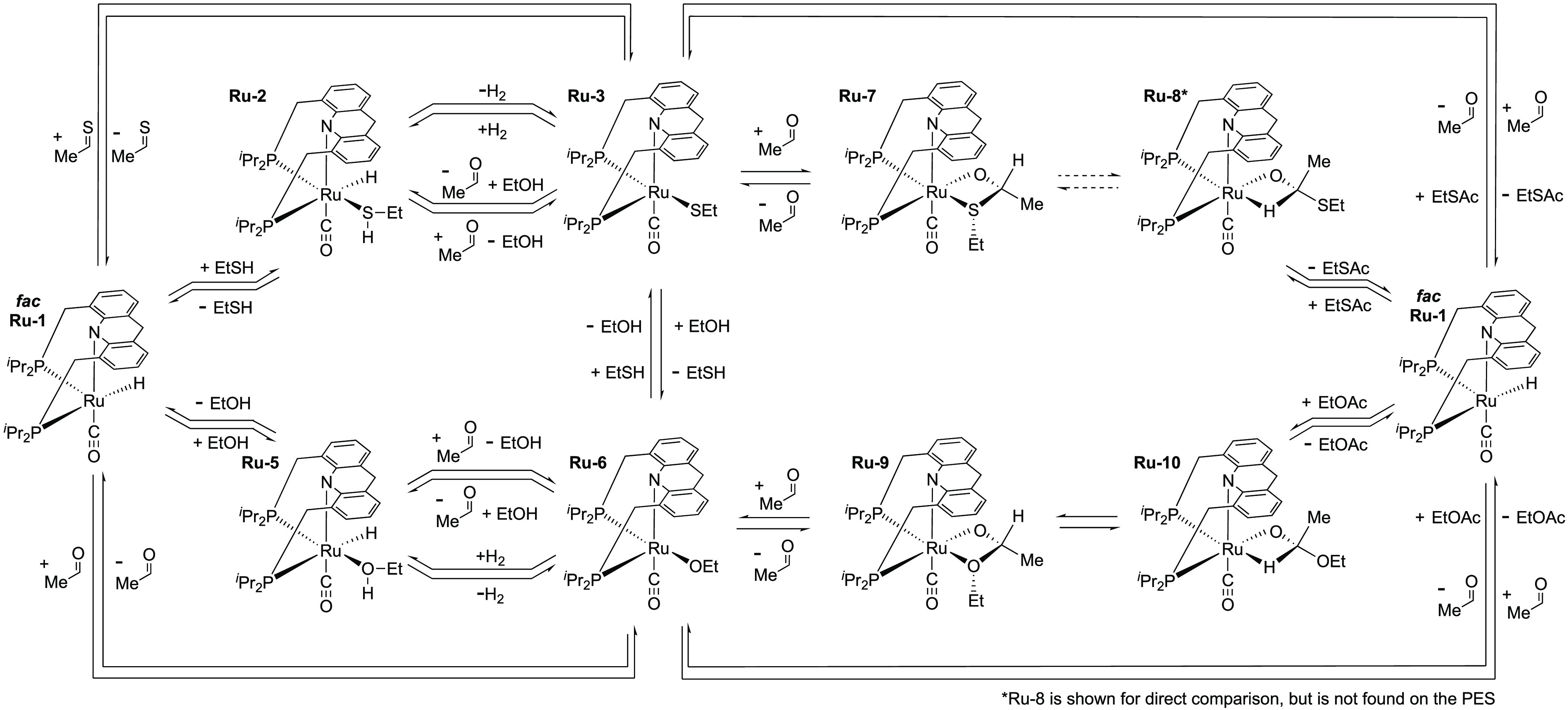
Global overview of competitive thioester and ester forming pathways.

**Figure 14:**
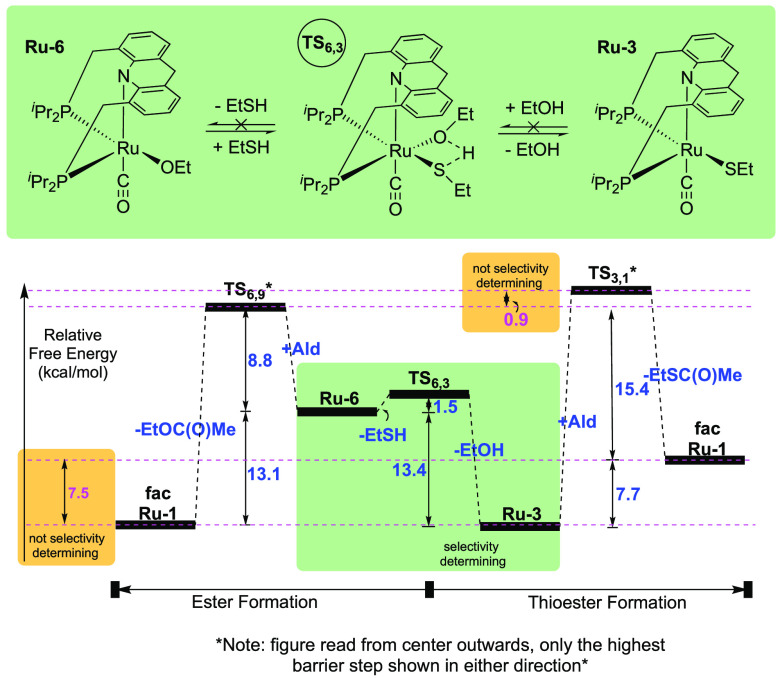
Selectivity governing parameters.

An overview of a global schematic is provided in Figure 13 demonstrating the possible intersections of the various pathways. With a broad experimental and computational understanding of the several possible pathways, we can rationalize the chemoselectivity outlined in Table 1 with respect to the forward dehydrogenative process. Interestingly, from alcohol and thiol, there is a global thermodynamic preference for ester formation rather than thioester formation (ΔΔ*G*_Thioester;Ester_ + 7.5 kcal/mol). As such, if **Ru-3** and **Ru-6** can interchange in the presence of thiol and alcohol, one might expect the reaction selectivity to be governed by Curtin-Hammett principles. In this instance, we know that both ester formation and thioester formation are kinetically feasible with similar overall barriers (demonstrated experimentally in Table 1 and theoretically in Figures 8 and 12). Thus, at the relatively high temperature employed, one might expect ester formation to be preferential as governed by thermodynamic control. However, the reaction is of course quite selective for thioester formation. We attribute the excellent selectivity to the difference in stability of the ruthenium thiolate complex as compared to the ruthenium alkoxide complex in the presence of both thiol and alcohol (Figure 14). Specifically, the magnitude of Δ*G* for the formation of the ruthenium thiolate complex from the ruthenium alkoxide intermediate (**Ru-6** to **Ru-3**) is quite large, −13.4 kcal/mol, such that this reaction must occur *essentially irreversibly* rather than in an equilibrium of reasonable unity to afford a Curtin-Hammett situation. The substantial difference in thiol acidity as compared to alcohol is directly associated with this outcome.^11^ While ester formation is favored overall thermodynamically, in the absence of alkoxide and thiolate interchange, thermodynamic control of the *intermediates* leads to the overall less thermodynamically favored *product*.^45^

We conclude that in the presence of thiol, **Ru-1** will preferentially form **Ru-2**, and **Ru-6** will irreversibly form **Ru-3**. Both of these steps are significant in preventing the forward reaction from proceeding toward ester formation despite thermodynamic preference. This underscores another unique role of thiol in the catalytic system. The strong ruthenium affinity of sulfur, as compared to oxygen (rationalized as either the high acidity of thiols or the strong coordinative ability of thiol and thiolate ligands), affords the ruthenium thiolate (**Ru-3**) and ruthenium hydrido thiol complexes (**Ru-2**) significant stability as compared to the oxygen containing counterparts, ensuring that the chemoselectivity favors thioester formation.

Clearly the general characteristics of thiols are consequential for several aspects of the catalysis. Of course, thiol serves as substrate in the system. In addition, the stronger coordinative ability and acidity of thiol ensures the intermediacy of the important ruthenium thiolate intermediate, governing the selectivity. Once the ruthenium thiolate forms, the thiolate ligand itself is important in that it can accept the proton in the outersphere alcohol dehydrogenation step. Finally, the presence of thiol in the system prevents the reverse insertion of thioester into the ruthenium hydride, by most preferably binding and reacting. In addition to the unique roles of thiol in this system, it is apparent that the ruthenium acridine-9H catalyst itself is specifically suited for this transformation. Not only is the ligand framework robust under the acidic conditions, but also the ability to access a cis vacant site for substrate coordination and hydride elimination is key to the mechanism.

### Thioaldehyde Intermediacy

**Figure 15:**
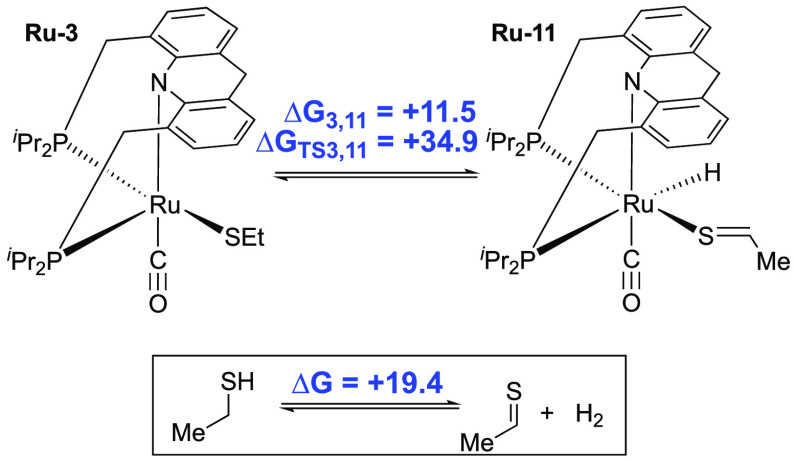
Thiol dehydrogenation.

With respect to the fates of the various intermediates, it is noteworthy that while **Ru-6** undergoes facile beta hydride elimination to afford **Ru-1** and aldehyde (Figure 5), **Ru-3**, it seems, does not undergo beta hydride elimination to afford thioaldehyde (Figure 15, top).^46^ While such a process is computed to possibly be kinetically accessible at high temperature (Δ*G*_TS3,11_ = 34.9 kcal/mol), the thermodynamics of thiol dehydrogenation (Figure 15, bottom) are far more challenging than that that of alcohol dehydrogenation. This also supports why we see no formation of thionoester or dithioester in our system. Nonetheless, it seems the beta hydride elimination of **Ru-3** to afford the ruthenium hydride complex with bound thioaldehyde, **Ru-11**, could possibly be achievable (Δ*G* = +11.5 kcal/mol). Herein we see no evidence for such a process occurring, which in this system is likely beneficial for the chemoselectivity of the overall transformations. Nonetheless, there is great interest in developing systems that could dehydrogenate thiol to thioaldehyde, possibly opening new avenues of dehydrogenative coupling reactivity. The computation indicates that to do so, one would need to either (i) utilize thiols with especially stable corresponding thioaldehydes and/or (ii) construct a system with an extremely favorable thermodynamic trap for the short-lived (if at all) presence of bound thioaldehyde.

### Vacant Site Coordination Competition

**Figure 16:**
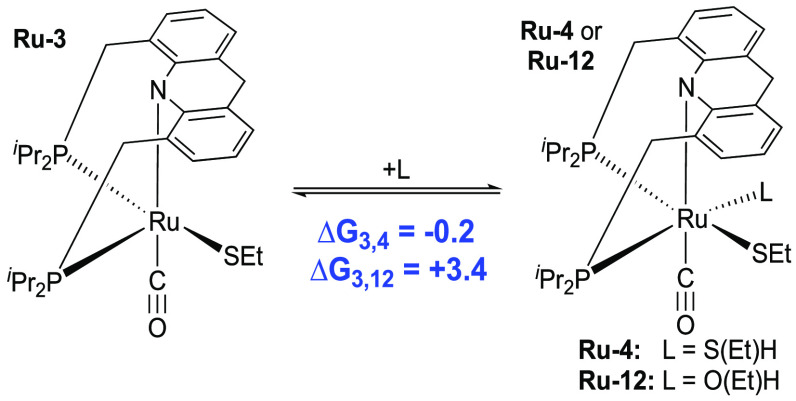
Coordination of thiol or alcohol to the vacant site of **Ru-3**.

While thioester formation in the forward reaction depends on aldehyde addition to **Ru-3** (via **TS**_**3,1**_ or **TS**_**7,1**_), it is noteworthy that the other organic species present in high concentration in the system can compete to bind the vacant site of the ruthenium thiolate intermediate.^47^ For example, we have reported the structure of **Ru-4** (Figure 1b), in which a molecule of thiol binds the vacant site of **Ru-3**. Indeed, thiol or alcohol can both, in principle, inhibit aldehyde coordination (Figure 16). We have computed the Δ*G* of each of these binding processes, and while it is clear that binding an additional molecule of thiol can readily occur, in agreement with experimental observation, the free energy is such that these binding processes are assuredly reversible, allowing for aldehyde to access the metal center.

## Conclusions

In conclusion, herein we describe a detailed overview of the ruthenium acridine-9H based catalyzed dehydrogenative coupling of alcohol (or aldehyde) and thiol to thioester, as well as the reverse transformation for thioester hydrogenation. We demonstrate the key steps of the main thioester forming pathway: (i) binding of thiol to *fac***Ru-1**, (ii) dehydrogenation to afford the ruthenium thiolate intermediate, (iii) outersphere alcohol dehydrogenation at the ruthenium thiolate intermediate to generate incipient aldehyde, and (iv) C–S bond formation and beta hydride elimination to afford thioester and regenerate the ruthenium hydride catalyst. Competing mechanisms for ester formation were analyzed in detail to rationalize the exquisite chemoselectivity for thioester formation observed experimentally. Furthermore, the major role of H_2_ pressure in thioester synthesis was studied experimentally, with the salient observation of high catalytic efficiency in an open system versus nearly a complete lack of competency with greater than 1.4 bar of initial H_2_ pressure. Accordingly, we can also rationalize the ease via which the system can facilitate the reverse transformation, thioester hydrogenation, under modest hydrogen pressures.

Moreover, we have elucidated the several key roles of thiols and thiolate ligands in the catalytic system. For example, in the dehydrogenative process, preferably to free alcohol, free thiol binds the vacant site of the ruthenium hydride complex driving the system toward the ruthenium thiolate intermediate and inhibiting counterproductive, reversible thioester insertion. Then, the ruthenium thiolate complex (**Ru-3**) formed after H_2_ liberation facilitates both outersphere alcohol dehydrogenation (**TS**_**2,3″**_) and innersphere thioester formation (**TS**_**3,1**_ or **TS**_**7,1**_). More generally, the chemoselectivity toward thioester rather than ester is governed by the relative stability of the Ru-SR containing species as opposed to the Ru-OR containing species. This difference prevents the persistence of the ruthenium alkoxide complex necessary for ester to form in any appreciable quantities, *despite* similar energy barriers and a global thermodynamic preference for ester formation rather than thioester. Informed by this mechanistic work, ongoing research in the group regarding (de)hydrogenative reactions with thiols is underway.

## Supporting Information Available

Experimental and computational details (PDF)
