# Supplementary material for: Mechanistic Investigations of Ruthenium Catalyzed Dehydrogenative Thioester Synthesis and Thioester Hydrogenation
Source: ACS Catal. 2021 Feb 15;11(5):2795–807. doi: 10.1021/acscatal.1c00418 (PMC7976608; doi:10.1021/acscatal.1c00418)
Supplement: Supplementary file 1 — cs1c00418_si_001.pdf [file cs1c00418_si_001.pdf]

Supporting Information

Mechanistic Investigations of Ruthenium Catalyzed Dehydrogenative Thioester Synthesis and Thioester Hydrogenation

Michael Rauch[†,§], Jie Luo[†,§], Liat Avram[‡], Yehoshoa Ben-David[†], David Milstein[†,*]

† Department of Organic Chemistry, Weizmann Institute of Science, Rehovot, 76100, Israel

‡ Department of Chemical Research Support, Weizmann Institute of Science, Rehovot, 76100, Israel

§ These authors contributed equally.

* Corresponding author: david.milstein@weizmann.ac.il

Contents:

General Experimental Information……………………….....………………………………..…..S2

Experimental Procedures and Data...……………………..S3

General Computational Information…………………………......……………......……S5

Discussion of H_2 Gas and Standard States………………………............……………......……S7

Concerted, Asynchronous Nature of $TS_{3,1}$.......................………...........…………….......……S8

$TS_{3,1}$ and $TS_{7,1}$ with the Experimentally Employed Substrates ....…………….......……S9

Pathway from Aldehyde to Ester……………………….....……………...………………......S10

Cartesian Coordinates……………….............………......……………………………......…….S11

Energy Data…………………………..............……….......…………………………......…….S70

GENERAL EXPERIMENTAL INFORMATION

All experiments were carried out in a glovebox under inert atmosphere of purified nitrogen using standard Schlenk techniques. All solvents were reagent grade or better. All non-deuterated solvents were refluxed over sodium/benzophenoneketyl and distilled under argon atmosphere. Deuterated solvents were used as received. All solvents were degassed with argon and kept in the glove box over 4Å molecular sieves. All substrates were directly purchased and kept in the glove box (liquids were degassed). 3-phenylpropionaldehyde was distilled prior to use. NMR spectra were recorded on Bruker AVANCE III (300 or 400 MHz) or AVANCE III HD (500 MHz) spectrometers and are reported in ppm (δ). Chemical shifts were referenced to the residual solvent peaks (^{1}H NMR, ^{13}C NMR) or an external standard of phosphoric acid (85% solution in D_2O) at 0.0 ppm (^{31}P NMR). GC analysis was performed on HP 6890 series GC system with Hp-5 column and SUPELCO 1-2382 column, flame ionization detector, and N_2 as carrier gas. GC-MS was carried out on HP 6890 / HP 5973 (MS detector) instruments equipped with a 30 m column (Restek 5MS, 0.32 mm internal diameter) with a 5% phenylmethylsilicone coating (0.25 mm) and helium as carrier gas.

Complex **Ru-1**[1] was prepared according to literature procedure.

Caution: *(i) Gas-evolving syntheses at high temperature should be performed in vessels of appropriate size and make (ii) Hydrogen is a flammable gas. Reactions associated with H_2 gas should be handled carefully inside proper fume hoods without any flame, spark or static electricity sources nearby.*

EXPERIMENTAL PROCEDURES AND DATA

General procedure for side-by-side dehydrogenative synthesis of thioesters and esters:
In a N_2 glove box, **Ru-1** (0.003 g, 0.005 mmol), the corresponding substrates (0.5 mmol) and HMDSO (1 mL) were added to a 50 mL Schlenk tube. The tube was sealed properly, removed from the glovebox, and heated at 150 °C with stirring. After 5 h, the Schlenk tube was cooled down to room temperature and the formed gas was collected. Then, 0.5 mmol internal standard (benzyl benzoate) was added. The solution was filtered through Celite, rinsed with ethyl acetate (2 mL) and analyzed by GC to determine the yields of generated products.

General procedure for dehydrogenative synthesis of thioesters under H_2 pressure:
In a N_2 glove box, **Ru-1** (0.003 g, 0.005 mmol), Hex-SH (59 mg, 0.5 mmol), 3-phenyl-1-propanol (68 mg, 0.5 mmol) and HMDSO (2 mL) were added to a 90 mL Fischer-Porter tube. The Fischer-Porter tube was taken out of the glovebox, and pressurized with H_2. The reaction was heated at 150 °C with stirring. After 24 h, the tube was cooled down to room temperature and the H_2 was released. Then, 0.5 mmol internal standard (benzyl benzoate) was added. The solution was filtered through Celite, rinsed with ethyl acetate (2 mL) and analyzed by GC to determine the yield of thioester.
Note: The pressure gauge of the Fischer-Porter was corrected based on the experimentally collected gas volume (3 times for each pressure).

General procedure for dehydrogenative synthesis of thioester in an open system:
In a N_2 glove box, **Ru-1** (0.003 g, 0.005 mmol), Hex-SH (59 mg, 0.5 mmol), 3-phenyl-1-propanol (68 mg, 0.5 mmol) and HMDSO (1 mL) were added to a 15 mL Schlenk flask. The flask was taken out of the glovebox and equipped with a condenser under argon flow. The reaction was refluxed at 150 °C with stirring. After 24 h, the Schlenk flask was cooled down to room temperature. Then, 0.5 mmol internal standard (benzyl benzoate) was added. The solution

was filtered through Celite, rinsed with ethyl acetate (2 mL) and analyzed by GC to determine the yield of thioester.

Stoichiometric Reaction of Ru-1 Towards Ester

In a N_2 glove box, **Ru-1** (0.003 g, 0.005 mmol) and hexyl hexanoate (2.3 μL, 0.01 mmol), and dioxane (0.6 mL) were added to a J. Young NMR tube. The tube was taken out of the glovebox and rotated at room temperature for 5 h, after which the sample was analyzed by $^{31}P\{^1H\}$ and ^1H NMR spectroscopy, indicating no reaction.

GENERAL COMPUTATIONAL INFORMATION

DFT calculations were performed with Gaussian 16 (C.01 revision)[2] using Truhlar's M06-L functional,[3] the triple-ξ def2-TZVP basis set,[4] W06 density fitting,[5] and Grimme's D3(0) empirical dispersion correction.[6] Frequency calculations at this level of theory were run at 393.15K (experimentally determined internal reaction temperature) to confirm stationary points and transition states and to obtain thermodynamic corrections. Single point energies of the M06-L optimized structures were computed with ORCA (4.2.1)[7] using the range-separated meta-GGA hybrid functional ωB97M-V of the Head-Gordon group[8] including dispersion correction,[9] together with the triple-ξ def2-TZVPP basis set[4] and the corresponding auxiliary basis sets, def2/J[5] and def2-TZVPP/C[10] for RIJCOSX density fitting. The functional and basis set selections are based on recent benchmark studies.[11] The polarizable continuum model (IEFPCM) was used in all calculations (optimization and single point) with the SMD solvation (1,4-dioxane) model of Truhlar and co-workers.[12]

Gibbs free energies were computed by adding the free energy correction term from the frequency calculation to the single point energy in dioxane, according to

$$\mathbf{G^{o\omega B97M\text{-}V}_{(dioxane,393.15K)} = E^{\omega B97M\text{-}V}_{dioxane} + corr^{\mathit{M06\text{-}L}}_{\mathit{freq}(dioxane,1\,atm,393.15K)}}$$

where $\mathbf{E^{\omega B97M\text{-}V}_{dioxane}}$ is the single point energy; and
where $\mathbf{corr^{\mathit{M06\text{-}L}}_{\mathit{freq}}}$ is the thermal correction to the Gibbs free energy from the frequency calculation (at T = 393.15K and P = 1 atm).

Free energy values (G^o) were then corrected to account for changes in standard states (G^o → G). Standard state corrections[13] were employed such that all species are treated as 1M (using an ideal gas approximation), with the exception of H_2 maintained as 1 atm.[14] Other than these standard state corrections, the transformation of hydrogen from the condensed phase to the gas phase is not additionally corrected for in the free energy quantities provided.

Regarding possible conformers of the acridine based ruthenium complexes (in particular the orientations of the ^{i}Pr groups) and geometries of the alkoxide, thiolate, hemiacetaloxide and hemithioacetaloxide ligands, several conformers for each intermediate and transition state were optimized but only the lowest energy results are presented.[15]

Ethanol and ethanethiol were studied as minimal models for the substrates in the system. Note that the various intermediates are numbered **Ru-X**, and the discussed transition states are defined as $\mathbf{TS_{X,Y}}$ with the transition state connecting intermediates **Ru-X** and **Ru-Y**. Directionality of ΔG and ΔG_{TS} values are indicated by the ordering of X,Y and all energies are reported in kcal/mol.

DISCUSSION OF H_2 GAS AND STANDARD STATES

The global transformation of alcohol and thiol to thioester with H_2 evolution is highly dependent on the pressure of H_2 in the system. The computation indicates that $\Delta G_{120^\circ} = +8.1$ kcal/mol. Accounting for standard states[13,14,16] has a large impact on the magnitude of the ΔG value because a) experimental H_2 concentrations will be significantly lower than the concentrations of the other species and b) two molecules of H_2 are generated which has a second order effect on the reaction quotient (Q).

Following the conversion from Cramer,[13] $\Delta G^{o\prime} = \Delta G^{o} + RT \ln(Q^{o\prime}/Q^{o})$, with ideal gas approximations, we find the following:

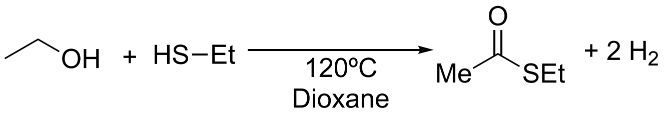

$$\Delta G_{120^\circ,\ 1\ \mathrm{atm}} = +8.1\ \mathrm{kcal/mol}$$

$$\Delta G_{120^\circ,\ 1\mathrm{M}\ (\text{all except H2 1 atm})} = +5.6\ \mathrm{kcal/mol}$$

$$\Delta G_{120^\circ,\ 1\mathrm{M}\ (\text{all except H2 0.003M})} = +1.5\ \mathrm{kcal/mol}$$

Note that additionally, the calculation does not account for a second transformation, which is occurring to the headspace and, in the case of the open system, to the atmosphere:

$$2\ H_{2(\mathrm{diox})} \xrightarrow[\text{Dioxane}]{120^\circ\mathrm{C}} 2\ H_{2(g)}$$

While not computed, this value should be exoergic and drive the overall ΔG to a negative value, in accordance with the experimental findings.

CONCERTED, ASYNCHRONOUS NATURE OF $TS_{3,1}$

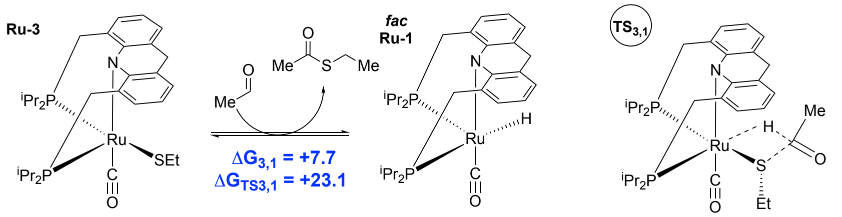

Analysis of the IRC for **$TS_{3,1}$** indicates an interesting asynchronous process is occurring in which the C-S bond forms before hydride elimination occurs.

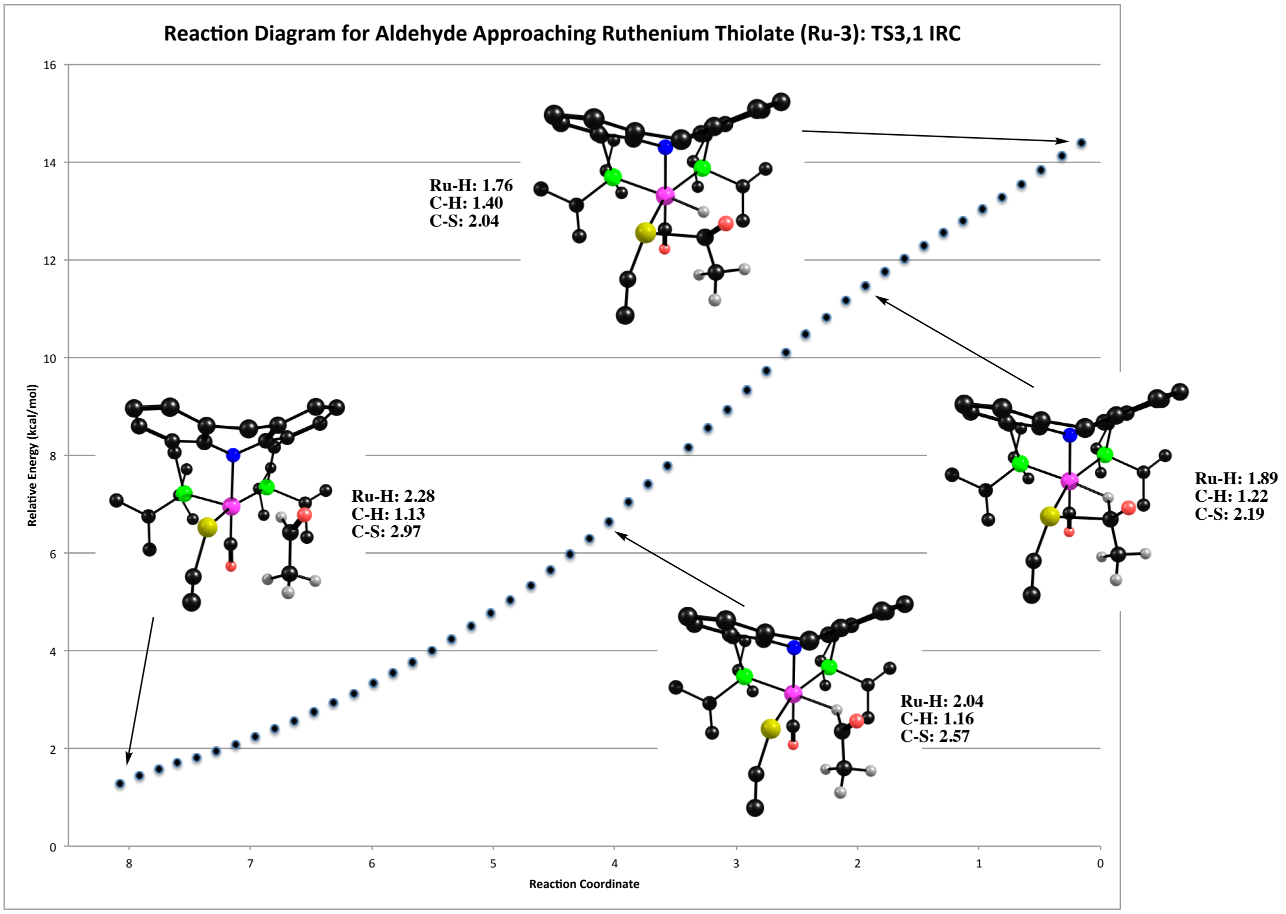

(distances in Å)

$TS_{3,1}$ AND $TS_{7,1}$ WITH THE EXPERIMENTALLY EMPLOYED SUBSTRATES

The transition states associated with thioester formation for the experimentally employed substrates, 3-phenyl-1-propanol and hexanethiol, were calculated. It should be noted that using the larger substrates adds significant computational expense, especially due to the large conformational space associated with the hexyl group, for example. In these two examples, we did not explore the conformational space, and therefore assume there is some additional degree of error in the quantities. Nonetheless, the barriers are similar to those determined using the model computational substrates.

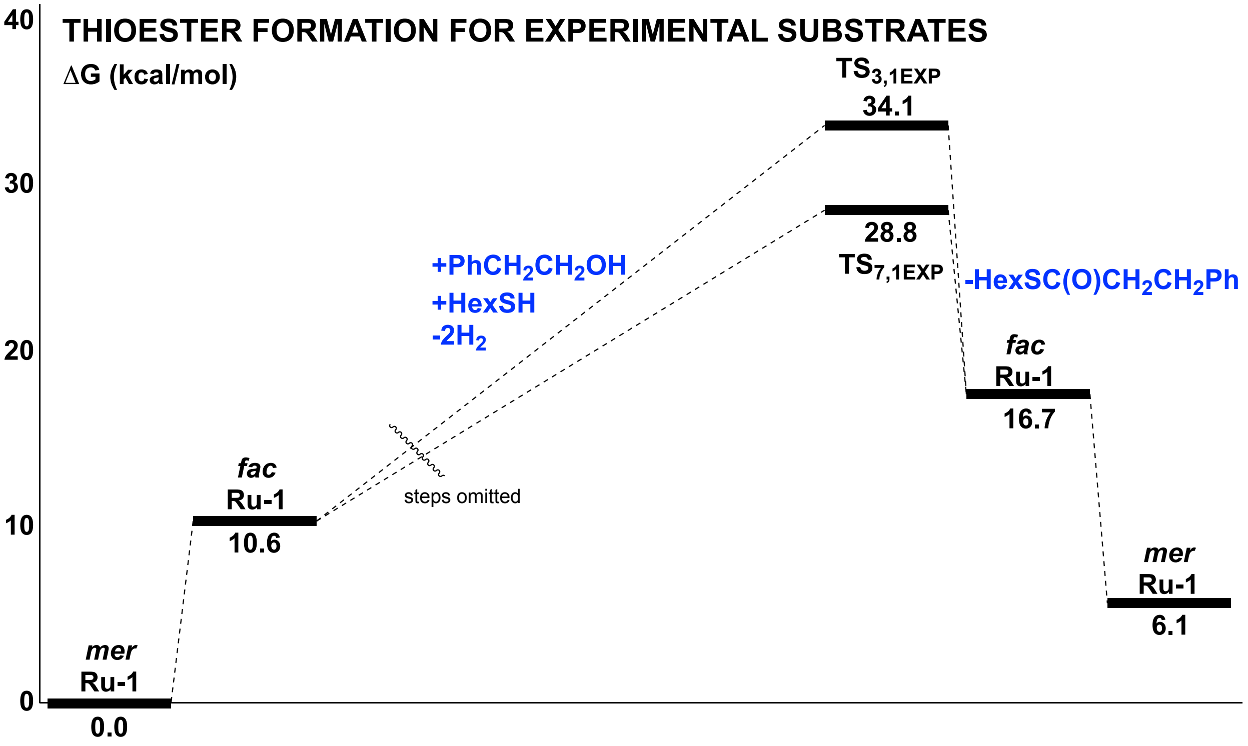

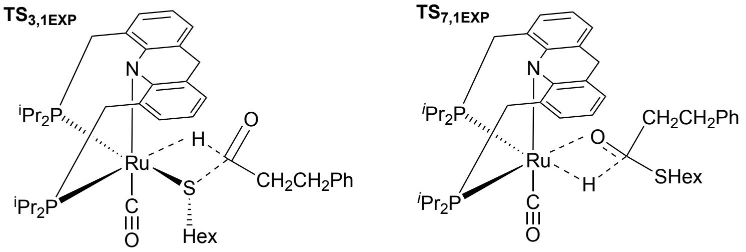

PATHWAY FROM ALDEHYDE TO ESTER

The lowest energy pathway from aldehyde to ester is shown here:

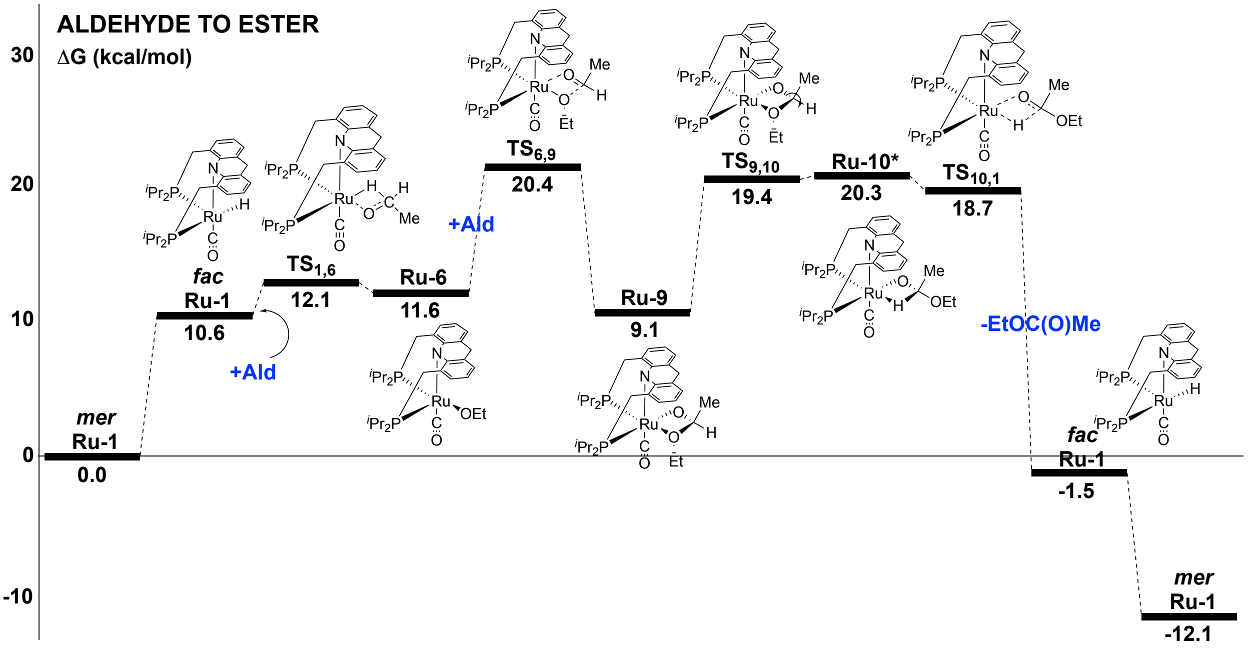

*Note, as discussed in the manuscript, that while **Ru-10** appears 1.6 kcal/mol *above* **TS$_{10,1}$** in the free energy diagram, **Ru-10** is calculated to be slightly lower in energy than the transition state when computed with the M06-L functional used for the optimizations (and confirmed by IRC calculation). The inversion is an erroneous occurrence resulting from the single point energy calculation at the higher level of theory than the originally optimized species in a special case of a very flat potential energy surface. This discrepancy is not chemically meaningful, but highlights the facile nature of the beta hydride elimination.

CARTESIAN COORDINATES

mer Ru-1

Ru	-0.217375000	6.355775000	1.344338000
P	-1.832412000	7.534745000	0.169300000
P	1.691341000	5.987845000	2.623814000
O	-2.160231000	5.207239000	3.282007000
N	0.952229000	6.119283000	-0.475762000
C	-1.391028000	5.707331000	2.560150000
C	-1.637562000	10.108312000	1.277586000
C	-3.247848000	8.530174000	2.364352000
C	-2.613944000	8.965192000	1.052658000
C	-4.053614000	7.458776000	-1.615136000
C	-2.956844000	5.268193000	-1.048278000
C	-3.302177000	6.667658000	-0.557375000
C	3.526360000	4.210755000	3.885355000
C	1.189899000	3.356261000	3.457058000
C	2.306329000	4.274739000	2.983166000
C	0.705580000	6.599447000	5.172430000
C	1.935294000	8.427901000	3.991932000
C	1.839952000	6.926774000	4.215226000
C	0.357304000	6.183708000	-1.753902000
C	2.200107000	5.459872000	-0.475533000
C	-0.894794000	8.320234000	-1.196074000
C	-0.455271000	7.268125000	-2.156764000
C	-0.936769000	7.322058000	-3.461552000
C	-0.673101000	6.328302000	-4.387307000
C	0.069756000	5.232581000	-3.982004000
C	0.564224000	5.151092000	-2.691136000
C	1.304078000	3.954789000	-2.202907000
C	2.458106000	4.410996000	-1.380114000
C	3.706728000	3.817558000	-1.464779000
C	4.737350000	4.215628000	-0.630265000
C	4.479815000	5.194406000	0.313496000
C	3.238809000	5.816050000	0.415184000
C	2.998266000	6.732383000	1.563694000
H	-0.769211000	9.782593000	1.853403000
H	-1.276723000	10.543802000	0.347192000
H	-2.120244000	10.906348000	1.843686000
H	-3.827151000	9.348718000	2.794083000
H	-3.920984000	7.680226000	2.252039000
H	-2.484278000	8.250489000	3.091631000
H	-3.404065000	9.318961000	0.382013000
H	-4.979021000	6.946968000	-1.884713000
H	-4.326853000	8.461310000	-1.286692000
H	-3.463650000	7.552533000	-2.527123000
H	-2.390347000	5.296686000	-1.979137000
H	-2.367939000	4.714355000	-0.314499000

H	-3.868212000	4.699656000	-1.239960000
H	-3.948151000	6.558361000	0.322689000
H	3.951719000	3.205865000	3.875910000
H	4.314811000	4.896389000	3.572501000
H	3.275611000	4.439865000	4.922374000
H	0.816243000	3.626589000	4.445095000
H	0.343040000	3.366519000	2.770853000
H	1.550705000	2.328639000	3.522154000
H	2.601994000	3.934373000	1.985178000
H	-0.247052000	6.962249000	4.783663000
H	0.598429000	5.531564000	5.358270000
H	0.871110000	7.083837000	6.136101000
H	2.014464000	8.940008000	4.952163000
H	2.803626000	8.713377000	3.400284000
H	1.046465000	8.815755000	3.491718000
H	2.785200000	6.592803000	4.654592000
H	-0.046944000	8.810941000	-0.708350000
H	-1.480139000	9.089257000	-1.705097000
H	-1.535416000	8.179927000	-3.747585000
H	-1.051855000	6.401519000	-5.398118000
H	0.266275000	4.416411000	-4.668435000
H	0.630490000	3.354107000	-1.570944000
H	1.617954000	3.308201000	-3.022329000
H	3.859504000	3.024059000	-2.187938000
H	5.715527000	3.758137000	-0.697281000
H	5.258430000	5.494772000	1.006189000
H	3.923268000	6.914595000	2.115163000
H	2.596414000	7.700327000	1.250871000
H	-0.251497000	7.656077000	2.234239000

***fac* Ru-1**

H	0.689882000	8.523731000	5.350577000
P	-2.354266000	7.729516000	2.795216000
P	0.500643000	6.127280000	4.961535000
O	-2.321543000	7.818776000	6.867222000
N	0.731168000	8.355769000	2.735094000
C	-1.576618000	7.873067000	1.135082000
H	-0.913785000	7.007306000	1.050071000
H	-2.328776000	7.798376000	0.346561000
C	-0.786065000	9.130087000	1.002078000
C	-1.149581000	10.102490000	0.077137000
H	-2.040175000	9.942500000	-0.522025000
C	-0.376009000	11.235205000	-0.119921000
H	-0.663925000	11.973813000	-0.856381000
C	0.781891000	11.399163000	0.627907000
H	1.406753000	12.273066000	0.477385000
C	1.149239000	10.470719000	1.588133000

C	0.361001000	9.323268000	1.802566000
C	2.354749000	10.643054000	2.460089000
H	3.076032000	11.326941000	2.009704000
H	2.044522000	11.116249000	3.404460000
C	2.975893000	9.318913000	2.780299000
C	4.336321000	9.146809000	2.978712000
H	5.001494000	9.990529000	2.828312000
C	4.846630000	7.925033000	3.395751000
H	5.909264000	7.800703000	3.557660000
C	3.978010000	6.864696000	3.606561000
H	4.365361000	5.903413000	3.927870000
C	2.612538000	6.997408000	3.384536000
C	2.095577000	8.240244000	2.967224000
C	1.685551000	5.843970000	3.581855000
H	2.247351000	4.926352000	3.761835000
H	1.062536000	5.688205000	2.695169000
C	-3.459725000	6.250780000	2.623081000
H	-4.268773000	6.554542000	1.952959000
C	-4.061803000	5.873996000	3.967241000
H	-3.293017000	5.596013000	4.689529000
H	-4.739353000	5.025222000	3.860860000
H	-4.630402000	6.692487000	4.408522000
C	-2.729552000	5.088832000	1.962331000
H	-2.558448000	5.265753000	0.901785000
H	-3.317682000	4.173875000	2.048778000
H	-1.756045000	4.891663000	2.414772000
C	-3.488681000	9.207880000	2.792497000
H	-2.824407000	9.985661000	2.398091000
C	-3.918712000	9.639429000	4.185738000
H	-4.510721000	8.878221000	4.694881000
H	-4.533725000	10.538972000	4.127421000
H	-3.065821000	9.868915000	4.823966000
C	-4.676346000	9.091622000	1.851930000
H	-4.395716000	8.750032000	0.855078000
H	-5.158371000	10.063423000	1.734407000
H	-5.432925000	8.407489000	2.239511000
C	1.623703000	6.179354000	6.434280000
H	2.357387000	6.917808000	6.089469000
C	0.985250000	6.730690000	7.698262000
H	0.530775000	7.704476000	7.528208000
H	1.748092000	6.854096000	8.468564000
H	0.223444000	6.067144000	8.106257000
C	2.358860000	4.874673000	6.705459000
H	1.697840000	4.107583000	7.109003000
H	3.138457000	5.043288000	7.450055000
H	2.846626000	4.466956000	5.820717000
C	-0.528319000	4.583305000	5.051288000
H	-1.324849000	4.819479000	4.342536000
C	-1.184495000	4.389858000	6.410174000

H	-0.466926000	4.075587000	7.167972000
H	-1.941912000	3.606715000	6.345405000
H	-1.679133000	5.290494000	6.770881000
C	0.149335000	3.308597000	4.569418000
H	0.507003000	3.386700000	3.543915000
H	-0.567718000	2.485868000	4.596444000
H	0.991368000	3.018106000	5.196029000
C	-1.636270000	7.867840000	5.925379000
Ru	-0.561920000	8.003404000	4.449900000

Ru-2

S	-0.846354000	10.631964000	4.430018000
H	-0.627550000	10.949805000	3.136451000
H	0.820600000	8.508798000	5.345124000
P	-2.404577000	7.820406000	2.983480000
P	0.503729000	6.151480000	4.871464000
O	-2.248328000	7.936594000	6.947594000
N	0.779682000	8.353388000	2.640277000
C	-1.649154000	7.759590000	1.303745000
H	-0.991232000	6.884663000	1.314427000
H	-2.412962000	7.598027000	0.542303000
C	-0.862360000	8.989327000	0.992648000
C	-1.319081000	9.883620000	0.031767000
H	-2.263804000	9.678780000	-0.460900000
C	-0.577762000	10.996639000	-0.332138000
H	-0.942049000	11.676185000	-1.091091000
C	0.650513000	11.208032000	0.276592000
H	1.254964000	12.064065000	-0.006234000
C	1.127261000	10.352764000	1.259248000
C	0.365900000	9.230585000	1.655668000
C	2.431447000	10.627324000	1.949150000
H	3.147105000	11.061901000	1.245479000
H	2.290500000	11.410804000	2.707342000
C	3.006192000	9.399178000	2.587853000
C	4.356117000	9.311288000	2.899634000
H	5.003220000	10.148089000	2.656710000
C	4.882298000	8.188308000	3.519848000
H	5.933950000	8.139984000	3.769963000
C	4.045107000	7.113790000	3.784383000
H	4.447107000	6.205261000	4.221708000
C	2.698321000	7.159814000	3.455140000
C	2.143461000	8.334589000	2.899981000
C	1.820439000	5.968092000	3.603965000
H	2.404299000	5.071542000	3.823085000
H	1.285158000	5.800983000	2.666007000
C	-3.487765000	6.305838000	3.078810000
H	-2.801378000	5.555043000	3.468581000

C	-4.021302000	5.774830000	1.757632000
H	-4.676529000	6.481901000	1.250419000
H	-4.605063000	4.869337000	1.936564000
H	-3.222619000	5.507138000	1.066739000
C	-4.591636000	6.461288000	4.114262000
H	-4.230509000	6.889356000	5.050066000
H	-5.023043000	5.486224000	4.347834000
H	-5.404159000	7.090348000	3.751309000
C	-3.538243000	9.289527000	2.818546000
H	-2.817859000	10.058289000	2.510136000
C	-4.162675000	9.746123000	4.131133000
H	-5.041154000	9.156918000	4.388654000
H	-4.489177000	10.784072000	4.048078000
H	-3.471964000	9.690853000	4.970702000
C	-4.590975000	9.200237000	1.724442000
H	-4.182409000	8.893356000	0.762402000
H	-5.059237000	10.175591000	1.577337000
H	-5.387630000	8.504441000	1.988941000
C	1.506533000	6.080238000	6.438279000
H	2.311288000	6.782336000	6.188060000
C	0.806862000	6.613687000	7.676013000
H	0.482861000	7.643479000	7.545595000
H	1.491521000	6.589062000	8.525603000
H	-0.063978000	6.017707000	7.951306000
C	2.128876000	4.720443000	6.715760000
H	1.391211000	4.005832000	7.084571000
H	2.891178000	4.814166000	7.490655000
H	2.612765000	4.281086000	5.843377000
C	-0.346521000	4.504523000	4.775667000
H	0.375649000	3.772680000	5.146080000
C	-0.665672000	4.139007000	3.328987000
H	-0.991266000	4.997861000	2.739231000
H	-1.458879000	3.390572000	3.282330000
H	0.203016000	3.722400000	2.821698000
C	-1.557126000	4.475359000	5.695756000
H	-1.272801000	4.592213000	6.741770000
H	-2.089125000	3.526421000	5.606893000
H	-2.263456000	5.274824000	5.470396000
C	0.704998000	11.349004000	5.069761000
H	0.663662000	11.153505000	6.140949000
H	1.531297000	10.760460000	4.675245000
C	0.827901000	12.818852000	4.764526000
H	0.012877000	13.392302000	5.204334000
H	0.823143000	13.007548000	3.690032000
H	1.765217000	13.207929000	5.162952000
C	-1.577923000	8.006605000	5.997623000
Ru	-0.544233000	8.156231000	4.505969000

Ru-3

S	-0.063829000	10.874657000	3.850354000
P	-2.075859000	7.920163000	2.970538000
P	0.694358000	6.552676000	4.853638000
O	-1.829414000	8.775560000	6.768221000
N	0.909931000	8.373278000	2.335712000
C	-1.546986000	7.812120000	1.215488000
H	-0.948022000	6.896866000	1.161174000
H	-2.432572000	7.666495000	0.596081000
C	-0.750596000	8.971917000	0.724443000
C	-1.199390000	9.793124000	-0.301134000
H	-2.187183000	9.627234000	-0.718996000
C	-0.381983000	10.785756000	-0.822297000
H	-0.734164000	11.414068000	-1.629891000
C	0.898604000	10.953764000	-0.310609000
H	1.546943000	11.722099000	-0.718571000
C	1.356507000	10.176440000	0.741253000
C	0.521699000	9.187665000	1.284203000
C	2.689302000	10.382111000	1.396756000
H	3.408605000	10.824443000	0.704431000
H	2.567254000	11.113934000	2.207618000
C	3.214831000	9.101793000	1.974468000
C	4.568678000	8.825588000	2.069876000
H	5.280118000	9.548732000	1.685155000
C	5.023376000	7.655167000	2.664129000
H	6.084210000	7.456478000	2.742343000
C	4.102332000	6.735613000	3.140392000
H	4.443718000	5.803554000	3.578574000
C	2.735771000	6.964944000	3.027653000
C	2.275479000	8.172931000	2.464478000
C	1.745137000	5.939943000	3.471857000
H	2.254022000	5.020633000	3.763053000
H	1.050525000	5.698285000	2.660097000
C	-2.860273000	6.265933000	3.260872000
H	-1.964086000	5.634791000	3.264892000
C	-3.772225000	5.713315000	2.173153000
H	-4.736729000	6.214121000	2.141607000
H	-3.969805000	4.659562000	2.378141000
H	-3.330467000	5.765810000	1.179995000
C	-3.517687000	6.182905000	4.628519000
H	-2.884150000	6.579241000	5.421588000
H	-3.753240000	5.147206000	4.880401000
H	-4.457006000	6.736936000	4.647925000
C	-3.370790000	9.250741000	2.889746000
H	-2.804529000	10.029885000	2.363229000
C	-3.797479000	9.817446000	4.232363000
H	-4.270685000	9.068619000	4.868432000
H	-4.527528000	10.613449000	4.076563000

H	-2.963374000	10.245916000	4.779737000
C	-4.593443000	8.896372000	2.054812000
H	-4.355090000	8.422364000	1.103925000
H	-5.149387000	9.807293000	1.827898000
H	-5.272800000	8.240274000	2.599365000
C	2.004222000	7.020097000	6.097509000
H	2.653954000	7.640087000	5.468476000
C	1.525065000	7.901341000	7.241145000
H	1.101947000	8.839729000	6.883686000
H	2.370997000	8.156192000	7.881688000
H	0.780198000	7.416512000	7.871174000
C	2.843512000	5.857589000	6.607475000
H	2.294330000	5.237953000	7.316971000
H	3.719676000	6.241810000	7.132749000
H	3.207818000	5.213308000	5.808167000
C	-0.213382000	5.057807000	5.495002000
H	-1.093930000	5.017277000	4.850606000
C	-0.709634000	5.273002000	6.917638000
H	0.102452000	5.206031000	7.641583000
H	-1.437263000	4.503208000	7.180124000
H	-1.195372000	6.238829000	7.053152000
C	0.500886000	3.722225000	5.345498000
H	0.732666000	3.489454000	4.307443000
H	-0.145631000	2.923820000	5.714773000
H	1.426813000	3.671988000	5.915264000
C	-0.836767000	11.907187000	5.145076000
H	-1.816278000	12.226826000	4.783854000
H	-0.999761000	11.318641000	6.047703000
C	0.021992000	13.113886000	5.446397000
H	0.192337000	13.713541000	4.552407000
H	0.996930000	12.819426000	5.834259000
H	-0.456326000	13.752897000	6.190645000
C	-1.247335000	8.695691000	5.764687000
Ru	-0.299473000	8.565028000	4.200346000

Ru-4

S	-0.573574000	10.844117000	3.856705000
H	1.885572000	10.270087000	4.528398000
S	1.872784000	9.286078000	5.452823000
P	-2.148800000	8.060490000	2.988037000
P	0.476038000	6.192821000	4.818157000
O	-1.839022000	8.490044000	6.964672000
N	0.919095000	8.326863000	2.500676000
C	-1.551588000	8.188681000	1.253660000
H	-1.048378000	7.228427000	1.092353000
H	-2.417396000	8.216294000	0.592122000
C	-0.610683000	9.291970000	0.926544000

C	-0.923672000	10.258967000	-0.017534000
H	-1.907800000	10.250127000	-0.474865000
C	0.013483000	11.206866000	-0.401446000
H	-0.236283000	11.953476000	-1.143917000
C	1.279516000	11.177542000	0.166029000
H	2.023644000	11.909032000	-0.131298000
C	1.607697000	10.241135000	1.134475000
C	0.655763000	9.293356000	1.538372000
C	2.930209000	10.234030000	1.838215000
H	3.715580000	10.664765000	1.213280000
H	2.867318000	10.902185000	2.712575000
C	3.300451000	8.853382000	2.291557000
C	4.617128000	8.442723000	2.425642000
H	5.411284000	9.132260000	2.159131000
C	4.929786000	7.180524000	2.912187000
H	5.962029000	6.875815000	3.023714000
C	3.902892000	6.306865000	3.235024000
H	4.133846000	5.305003000	3.581976000
C	2.571535000	6.673994000	3.080049000
C	2.252471000	7.975023000	2.634839000
C	1.467882000	5.704925000	3.347185000
H	1.864095000	4.697145000	3.474145000
H	0.764789000	5.691305000	2.507877000
C	-2.941066000	6.378049000	2.928239000
H	-2.050641000	5.740332000	2.914654000
C	-3.734066000	6.001461000	1.682260000
H	-4.673639000	6.541306000	1.597990000
H	-3.983575000	4.939572000	1.731851000
H	-3.174762000	6.150977000	0.761282000
C	-3.742895000	6.081953000	4.187224000
H	-3.244039000	6.415858000	5.098522000
H	-3.930142000	5.010556000	4.285296000
H	-4.717621000	6.568970000	4.149331000
C	-3.504746000	9.326141000	3.090547000
H	-2.949281000	10.230581000	2.819891000
C	-4.053505000	9.514417000	4.495534000
H	-4.605216000	8.641648000	4.844530000
H	-4.748909000	10.355623000	4.510078000
H	-3.274555000	9.725882000	5.220428000
C	-4.648904000	9.150789000	2.103339000
H	-4.323828000	8.942305000	1.085099000
H	-5.232647000	10.072237000	2.067447000
H	-5.333221000	8.359772000	2.410591000
C	1.771175000	6.086254000	6.154214000
H	2.546411000	6.729673000	5.727143000
C	1.354259000	6.690029000	7.487660000
H	0.748438000	7.586880000	7.373157000
H	2.236760000	6.960013000	8.070652000
H	0.776283000	5.990886000	8.090306000

C	2.400755000	4.716418000	6.358883000
H	1.726253000	4.024470000	6.862864000
H	3.283955000	4.814252000	6.993234000
H	2.727071000	4.251971000	5.429648000
C	-0.692532000	4.776191000	5.125688000
H	-1.555260000	5.039258000	4.521341000
C	-1.176078000	4.738990000	6.567969000
H	-0.420867000	4.337120000	7.242708000
H	-2.051245000	4.091769000	6.648826000
H	-1.465000000	5.722346000	6.939507000
C	-0.226241000	3.409475000	4.645189000
H	-0.032996000	3.393700000	3.573288000
H	-1.007725000	2.672033000	4.839534000
H	0.672636000	3.061946000	5.150794000
C	-1.571882000	11.743003000	5.098923000
H	-2.589163000	11.861966000	4.719437000
H	-1.652302000	11.162733000	6.020836000
C	-0.980277000	13.105221000	5.383542000
H	-0.879168000	13.688755000	4.468310000
H	0.013424000	13.027196000	5.826385000
H	-1.612326000	13.672130000	6.070677000
C	1.361681000	10.387290000	6.809908000
H	0.415193000	10.833558000	6.510249000
H	1.170604000	9.735051000	7.661658000
C	2.405437000	11.428389000	7.124197000
H	2.593135000	12.071896000	6.264614000
H	3.353088000	10.978000000	7.416745000
H	2.067590000	12.062382000	7.944669000
C	-1.263158000	8.451260000	5.952728000
Ru	-0.306854000	8.400807000	4.399812000

Ru-5

O	-0.690138000	10.330779000	4.047582000
H	-0.218890000	10.528397000	3.221224000
H	0.963759000	8.471357000	5.254060000
P	-2.312040000	7.798322000	2.945764000
P	0.568629000	6.078116000	4.848573000
O	-2.050417000	8.032207000	6.930635000
N	0.862041000	8.207745000	2.593766000
C	-1.594549000	7.714163000	1.244774000
H	-0.976655000	6.810536000	1.233650000
H	-2.376300000	7.599673000	0.492733000
C	-0.761785000	8.917401000	0.947911000
C	-1.174617000	9.843211000	-0.002390000
H	-2.110119000	9.670014000	-0.523779000
C	-0.407101000	10.955262000	-0.315627000
H	-0.740931000	11.655685000	-1.069577000

C	0.796383000	11.150770000	0.345373000
H	1.409245000	12.015751000	0.113967000
C	1.229786000	10.264157000	1.320414000
C	0.457645000	9.123417000	1.638905000
C	2.483864000	10.501826000	2.110497000
H	3.195546000	11.102461000	1.540032000
H	2.244861000	11.113011000	2.996337000
C	3.098001000	9.215340000	2.569870000
C	4.456835000	9.081552000	2.813467000
H	5.113525000	9.919498000	2.603618000
C	4.975984000	7.909726000	3.344794000
H	6.035865000	7.820137000	3.543674000
C	4.119702000	6.850658000	3.612046000
H	4.513070000	5.921098000	4.011082000
C	2.761352000	6.943676000	3.343279000
C	2.229297000	8.145725000	2.833906000
C	1.837440000	5.794848000	3.543197000
H	2.385089000	4.877410000	3.768565000
H	1.264707000	5.628846000	2.627731000
C	-3.482443000	6.353628000	3.064123000
H	-2.831495000	5.552019000	3.413171000
C	-4.108218000	5.878803000	1.762403000
H	-4.745629000	6.630211000	1.298862000
H	-4.732669000	5.003745000	1.954405000
H	-3.358768000	5.579820000	1.030249000
C	-4.522576000	6.569634000	4.153916000
H	-4.083259000	6.943012000	5.079882000
H	-5.020120000	5.626573000	4.387209000
H	-5.297105000	7.271948000	3.846423000
C	-3.346373000	9.341583000	2.819515000
H	-2.600799000	10.051137000	2.445267000
C	-3.824098000	9.874825000	4.162156000
H	-4.634727000	9.281407000	4.582814000
H	-4.204256000	10.891467000	4.042184000
H	-3.022225000	9.910107000	4.896527000
C	-4.480517000	9.295519000	1.807422000
H	-4.163937000	8.929576000	0.831105000
H	-4.886064000	10.298222000	1.657134000
H	-5.305775000	8.668937000	2.146834000
C	1.613966000	6.005064000	6.386070000
H	2.436089000	6.670802000	6.096383000
C	0.968229000	6.593966000	7.628060000
H	0.678641000	7.631436000	7.479414000
H	1.674421000	6.564908000	8.459686000
H	0.084127000	6.037384000	7.941356000
C	2.194662000	4.630229000	6.680063000
H	1.443247000	3.951770000	7.087254000
H	2.982112000	4.714667000	7.430519000
H	2.637472000	4.152990000	5.805674000

C	-0.364143000	4.473657000	4.820924000
H	0.322798000	3.721339000	5.216757000
C	-0.713751000	4.069977000	3.392069000
H	-1.017650000	4.920671000	2.778752000
H	-1.533360000	3.349213000	3.382530000
H	0.131771000	3.605088000	2.887575000
C	-1.573685000	4.541790000	5.739562000
H	-1.287906000	4.707326000	6.778584000
H	-2.141514000	3.610355000	5.702026000
H	-2.246581000	5.354024000	5.462914000
C	-0.255552000	11.256663000	5.056989000
H	-0.824102000	10.993897000	5.949568000
H	0.799757000	11.079577000	5.285301000
C	-0.513324000	12.671772000	4.625507000
H	-1.572723000	12.834857000	4.428286000
H	0.040476000	12.915085000	3.716655000
H	-0.199406000	13.371319000	5.399213000
C	-1.402279000	8.026427000	5.961595000
Ru	-0.400153000	8.067577000	4.439503000

Ru-6

Ru	-0.389473000	-0.213857000	0.645286000
P	-1.379438000	1.607320000	-0.301199000
P	-0.706665000	-2.017966000	-0.787004000
O	0.605555000	0.907488000	2.114466000
O	-2.875980000	-0.827355000	2.196535000
N	1.427904000	0.225043000	-0.577516000
C	-0.234468000	2.240728000	-1.593214000
H	-0.166880000	1.445145000	-2.342188000
H	-0.668762000	3.118063000	-2.073300000
C	1.114144000	2.560774000	-1.039191000
C	1.591515000	3.865931000	-1.037765000
H	0.960544000	4.655786000	-1.431679000
C	2.866109000	4.160508000	-0.580934000
H	3.233416000	5.178115000	-0.601148000
C	3.668011000	3.130918000	-0.107045000
H	4.666815000	3.347464000	0.257558000
C	3.208794000	1.824813000	-0.064649000
C	1.917949000	1.519000000	-0.533824000
C	0.735798000	-2.103225000	-1.922212000
H	0.675173000	-3.013618000	-2.522478000
H	0.626547000	-1.246707000	-2.593131000
C	2.044415000	-2.005651000	-1.215199000
C	2.957070000	-3.052503000	-1.215198000
H	2.681597000	-3.990250000	-1.687291000
C	4.222767000	-2.893722000	-0.670315000
H	4.932396000	-3.710434000	-0.687419000

C	4.578849000	-1.663980000	-0.132056000
H	5.576534000	-1.518805000	0.269197000
C	3.677477000	-0.611758000	-0.081188000
C	2.377892000	-0.782345000	-0.594159000
C	4.022251000	0.714378000	0.528835000
H	5.091224000	0.918776000	0.433266000
H	3.834988000	0.679984000	1.611955000
C	-3.054424000	1.506083000	-1.103423000
H	-3.064945000	0.497155000	-1.516840000
C	-3.263746000	2.469529000	-2.263249000
H	-4.261028000	2.320635000	-2.681065000
H	-2.550717000	2.310858000	-3.070951000
H	-3.197104000	3.513251000	-1.958013000
C	-4.196731000	1.584252000	-0.101626000
H	-4.322447000	2.591124000	0.294724000
H	-4.068015000	0.906390000	0.740366000
H	-5.132658000	1.315516000	-0.594386000
C	-1.378417000	2.993248000	0.928170000
H	-0.301249000	3.084618000	1.108569000
C	-2.021984000	2.670427000	2.267723000
H	-1.850563000	3.501269000	2.954326000
H	-1.578613000	1.785318000	2.718146000
H	-3.100018000	2.531527000	2.199244000
C	-1.882651000	4.316500000	0.369371000
H	-2.964861000	4.317956000	0.234730000
H	-1.424880000	4.582299000	-0.582708000
H	-1.649301000	5.120182000	1.069726000
C	-0.496038000	-3.627854000	0.134249000
H	0.580733000	-3.605118000	0.343495000
C	-1.216175000	-3.705263000	1.470353000
H	-2.298292000	-3.618723000	1.370490000
H	-0.885807000	-2.931895000	2.162575000
H	-1.013879000	-4.667629000	1.943407000
C	-0.780620000	-4.860807000	-0.709790000
H	-0.423172000	-5.754035000	-0.195453000
H	-0.290846000	-4.834612000	-1.683191000
H	-1.850292000	-4.998026000	-0.876495000
C	-2.129727000	-2.286383000	-1.938787000
H	-1.997864000	-3.294053000	-2.341569000
C	-3.456104000	-2.248353000	-1.196632000
H	-3.611764000	-1.306112000	-0.670668000
H	-3.527944000	-3.042288000	-0.453090000
H	-4.287796000	-2.378157000	-1.891038000
C	-2.047544000	-1.306991000	-3.106473000
H	-3.021104000	-1.186755000	-3.584377000
H	-1.350437000	-1.651057000	-3.868509000
H	-1.709891000	-0.316636000	-2.796234000
C	1.105964000	-0.260738000	2.575888000
H	0.847627000	-1.160954000	1.861184000

C	-1.909613000	-0.587407000	1.594967000
H	2.207515000	-0.316263000	2.513206000
C	0.616421000	-0.675028000	3.948551000
H	-0.472744000	-0.678903000	3.991475000
H	0.975317000	0.039834000	4.688687000
H	0.980790000	-1.663913000	4.229623000

Ru-7

Ru	10.644562000	3.675601000	2.427239000
O	11.879263000	3.832196000	4.151037000
P	9.403477000	2.118021000	3.660754000
P	9.930042000	3.466217000	0.215016000
O	8.747002000	5.912942000	3.006080000
N	12.033731000	1.966188000	1.931622000
C	10.224844000	0.484366000	3.461876000
H	9.703984000	-0.263981000	4.059731000
H	10.098419000	0.220961000	2.406589000
C	11.673636000	0.524794000	3.816807000
C	12.170896000	-0.185491000	4.902919000
H	11.483188000	-0.756772000	5.517878000
C	13.528793000	-0.206320000	5.181626000
H	13.905514000	-0.775646000	6.021307000
C	14.401026000	0.498262000	4.361116000
H	15.466598000	0.485716000	4.565792000
C	13.930388000	1.241301000	3.291613000
C	12.553088000	1.271815000	3.010956000
C	14.818599000	2.081235000	2.425021000
H	14.846275000	3.104387000	2.832072000
H	15.851521000	1.728489000	2.451209000
C	14.296256000	2.144613000	1.022292000
C	12.898845000	2.132804000	0.863585000
C	15.115030000	2.276757000	-0.086901000
H	16.191208000	2.288080000	0.051176000
C	14.577669000	2.415619000	-1.360266000
H	15.226062000	2.522065000	-2.220113000
C	13.200038000	2.396654000	-1.521516000
H	12.774461000	2.457579000	-2.517516000
C	12.353425000	2.242380000	-0.430521000
C	10.875313000	2.101963000	-0.578228000
H	10.549515000	1.196049000	-0.059331000
H	10.582167000	2.014328000	-1.625639000
C	9.653884000	2.438213000	5.470636000
H	10.742001000	2.334643000	5.531383000
C	9.320997000	3.847888000	5.932746000
H	9.816809000	4.597799000	5.322555000
H	9.672990000	3.982164000	6.957496000
H	8.250955000	4.052035000	5.935415000

C	9.023426000	1.399645000	6.387422000
H	7.938647000	1.500144000	6.438025000
H	9.400870000	1.536248000	7.402475000
H	9.253244000	0.374750000	6.099592000
C	7.597125000	1.789768000	3.372186000
H	7.501277000	1.906714000	2.293246000
C	7.121830000	0.382576000	3.704697000
H	7.631742000	-0.377451000	3.114535000
H	6.056544000	0.299882000	3.480833000
H	7.248295000	0.129940000	4.756185000
C	6.714644000	2.847973000	4.018111000
H	6.645770000	2.711587000	5.096906000
H	5.700499000	2.779672000	3.620684000
H	7.068873000	3.862547000	3.834564000
C	8.179073000	3.129805000	-0.317142000
H	8.128492000	3.467016000	-1.357428000
C	7.869757000	1.636533000	-0.306567000
H	8.356255000	1.115857000	-1.129429000
H	6.795854000	1.470003000	-0.404357000
H	8.191989000	1.143215000	0.611976000
C	7.174068000	3.943024000	0.485768000
H	7.258243000	3.759723000	1.554672000
H	6.155472000	3.689679000	0.186087000
H	7.297174000	5.014678000	0.344718000
C	10.419307000	4.926015000	-0.834033000
H	11.458521000	5.090987000	-0.527193000
C	10.416761000	4.662879000	-2.332257000
H	11.111788000	3.880973000	-2.626636000
H	10.713391000	5.569694000	-2.862150000
H	9.426415000	4.393469000	-2.703241000
C	9.608089000	6.175421000	-0.527121000
H	8.604480000	6.105185000	-0.948566000
H	10.074699000	7.049262000	-0.985613000
H	9.508469000	6.376078000	0.538112000
C	9.483100000	5.037178000	2.783405000
C	12.656844000	4.890629000	3.965048000
S	12.645307000	5.123196000	1.977445000
H	13.722414000	4.694438000	4.175110000
C	12.269474000	6.877617000	1.676222000
H	11.819421000	6.916150000	0.685317000
H	11.507666000	7.211671000	2.380122000
C	13.509645000	7.738733000	1.723484000
H	14.237871000	7.421768000	0.978042000
H	13.999176000	7.699490000	2.695705000
H	13.256284000	8.780459000	1.522933000
C	12.213684000	6.158296000	4.650481000
H	11.185810000	6.420325000	4.399915000
H	12.248716000	5.972016000	5.725436000
H	12.861745000	7.009203000	4.443488000

Ru-9

Ru	10.408317000	3.689089000	2.509719000
O	11.657833000	4.115669000	4.154095000
P	9.308447000	2.065073000	3.655983000
P	9.748892000	3.445984000	0.296972000
O	8.185395000	5.612942000	3.063240000
N	11.965225000	2.150826000	2.019742000
C	10.228119000	0.488448000	3.423396000
H	9.736837000	-0.310587000	3.978580000
H	10.149745000	0.256424000	2.356208000
C	11.659126000	0.611590000	3.829358000
C	12.173357000	-0.112901000	4.897780000
H	11.513306000	-0.768793000	5.455742000
C	13.516984000	-0.036702000	5.230367000
H	13.909700000	-0.618021000	6.054265000
C	14.354186000	0.786992000	4.488444000
H	15.406601000	0.858850000	4.742678000
C	13.863535000	1.544889000	3.438463000
C	12.502701000	1.466028000	3.093928000
C	14.709832000	2.516811000	2.674424000
H	14.648340000	3.498469000	3.172265000
H	15.765737000	2.240077000	2.710022000
C	14.233560000	2.664761000	1.261126000
C	12.854770000	2.510284000	1.026734000
C	15.072619000	3.022953000	0.218145000
H	16.132602000	3.146927000	0.415260000
C	14.574953000	3.253047000	-1.058925000
H	15.238912000	3.549203000	-1.860458000
C	13.221049000	3.068199000	-1.302481000
H	12.826848000	3.196828000	-2.305587000
C	12.362316000	2.666718000	-0.287171000
C	10.935601000	2.324812000	-0.548172000
H	10.727233000	1.329863000	-0.144751000
H	10.719853000	2.306809000	-1.618577000
C	9.542547000	2.368659000	5.469563000
H	10.636298000	2.345275000	5.525752000
C	9.108818000	3.737829000	5.968412000
H	9.585796000	4.535399000	5.404700000
H	9.418270000	3.850715000	7.009081000
H	8.029582000	3.880378000	5.940328000
C	8.990771000	1.258054000	6.353407000
H	7.901212000	1.273021000	6.399103000
H	9.352267000	1.394461000	7.374046000
H	9.299795000	0.262375000	6.038107000
C	7.521203000	1.662999000	3.341389000
H	7.420050000	1.850350000	2.273358000

C	7.144230000	0.207566000	3.578771000
H	7.707278000	-0.477555000	2.946726000
H	6.087423000	0.065541000	3.345380000
H	7.287601000	-0.098772000	4.614423000
C	6.561893000	2.608910000	4.048510000
H	6.518742000	2.419273000	5.120399000
H	5.553238000	2.463049000	3.657910000
H	6.816709000	3.657318000	3.903375000
C	8.124751000	2.831966000	-0.346589000
H	8.152582000	3.026016000	-1.421944000
C	8.006827000	1.319439000	-0.163708000
H	8.510582000	0.782561000	-0.965780000
H	6.961126000	1.007698000	-0.170598000
H	8.446158000	0.965250000	0.770160000
C	6.963333000	3.623229000	0.234317000
H	6.938467000	3.595545000	1.323445000
H	6.011816000	3.229415000	-0.126699000
H	7.008799000	4.673905000	-0.052652000
C	10.069953000	5.035046000	-0.626349000
H	11.166319000	5.053225000	-0.630791000
C	9.606832000	5.010698000	-2.075473000
H	9.912027000	4.111504000	-2.610373000
H	10.029777000	5.861383000	-2.611902000
H	8.521835000	5.095759000	-2.153270000
C	9.592747000	6.297326000	0.073832000
H	8.509840000	6.318917000	0.198098000
H	9.862432000	7.171661000	-0.521780000
H	10.046166000	6.418141000	1.055750000
C	9.060871000	4.874620000	2.855387000
C	12.200733000	5.279187000	3.769751000
O	11.955963000	5.389390000	2.288797000
C	11.602095000	6.507281000	4.420187000
H	11.732924000	6.437931000	5.500137000
H	12.074909000	7.427794000	4.078598000
H	10.534639000	6.572305000	4.210348000
C	13.076057000	5.740887000	1.479457000
H	13.973314000	5.247465000	1.866386000
H	12.895183000	5.323534000	0.487000000
C	13.279215000	7.232389000	1.407875000
H	13.566188000	7.649574000	2.372678000
H	12.373907000	7.739818000	1.074543000
H	14.076486000	7.464447000	0.701195000
H	13.304534000	5.287414000	3.886025000

Ru-10

Ru	-0.354255000	0.469399000	0.116842000
O	0.860098000	1.077263000	1.745575000

P	-1.494946000	-1.011127000	1.385774000
P	-1.010082000	0.186252000	-2.085686000
O	-2.423835000	2.593155000	0.508291000
N	1.179565000	-1.161023000	-0.254382000
C	-0.511840000	-2.551663000	1.455486000
H	-0.995976000	-3.246934000	2.144572000
H	-0.572140000	-2.978793000	0.451074000
C	0.920041000	-2.346433000	1.815264000
C	1.445778000	-2.847658000	2.997571000
H	0.777689000	-3.312208000	3.715775000
C	2.810444000	-2.809600000	3.239955000
H	3.215966000	-3.207442000	4.160863000
C	3.651355000	-2.300188000	2.261509000
H	4.725964000	-2.315712000	2.412434000
C	3.151288000	-1.763097000	1.084055000
C	1.762395000	-1.716158000	0.874105000
C	4.065808000	-1.242290000	0.015488000
H	4.468941000	-0.261938000	0.299856000
H	4.952078000	-1.879437000	-0.059262000
C	3.389363000	-1.153622000	-1.321482000
C	1.979632000	-1.114356000	-1.384529000
C	4.136632000	-1.115677000	-2.488706000
H	5.218925000	-1.143938000	-2.413438000
C	3.533239000	-1.040919000	-3.735104000
H	4.130854000	-1.002818000	-4.636222000
C	2.149939000	-1.056234000	-3.807542000
H	1.658303000	-1.058438000	-4.774778000
C	1.368108000	-1.122238000	-2.660557000
C	-0.114716000	-1.260597000	-2.776909000
H	-0.463300000	-2.123758000	-2.199809000
H	-0.394196000	-1.420034000	-3.818696000
C	-1.492802000	-0.492982000	3.166579000
H	-0.423180000	-0.571049000	3.393559000
C	-1.901147000	0.946785000	3.419574000
H	-1.273456000	1.634802000	2.860850000
H	-1.780304000	1.180020000	4.479151000
H	-2.945013000	1.138014000	3.167311000
C	-2.254550000	-1.448729000	4.073551000
H	-3.334193000	-1.332046000	3.965149000
H	-2.016462000	-1.232371000	5.115897000
H	-2.012311000	-2.497372000	3.902358000
C	-3.196823000	-1.625853000	1.012863000
H	-3.491843000	-2.179444000	1.909123000
C	-4.179501000	-0.481898000	0.823293000
H	-4.263806000	0.134497000	1.717771000
H	-5.174867000	-0.867836000	0.597927000
H	-3.889932000	0.179433000	0.006891000
C	-3.175269000	-2.611844000	-0.151534000
H	-2.514910000	-2.294187000	-0.960399000

H	-4.174811000	-2.731017000	-0.572374000
H	-2.835225000	-3.597061000	0.162749000
C	-2.780432000	-0.003217000	-2.631226000
H	-3.219441000	-0.586029000	-1.821113000
C	-3.520644000	1.326016000	-2.667003000
H	-3.375318000	1.914797000	-1.762502000
H	-4.592405000	1.146909000	-2.768377000
H	-3.219243000	1.938093000	-3.516349000
C	-2.988968000	-0.796971000	-3.912365000
H	-2.515560000	-0.334399000	-4.777316000
H	-4.056984000	-0.862081000	-4.128353000
H	-2.616190000	-1.817245000	-3.832928000
C	-0.246188000	1.575800000	-3.065332000
H	0.817452000	1.371191000	-2.891142000
C	-0.480697000	1.500661000	-4.566806000
H	-0.272716000	0.513916000	-4.979089000
H	0.177017000	2.205804000	-5.078047000
H	-1.502703000	1.769308000	-4.834697000
C	-0.525222000	2.972013000	-2.527679000
H	-1.570562000	3.261547000	-2.626548000
H	0.061153000	3.700336000	-3.090617000
H	-0.248063000	3.083269000	-1.479322000
C	-1.621349000	1.767226000	0.367585000
C	1.398952000	2.009530000	0.961100000
O	0.749680000	3.297728000	0.995368000
C	2.904099000	2.111452000	1.093054000
H	3.314675000	2.915693000	0.484298000
H	3.176449000	2.271914000	2.135487000
H	3.347647000	1.172308000	0.781657000
C	1.117436000	4.170407000	2.050645000
H	2.202161000	4.332178000	2.057293000
H	0.669512000	5.129800000	1.782670000
C	0.638446000	3.738329000	3.414935000
H	1.046591000	2.769758000	3.701133000
H	-0.448362000	3.662955000	3.438831000
H	0.939156000	4.470986000	4.165429000
H	1.215452000	1.750491000	-0.168535000

Ru-11

S	-1.226618000	11.013353000	3.711914000
P	-2.322115000	7.951239000	2.924947000
P	0.622914000	6.826270000	5.094589000
O	-2.109171000	8.935073000	6.884613000
N	0.756924000	8.638228000	2.470554000
C	-1.636543000	7.891463000	1.225523000
H	-0.961633000	7.028418000	1.245345000
H	-2.440736000	7.664157000	0.524729000

C	-0.890530000	9.100209000	0.780757000
C	-1.365379000	9.881945000	-0.262764000
H	-2.323483000	9.635342000	-0.709254000
C	-0.616663000	10.936971000	-0.761760000
H	-0.990837000	11.536429000	-1.581227000
C	0.630365000	11.194152000	-0.212581000
H	1.238214000	12.002473000	-0.605853000
C	1.114967000	10.452413000	0.854558000
C	0.342636000	9.409141000	1.391262000
C	2.432770000	10.770389000	1.494528000
H	3.138862000	11.153739000	0.753340000
H	2.302021000	11.598987000	2.205100000
C	3.009526000	9.580827000	2.203186000
C	4.372518000	9.458317000	2.426660000
H	5.033742000	10.234496000	2.055336000
C	4.897010000	8.379392000	3.123984000
H	5.961957000	8.302804000	3.299245000
C	4.037719000	7.388837000	3.572862000
H	4.433182000	6.518962000	4.087324000
C	2.670631000	7.465321000	3.337806000
C	2.127644000	8.588444000	2.675165000
C	1.766117000	6.350183000	3.742293000
H	2.344689000	5.475094000	4.040045000
H	1.119745000	6.062370000	2.906335000
C	-2.909597000	6.205206000	3.140889000
H	-1.945850000	5.686460000	3.198666000
C	-3.667700000	5.555217000	1.991535000
H	-4.680828000	5.936082000	1.889388000
H	-3.750180000	4.483054000	2.179922000
H	-3.165234000	5.670781000	1.033042000
C	-3.641389000	6.018959000	4.461075000
H	-3.145963000	6.526941000	5.289993000
H	-3.718950000	4.960386000	4.716458000
H	-4.659248000	6.405804000	4.401898000
C	-3.782626000	9.082818000	2.773178000
H	-3.308590000	9.963230000	2.322491000
C	-4.361027000	9.497369000	4.117342000
H	-4.809587000	8.656962000	4.648220000
H	-5.148461000	10.237650000	3.967165000
H	-3.610806000	9.948996000	4.761844000
C	-4.884403000	8.615062000	1.834677000
H	-4.515165000	8.231449000	0.884157000
H	-5.546112000	9.452283000	1.606950000
H	-5.501175000	7.843389000	2.295391000
C	1.806135000	7.161113000	6.488320000
H	2.497780000	7.847635000	5.983151000
C	1.210870000	7.909490000	7.670198000
H	0.712175000	8.828190000	7.368078000
H	2.006868000	8.186370000	8.363137000

H	0.495492000	7.308657000	8.230030000
C	2.610374000	5.956612000	6.952590000
H	2.007384000	5.262963000	7.538333000
H	3.423872000	6.290588000	7.598545000
H	3.062817000	5.401421000	6.131946000
C	-0.324333000	5.280351000	5.512413000
H	-1.146952000	5.308837000	4.798677000
C	-0.941098000	5.346382000	6.902124000
H	-0.195037000	5.209060000	7.684514000
H	-1.676426000	4.549000000	7.020512000
H	-1.452907000	6.289714000	7.091117000
C	0.411879000	3.967608000	5.283895000
H	0.740178000	3.848961000	4.252557000
H	-0.260896000	3.136301000	5.502912000
H	1.281194000	3.851011000	5.927970000
C	0.266667000	10.829470000	4.550969000
H	0.923330000	9.060282000	5.089037000
H	1.155635000	10.889168000	3.925530000
C	-1.482258000	8.856297000	5.911892000
Ru	-0.494378000	8.773058000	4.374432000
C	0.460919000	11.434103000	5.908628000
H	-0.395063000	11.264963000	6.559945000
H	1.357136000	11.048211000	6.397217000
H	0.584880000	12.515357000	5.811959000

Ru-12

S	-0.682552000	10.807210000	3.893051000
H	1.228380000	10.084540000	4.695708000
O	1.543953000	9.300392000	5.205825000
P	-2.243379000	7.989769000	2.866061000
P	0.337121000	6.191365000	4.791946000
O	-2.220666000	8.406076000	6.761689000
N	0.824149000	8.308992000	2.503229000
C	-1.578395000	8.071786000	1.156387000
H	-1.030192000	7.131927000	1.031002000
H	-2.416808000	8.062162000	0.459606000
C	-0.675862000	9.221086000	0.873738000
C	-0.995119000	10.192676000	-0.064966000
H	-1.956868000	10.147065000	-0.565881000
C	-0.086153000	11.187493000	-0.393421000
H	-0.338394000	11.934792000	-1.134281000
C	1.154067000	11.209179000	0.230895000
H	1.872513000	11.982758000	-0.019188000
C	1.482382000	10.275312000	1.200780000
C	0.560236000	9.274020000	1.542345000
C	2.756467000	10.321868000	1.986434000
H	3.551315000	10.824944000	1.431859000

H	2.585293000	10.942828000	2.880995000
C	3.179778000	8.951889000	2.423571000
C	4.506116000	8.609163000	2.624370000
H	5.276941000	9.340317000	2.403225000
C	4.857214000	7.361895000	3.126634000
H	5.896592000	7.108148000	3.288888000
C	3.859764000	6.439029000	3.399615000
H	4.121721000	5.450497000	3.762435000
C	2.521193000	6.740390000	3.175604000
C	2.163258000	8.019779000	2.707484000
C	1.454556000	5.722967000	3.406007000
H	1.894848000	4.742934000	3.590718000
H	0.807398000	5.640725000	2.526202000
C	-3.038951000	6.314669000	2.871158000
H	-2.144803000	5.682368000	2.874773000
C	-3.839391000	5.898246000	1.642974000
H	-4.807563000	6.388728000	1.585878000
H	-4.032047000	4.825016000	1.695975000
H	-3.312373000	6.079074000	0.708271000
C	-3.824891000	6.063231000	4.147810000
H	-3.282518000	6.369827000	5.042389000
H	-4.065167000	5.003333000	4.252272000
H	-4.772578000	6.602972000	4.136084000
C	-3.561754000	9.294651000	2.835957000
H	-2.982863000	10.146224000	2.460961000
C	-4.093909000	9.674568000	4.205283000
H	-4.616291000	8.851420000	4.694321000
H	-4.806251000	10.495583000	4.108297000
H	-3.300064000	10.009409000	4.864977000
C	-4.710862000	9.032159000	1.874136000
H	-4.391678000	8.695917000	0.888674000
H	-5.270623000	9.957509000	1.728820000
H	-5.413479000	8.299749000	2.271992000
C	1.537511000	6.283150000	6.210378000
H	2.257417000	7.002766000	5.803766000
C	0.959153000	6.874183000	7.486995000
H	0.420835000	7.801854000	7.310301000
H	1.763507000	7.091301000	8.192299000
H	0.278144000	6.189574000	7.989594000
C	2.291475000	4.995949000	6.509788000
H	1.656809000	4.258890000	7.002334000
H	3.117902000	5.206913000	7.191177000
H	2.721187000	4.533436000	5.622625000
C	-0.730360000	4.686379000	5.068960000
H	-1.555528000	4.842788000	4.376211000
C	-1.331028000	4.652218000	6.466588000
H	-0.601256000	4.336285000	7.211764000
H	-2.151767000	3.933805000	6.501233000
H	-1.730832000	5.616978000	6.778379000

C	-0.102562000	3.349702000	4.699146000
H	0.178481000	3.300567000	3.648441000
H	-0.826387000	2.551282000	4.873888000
H	0.779508000	3.115566000	5.292322000
C	-1.081030000	11.557105000	5.514208000
H	-2.082532000	11.256481000	5.826637000
H	-0.397248000	11.177157000	6.276356000
C	-0.995528000	13.063762000	5.433626000
H	-1.699459000	13.460013000	4.701941000
H	-0.000084000	13.393161000	5.135034000
H	-1.223788000	13.519759000	6.398706000
C	2.031944000	9.767004000	6.466756000
H	1.198581000	9.959143000	7.153176000
H	2.616885000	8.949712000	6.891661000
C	2.881303000	10.995305000	6.289416000
H	2.305081000	11.812661000	5.851676000
H	3.730038000	10.791543000	5.636784000
H	3.265844000	11.337996000	7.249482000
C	-1.565793000	8.391433000	5.799673000
Ru	-0.504832000	8.360475000	4.309380000

$TS_{2,3'}$

S	-0.541345000	11.115830000	3.414792000
P	-2.159543000	7.893074000	2.881164000
P	0.777467000	6.746070000	4.932424000
O	-1.764365000	9.058603000	6.717419000
N	0.879973000	8.373662000	2.249514000
C	-1.591987000	7.709206000	1.144132000
H	-0.959154000	6.815348000	1.150501000
H	-2.448203000	7.505675000	0.500247000
C	-0.818501000	8.873052000	0.626491000
C	-1.296761000	9.655274000	-0.416837000
H	-2.281056000	9.445101000	-0.822987000
C	-0.518262000	10.662257000	-0.967553000
H	-0.896313000	11.258335000	-1.787792000
C	0.757256000	10.884875000	-0.467099000
H	1.379224000	11.663289000	-0.896358000
C	1.245381000	10.148092000	0.600572000
C	0.450172000	9.142901000	1.176413000
C	2.575977000	10.421900000	1.232558000
H	3.269930000	10.868617000	0.517511000
H	2.437797000	11.178678000	2.019365000
C	3.154704000	9.184810000	1.850305000
C	4.519368000	8.977011000	1.969221000
H	5.200397000	9.710840000	1.550875000
C	5.021485000	7.865461000	2.633616000
H	6.089438000	7.719140000	2.728324000

C	4.137637000	6.941446000	3.168950000
H	4.515598000	6.059622000	3.675916000
C	2.763602000	7.103161000	3.035739000
C	2.253720000	8.243495000	2.382073000
C	1.811622000	6.082970000	3.562748000
H	2.346439000	5.193826000	3.898114000
H	1.102162000	5.778477000	2.785902000
C	-2.913731000	6.234725000	3.231466000
H	-2.008859000	5.616817000	3.272281000
C	-3.806580000	5.613157000	2.167016000
H	-4.777374000	6.098362000	2.101259000
H	-3.992170000	4.567408000	2.419685000
H	-3.355791000	5.624693000	1.176226000
C	-3.579403000	6.206428000	4.598853000
H	-2.974871000	6.687341000	5.369301000
H	-3.767714000	5.179473000	4.917935000
H	-4.544518000	6.713949000	4.574315000
C	-3.488995000	9.173511000	2.711982000
H	-2.934505000	9.957757000	2.181626000
C	-3.939987000	9.758016000	4.039396000
H	-4.416374000	9.016975000	4.682259000
H	-4.668835000	10.552119000	3.868322000
H	-3.108862000	10.191732000	4.588809000
C	-4.684817000	8.779622000	1.858849000
H	-4.411411000	8.283264000	0.928531000
H	-5.252444000	9.672488000	1.591069000
H	-5.366348000	8.124499000	2.402131000
C	2.068734000	7.147341000	6.208448000
H	2.747232000	7.759654000	5.601682000
C	1.587633000	8.016471000	7.359224000
H	1.124783000	8.937471000	7.009910000
H	2.437693000	8.297563000	7.982969000
H	0.874056000	7.505122000	8.004191000
C	2.856075000	5.950986000	6.721748000
H	2.268656000	5.341393000	7.408835000
H	3.729351000	6.298707000	7.276144000
H	3.221178000	5.304529000	5.924741000
C	-0.208698000	5.291702000	5.540820000
H	-1.068142000	5.302211000	4.869334000
C	-0.738195000	5.509749000	6.950477000
H	0.047532000	5.397709000	7.697763000
H	-1.505006000	4.767817000	7.179620000
H	-1.187625000	6.493121000	7.084422000
C	0.451517000	3.927747000	5.396027000
H	0.712705000	3.699261000	4.364047000
H	-0.242484000	3.153809000	5.729365000
H	1.352219000	3.828577000	5.999004000
C	-0.938603000	12.050409000	4.937014000
H	-1.198187000	13.047221000	4.577334000

H	-1.842857000	11.640873000	5.388699000
C	0.176431000	12.128780000	5.950082000
H	1.085813000	12.539877000	5.512081000
H	0.419385000	11.148020000	6.360025000
H	-0.112000000	12.766816000	6.786746000
C	-1.192530000	8.891517000	5.718761000
Ru	-0.271627000	8.658591000	4.151857000
H	1.155566000	9.406404000	4.719631000
H	0.638337000	10.218947000	4.161107000

$TS_{2,3''}$

S	-0.577461000	10.792291000	4.013397000
H	0.791319000	10.957442000	4.164491000
O	2.203106000	10.861718000	5.008965000
P	-2.317376000	7.918531000	3.098477000
P	0.547502000	6.235448000	4.828162000
O	-1.882743000	8.446738000	7.038077000
N	0.767211000	8.327448000	2.529443000
C	-1.707925000	7.734110000	1.373095000
H	-1.078237000	6.838141000	1.379235000
H	-2.546833000	7.551967000	0.700736000
C	-0.916586000	8.915330000	0.919322000
C	-1.376538000	9.746673000	-0.094686000
H	-2.346962000	9.548988000	-0.537993000
C	-0.595791000	10.787821000	-0.572708000
H	-0.958792000	11.420270000	-1.372205000
C	0.664895000	10.996459000	-0.028781000
H	1.289112000	11.800996000	-0.403143000
C	1.139414000	10.207105000	1.006630000
C	0.340102000	9.162323000	1.508234000
C	2.464127000	10.450267000	1.666301000
H	3.161236000	10.930749000	0.976491000
H	2.335573000	11.159078000	2.495753000
C	3.040633000	9.181858000	2.218999000
C	4.404236000	8.977095000	2.358770000
H	5.087305000	9.755704000	2.036265000
C	4.901947000	7.807045000	2.917008000
H	5.968851000	7.664575000	3.028614000
C	4.019119000	6.808538000	3.301470000
H	4.400341000	5.866401000	3.680743000
C	2.648007000	6.975188000	3.152580000
C	2.142795000	8.190454000	2.647858000
C	1.676049000	5.877138000	3.423510000
H	2.180004000	4.924617000	3.592746000
H	1.020866000	5.763044000	2.555739000
C	-3.424046000	6.451772000	3.384881000
H	-2.729498000	5.701886000	3.755680000

C	-4.085093000	5.860616000	2.148417000
H	-4.760921000	6.555125000	1.652326000
H	-4.676270000	4.989214000	2.437370000
H	-3.357160000	5.521330000	1.412709000
C	-4.426830000	6.713289000	4.498964000
H	-3.974172000	7.199102000	5.364571000
H	-4.856914000	5.771097000	4.843029000
H	-5.253598000	7.338623000	4.162778000
C	-3.425681000	9.403115000	2.919899000
H	-2.720072000	10.116674000	2.481433000
C	-3.915124000	9.986178000	4.237309000
H	-4.804749000	9.475551000	4.603344000
H	-4.181246000	11.036764000	4.107909000
H	-3.165517000	9.929977000	5.023648000
C	-4.579632000	9.247799000	1.941442000
H	-4.272000000	8.855167000	0.973578000
H	-5.035523000	10.222927000	1.759422000
H	-5.363663000	8.601597000	2.336190000
C	1.715245000	6.197032000	6.275036000
H	2.314906000	7.097645000	6.108254000
C	1.010904000	6.316791000	7.616939000
H	0.268446000	7.111703000	7.645531000
H	1.738454000	6.518966000	8.404689000
H	0.513723000	5.384353000	7.886285000
C	2.667090000	5.009823000	6.292380000
H	2.142496000	4.056615000	6.373980000
H	3.319067000	5.083181000	7.164354000
H	3.311670000	4.966661000	5.417983000
C	-0.425133000	4.661367000	5.013352000
H	0.259271000	3.955569000	5.493207000
C	-0.784989000	4.078609000	3.648877000
H	-1.137597000	4.829897000	2.940772000
H	-1.572320000	3.329388000	3.747286000
H	0.071329000	3.592185000	3.184692000
C	-1.622783000	4.846998000	5.938903000
H	-1.322554000	4.937076000	6.980769000
H	-2.298634000	3.992545000	5.868953000
H	-2.195450000	5.742761000	5.705889000
C	-0.927862000	11.645870000	5.591103000
H	-1.987302000	11.502455000	5.796650000
H	-0.366563000	11.135836000	6.371302000
C	-0.566786000	13.107485000	5.514300000
H	-1.121059000	13.619025000	4.728194000
H	0.496730000	13.238407000	5.319196000
H	-0.795129000	13.599964000	6.460074000
C	2.233848000	9.741672000	5.599604000
H	1.118652000	8.941635000	5.220953000
H	2.941738000	8.977277000	5.213625000
C	-1.309462000	8.384054000	6.026724000

Ru	-0.397032000	8.338050000	4.441290000
C	2.098060000	9.724091000	7.106336000
H	1.244342000	10.313000000	7.438888000
H	2.013748000	8.721650000	7.519051000
H	2.994203000	10.185571000	7.526629000

$TS_{5,6}$

O	-0.249192000	10.656429000	3.730001000
P	-2.054683000	7.893287000	3.050511000
P	0.809570000	6.525125000	4.942070000
O	-1.653031000	8.791931000	6.902068000
N	0.953497000	8.339588000	2.416787000
C	-1.514530000	7.702771000	1.303211000
H	-0.895573000	6.798972000	1.304995000
H	-2.382580000	7.511171000	0.671410000
C	-0.724196000	8.859249000	0.783450000
C	-1.153921000	9.615216000	-0.300849000
H	-2.102554000	9.376160000	-0.769552000
C	-0.368012000	10.631876000	-0.821302000
H	-0.711540000	11.205020000	-1.672484000
C	0.871274000	10.893484000	-0.251054000
H	1.495252000	11.686096000	-0.650450000
C	1.321944000	10.165668000	0.836904000
C	0.523976000	9.138856000	1.369520000
C	2.620037000	10.450648000	1.529480000
H	3.319252000	10.966318000	0.868521000
H	2.426155000	11.146582000	2.359321000
C	3.219941000	9.193219000	2.082992000
C	4.585269000	8.989885000	2.200460000
H	5.264505000	9.753560000	1.836277000
C	5.089984000	7.840514000	2.796245000
H	6.158367000	7.697997000	2.892338000
C	4.211148000	6.872114000	3.257594000
H	4.594051000	5.960339000	3.704307000
C	2.837107000	7.031476000	3.122196000
C	2.326360000	8.211626000	2.547642000
C	1.879365000	5.971320000	3.551365000
H	2.409523000	5.060792000	3.831731000
H	1.190538000	5.725093000	2.736412000
C	-3.007794000	6.334903000	3.363976000
H	-2.241036000	5.580166000	3.145228000
C	-4.186412000	6.075256000	2.437656000
H	-5.023421000	6.734003000	2.667595000
H	-4.543584000	5.052925000	2.573609000
H	-3.941874000	6.193555000	1.383388000
C	-3.428210000	6.181359000	4.819060000
H	-2.652830000	6.495578000	5.514876000

H	-3.670408000	5.139893000	5.038378000
H	-4.318788000	6.767906000	5.039403000
C	-3.216992000	9.338431000	2.994079000
H	-2.500797000	10.168783000	2.961010000
C	-4.058008000	9.470389000	4.255205000
H	-4.878859000	8.751578000	4.260078000
H	-4.509017000	10.462874000	4.299273000
H	-3.490279000	9.331299000	5.173195000
C	-4.098734000	9.446338000	1.758418000
H	-3.527810000	9.459244000	0.834259000
H	-4.655713000	10.384272000	1.799178000
H	-4.832424000	8.644321000	1.695507000
C	2.085389000	6.830909000	6.261901000
H	2.771760000	7.485240000	5.710069000
C	1.596704000	7.613461000	7.470247000
H	1.175252000	8.576864000	7.190872000
H	2.437025000	7.811299000	8.137700000
H	0.848810000	7.073930000	8.050176000
C	2.867598000	5.597177000	6.689212000
H	2.272291000	4.933173000	7.316367000
H	3.732097000	5.900767000	7.281979000
H	3.245931000	5.017320000	5.848581000
C	-0.179305000	5.023795000	5.411005000
H	-1.037169000	5.094675000	4.738447000
C	-0.700224000	5.115433000	6.838189000
H	0.089956000	4.923574000	7.564106000
H	-1.477015000	4.367425000	7.004245000
H	-1.131245000	6.089035000	7.071050000
C	0.483056000	3.679122000	5.146264000
H	0.731717000	3.537663000	4.095940000
H	-0.204087000	2.877381000	5.423115000
H	1.391687000	3.534791000	5.727988000
C	-0.881897000	11.566109000	4.596439000
H	-1.921967000	11.265042000	4.794823000
H	-0.390593000	11.585522000	5.582926000
C	-0.857717000	12.940098000	3.973758000
H	-1.363430000	12.930467000	3.007105000
H	0.167093000	13.271808000	3.804765000
H	-1.352736000	13.673593000	4.611504000
C	-1.091087000	8.669752000	5.891834000
Ru	-0.175149000	8.510530000	4.309538000
H	1.279497000	9.433840000	4.907776000
H	0.787890000	10.092859000	4.424693000

$TS_{6,1'}$

Ru	-0.378058000	-0.268706000	0.690469000
P	-1.408256000	1.644313000	-0.307185000

P	-0.670320000	-2.072357000	-0.699405000
O	0.607708000	0.987601000	2.175643000
O	-2.829091000	-0.909890000	2.281665000
N	1.436351000	0.223905000	-0.543823000
C	-0.247062000	2.240794000	-1.603842000
H	-0.177067000	1.427713000	-2.333656000
H	-0.660677000	3.112488000	-2.112084000
C	1.099202000	2.559992000	-1.042891000
C	1.568037000	3.868736000	-1.054570000
H	0.931330000	4.647087000	-1.461990000
C	2.837251000	4.182401000	-0.597350000
H	3.195291000	5.203017000	-0.628753000
C	3.646119000	3.164539000	-0.112192000
H	4.643955000	3.391184000	0.248982000
C	3.197555000	1.855298000	-0.056499000
C	1.908177000	1.528414000	-0.520539000
C	0.770057000	-2.135189000	-1.835346000
H	0.718869000	-3.047862000	-2.432650000
H	0.636338000	-1.282694000	-2.506632000
C	2.084097000	-2.007779000	-1.145931000
C	3.008743000	-3.042978000	-1.132969000
H	2.740138000	-3.993721000	-1.582609000
C	4.276956000	-2.858549000	-0.600807000
H	4.996384000	-3.666795000	-0.606880000
C	4.619928000	-1.615311000	-0.087684000
H	5.616714000	-1.450456000	0.308281000
C	3.705259000	-0.572813000	-0.052482000
C	2.406220000	-0.766384000	-0.555435000
C	4.034808000	0.764434000	0.540755000
H	5.098809000	0.986281000	0.428874000
H	3.865572000	0.739470000	1.627253000
C	-3.079955000	1.568734000	-1.121812000
H	-3.093397000	0.563541000	-1.545717000
C	-3.294943000	2.544085000	-2.268983000
H	-4.293436000	2.401619000	-2.686711000
H	-2.584323000	2.392267000	-3.080446000
H	-3.224974000	3.584640000	-1.954420000
C	-4.214023000	1.630215000	-0.109272000
H	-4.352300000	2.636168000	0.285907000
H	-4.060095000	0.958329000	0.734693000
H	-5.152052000	1.342577000	-0.587191000
C	-1.392410000	3.051411000	0.899271000
H	-0.315368000	3.125702000	1.087168000
C	-2.053770000	2.746136000	2.234623000
H	-1.825724000	3.545686000	2.941609000
H	-1.680981000	1.819865000	2.666907000
H	-3.138807000	2.684842000	2.163157000
C	-1.865823000	4.381051000	0.332053000
H	-2.946400000	4.401103000	0.186407000

H	-1.394389000	4.630380000	-0.618035000
H	-1.624595000	5.186054000	1.028583000
C	-0.505876000	-3.708661000	0.173927000
H	0.564198000	-3.694408000	0.417394000
C	-1.268809000	-3.825682000	1.482149000
H	-2.348668000	-3.770603000	1.342184000
H	-0.985182000	-3.052910000	2.193562000
H	-1.056251000	-4.789770000	1.946986000
C	-0.772703000	-4.914300000	-0.714295000
H	-0.427101000	-5.822292000	-0.218230000
H	-0.262306000	-4.862303000	-1.675885000
H	-1.838718000	-5.046353000	-0.906368000
C	-2.103254000	-2.278945000	-1.855108000
H	-2.014676000	-3.293444000	-2.252571000
C	-3.421026000	-2.180473000	-1.102947000
H	-3.523335000	-1.230850000	-0.577036000
H	-3.522854000	-2.968331000	-0.356580000
H	-4.264315000	-2.271236000	-1.789515000
C	-2.002049000	-1.315481000	-3.032614000
H	-2.966004000	-1.218540000	-3.534905000
H	-1.283279000	-1.660837000	-3.773642000
H	-1.689704000	-0.314577000	-2.731389000
C	1.057480000	-0.175046000	2.485038000
H	0.595305000	-1.408659000	1.428962000
C	-1.877174000	-0.666090000	1.659936000
H	2.085421000	-0.421613000	2.172413000
C	0.638938000	-0.816106000	3.776763000
H	-0.423502000	-0.678968000	3.968857000
H	1.188113000	-0.325529000	4.583612000
H	0.882185000	-1.876505000	3.808269000

$TS_{3,2}$

S	-0.521781000	10.805503000	4.012615000
H	0.827839000	11.017460000	4.271354000
O	2.360464000	10.864460000	4.813628000
P	-2.243184000	7.926946000	3.196409000
P	0.553595000	6.196548000	4.865363000
O	-1.840009000	8.419145000	7.085210000
N	0.834350000	8.325981000	2.590673000
C	-1.664295000	7.762617000	1.463268000
H	-1.049967000	6.855580000	1.451906000
H	-2.518060000	7.596627000	0.806030000
C	-0.863720000	8.930268000	0.997391000
C	-1.327892000	9.765077000	-0.012059000
H	-2.313551000	9.588261000	-0.429927000
C	-0.531411000	10.779096000	-0.520530000
H	-0.897886000	11.415964000	-1.314922000

C	0.751564000	10.949948000	-0.017165000
H	1.392192000	11.727330000	-0.419918000
C	1.228424000	10.157533000	1.014435000
C	0.409302000	9.150868000	1.557603000
C	2.579794000	10.354217000	1.630346000
H	3.270311000	10.816301000	0.922158000
H	2.497152000	11.054042000	2.470994000
C	3.125633000	9.058991000	2.148795000
C	4.480913000	8.773648000	2.169198000
H	5.177877000	9.513394000	1.790127000
C	4.954384000	7.567277000	2.669272000
H	6.015729000	7.356698000	2.680484000
C	4.048528000	6.624354000	3.129011000
H	4.401903000	5.657545000	3.471773000
C	2.680995000	6.877164000	3.116189000
C	2.205045000	8.116605000	2.644723000
C	1.697236000	5.816567000	3.476637000
H	2.211086000	4.877113000	3.680719000
H	1.020600000	5.645467000	2.631033000
C	-3.315314000	6.429003000	3.454302000
H	-2.594975000	5.677966000	3.769923000
C	-3.999878000	5.873567000	2.212211000
H	-4.700891000	6.573161000	1.761477000
H	-4.569225000	4.983210000	2.486429000
H	-3.288047000	5.572415000	1.445606000
C	-4.304010000	6.621877000	4.595034000
H	-3.858453000	7.100033000	5.467761000
H	-4.698433000	5.657055000	4.917387000
H	-5.157295000	7.225537000	4.286642000
C	-3.386096000	9.389945000	3.067283000
H	-2.705302000	10.134316000	2.640286000
C	-3.881715000	9.918809000	4.404855000
H	-4.703684000	9.323361000	4.798774000
H	-4.258811000	10.935970000	4.286002000
H	-3.104355000	9.941955000	5.165214000
C	-4.547219000	9.232353000	2.096918000
H	-4.244653000	8.862127000	1.118840000
H	-5.018482000	10.204019000	1.937708000
H	-5.318571000	8.568892000	2.487693000
C	1.593673000	6.080478000	6.403476000
H	1.935490000	7.109772000	6.497203000
C	0.782962000	5.755396000	7.649474000
H	-0.145459000	6.323412000	7.718836000
H	1.370338000	5.983468000	8.540691000
H	0.532131000	4.695584000	7.698562000
C	2.833724000	5.206906000	6.305098000
H	2.603914000	4.159816000	6.109874000
H	3.375375000	5.243714000	7.252659000
H	3.517565000	5.554537000	5.533243000

C	-0.533738000	4.687984000	4.833634000
H	-0.940642000	4.755319000	3.817224000
C	-1.692067000	4.740868000	5.823049000
H	-1.390392000	4.382033000	6.804437000
H	-2.506491000	4.095222000	5.489666000
H	-2.096849000	5.743600000	5.953255000
C	0.183880000	3.349325000	4.936012000
H	0.987261000	3.233489000	4.211177000
H	-0.528223000	2.541970000	4.754575000
H	0.596648000	3.183143000	5.930453000
C	-1.121398000	11.818376000	5.415084000
H	-2.182312000	11.982486000	5.236527000
H	-1.008550000	11.245330000	6.334387000
C	-0.373462000	13.125792000	5.487617000
H	-0.482313000	13.703114000	4.569772000
H	0.690087000	12.962925000	5.659341000
H	-0.756280000	13.728629000	6.311678000
C	2.298424000	9.970116000	5.690321000
H	1.255242000	9.014176000	5.342451000
C	3.497832000	9.066694000	5.860622000
S	1.612293000	10.612210000	7.312026000
C	-1.235739000	8.379587000	6.090361000
Ru	-0.309401000	8.344345000	4.513339000
H	3.410546000	8.370196000	6.690828000
H	3.694276000	8.515655000	4.943052000
H	4.353954000	9.713119000	6.057987000
C	1.312677000	9.118532000	8.285081000
H	0.778201000	8.423119000	7.637366000
H	2.263135000	8.653987000	8.558250000
C	0.496798000	9.424859000	9.519232000
H	-0.453506000	9.891190000	9.262868000
H	1.025782000	10.096723000	10.194679000
H	0.279189000	8.507174000	10.067524000

$TS_{3,1}$

Ru	10.582180000	3.581290000	2.674121000
O	13.335445000	3.728657000	5.006226000
P	9.199666000	2.011790000	3.696514000
P	10.117914000	3.554288000	0.353373000
O	8.728564000	5.863616000	3.235168000
N	11.993643000	1.893673000	2.240320000
C	10.008271000	0.374599000	3.510702000
H	9.416763000	-0.387065000	4.019265000
H	9.980475000	0.159627000	2.437220000
C	11.416181000	0.359222000	4.002912000
C	11.789272000	-0.427229000	5.087720000
H	11.027069000	-0.992951000	5.613716000
C	13.118080000	-0.535656000	5.465366000

H	13.398628000	-1.160606000	6.303181000
C	14.087009000	0.147874000	4.741922000
H	15.132140000	0.061258000	5.019434000
C	13.741215000	0.969114000	3.682622000
C	12.390950000	1.106135000	3.313931000
C	14.738777000	1.783943000	2.921653000
H	14.785972000	2.778094000	3.388091000
H	15.742496000	1.362007000	2.999252000
C	14.321955000	1.939269000	1.492945000
C	12.941206000	2.034262000	1.233419000
C	15.231283000	2.035493000	0.453027000
H	16.290758000	1.962067000	0.674770000
C	14.807475000	2.239079000	-0.853379000
H	15.526010000	2.315940000	-1.658933000
C	13.449514000	2.316337000	-1.116796000
H	13.104229000	2.419835000	-2.139962000
C	12.510124000	2.201955000	-0.097736000
C	11.048123000	2.159813000	-0.394386000
H	10.610941000	1.258939000	0.046442000
H	10.865987000	2.128685000	-1.470156000
C	9.220391000	2.211976000	5.547619000
H	10.292075000	2.071975000	5.739887000
C	8.843376000	3.595726000	6.051602000
H	9.371830000	4.386494000	5.525314000
H	9.096264000	3.682048000	7.109874000
H	7.775863000	3.793313000	5.963535000
C	8.464361000	1.139316000	6.317703000
H	7.384607000	1.269167000	6.239138000
H	8.714304000	1.206589000	7.377913000
H	8.706696000	0.127339000	5.997248000
C	7.439794000	1.732217000	3.167702000
H	7.499177000	1.901670000	2.091426000
C	6.897559000	0.324120000	3.364574000
H	7.475269000	-0.422086000	2.821296000
H	5.874798000	0.274735000	2.985927000
H	6.865262000	0.025356000	4.411039000
C	6.498590000	2.781072000	3.741618000
H	6.280919000	2.591552000	4.792593000
H	5.545903000	2.761354000	3.209676000
H	6.895127000	3.793637000	3.662266000
C	8.420254000	3.417672000	-0.400564000
H	8.515791000	3.880502000	-1.388214000
C	7.992338000	1.971971000	-0.613199000
H	8.580466000	1.477150000	-1.383985000
H	6.947353000	1.931133000	-0.924527000
H	8.081129000	1.369441000	0.292127000
C	7.387271000	4.206166000	0.389620000
H	7.264674000	3.812945000	1.397049000
H	6.414698000	4.156402000	-0.103371000

H	7.648230000	5.257418000	0.492588000
C	10.832380000	5.032984000	-0.524898000
H	11.831508000	5.112448000	-0.081509000
C	10.999692000	4.860835000	-2.026244000
H	11.665770000	4.041435000	-2.284034000
H	11.428532000	5.768815000	-2.453901000
H	10.046852000	4.696081000	-2.532276000
C	10.056997000	6.307122000	-0.226455000
H	9.104093000	6.320905000	-0.757933000
H	10.615765000	7.179508000	-0.569697000
H	9.848232000	6.444739000	0.833957000
C	9.437987000	4.965903000	3.013460000
C	12.477737000	4.455622000	4.558863000
S	12.660496000	4.931953000	2.596013000
C	11.846287000	5.568007000	5.352046000
H	11.544796000	5.163507000	6.317369000
H	12.593699000	6.339138000	5.538107000
H	10.980225000	6.011925000	4.871135000
C	12.351062000	6.723500000	2.479176000
H	12.099742000	6.908217000	1.434786000
H	11.467865000	6.981482000	3.062664000
C	13.560105000	7.531489000	2.886337000
H	14.420444000	7.296150000	2.261335000
H	13.847897000	7.345363000	3.920140000
H	13.355194000	8.597960000	2.785491000
H	11.286086000	3.652729000	4.261167000

$TS_{3,7}$

Ru	10.683713000	3.694499000	2.408531000
O	11.949152000	3.793372000	4.207190000
P	9.400763000	2.121866000	3.637393000
P	9.979646000	3.512150000	0.215817000
O	8.828063000	5.966976000	2.988372000
N	12.049964000	1.979206000	1.937531000
C	10.205188000	0.481143000	3.423355000
H	9.672354000	-0.271566000	4.004885000
H	10.087822000	0.233766000	2.363164000
C	11.650947000	0.503406000	3.792860000
C	12.130594000	-0.229804000	4.872185000
H	11.431181000	-0.806065000	5.469010000
C	13.484901000	-0.267915000	5.165209000
H	13.847144000	-0.855390000	5.998737000
C	14.372748000	0.441434000	4.365366000
H	15.436058000	0.414067000	4.579941000
C	13.920609000	1.206723000	3.303869000
C	12.545861000	1.256461000	3.010145000
C	14.824623000	2.050222000	2.457145000

H	14.842761000	3.072289000	2.866462000
H	15.856433000	1.696258000	2.498489000
C	14.322010000	2.120232000	1.047697000
C	12.927794000	2.132823000	0.875891000
C	15.152767000	2.236978000	-0.054327000
H	16.227723000	2.231147000	0.092702000
C	14.628126000	2.383701000	-1.331893000
H	15.285571000	2.479252000	-2.186128000
C	13.251797000	2.390125000	-1.505449000
H	12.835673000	2.461305000	-2.504737000
C	12.394734000	2.253033000	-0.421129000
C	10.915452000	2.141021000	-0.574451000
H	10.573705000	1.239564000	-0.058410000
H	10.621675000	2.061219000	-1.622160000
C	9.659605000	2.414666000	5.454624000
H	10.744949000	2.288996000	5.512463000
C	9.347160000	3.823440000	5.934458000
H	9.807365000	4.580304000	5.304806000
H	9.742806000	3.958184000	6.943102000
H	8.277598000	4.023558000	5.984225000
C	9.020481000	1.376788000	6.365815000
H	7.937671000	1.490469000	6.424044000
H	9.405725000	1.499905000	7.379781000
H	9.236617000	0.351967000	6.067568000
C	7.585765000	1.812694000	3.370560000
H	7.472322000	1.933681000	2.294717000
C	7.097340000	0.409717000	3.701534000
H	7.589576000	-0.352090000	3.098600000
H	6.028252000	0.342223000	3.490820000
H	7.234103000	0.146736000	4.748906000
C	6.727497000	2.880844000	4.033099000
H	6.659456000	2.733222000	5.110384000
H	5.710789000	2.839650000	3.638571000
H	7.102755000	3.890259000	3.861122000
C	8.218648000	3.147989000	-0.263546000
H	8.142427000	3.479143000	-1.304538000
C	7.929691000	1.650415000	-0.241462000
H	8.388611000	1.137703000	-1.084864000
H	6.854952000	1.469666000	-0.295190000
H	8.296900000	1.159815000	0.661269000
C	7.220640000	3.956073000	0.554296000
H	7.362536000	3.823570000	1.623913000
H	6.199914000	3.651857000	0.314971000
H	7.294230000	5.024159000	0.363308000
C	10.409389000	4.968229000	-0.859190000
H	11.433499000	5.196190000	-0.547704000
C	10.430567000	4.658636000	-2.348840000
H	11.192705000	3.932149000	-2.618384000
H	10.649609000	5.571869000	-2.904704000

H	9.468482000	4.293204000	-2.713200000
C	9.527883000	6.180032000	-0.595945000
H	8.537891000	6.050083000	-1.035719000
H	9.960748000	7.064940000	-1.065625000
H	9.398099000	6.402045000	0.461979000
C	9.546856000	5.079157000	2.759791000
C	12.684175000	4.818550000	4.192715000
S	12.650968000	5.148526000	1.802201000
H	13.771767000	4.665542000	4.285867000
C	12.183219000	6.896395000	1.610543000
H	11.651198000	7.003447000	0.665652000
H	11.479748000	7.180991000	2.394697000
C	13.403549000	7.788160000	1.620357000
H	14.088112000	7.528347000	0.813361000
H	13.958936000	7.704531000	2.554571000
H	13.119428000	8.834185000	1.494525000
C	12.208209000	6.135813000	4.701070000
H	11.182192000	6.351659000	4.408458000
H	12.231237000	6.082166000	5.794312000
H	12.858313000	6.956629000	4.405321000

$TS_{7,1}$

Ru	10.644919000	3.801378000	2.363089000
O	11.847364000	4.286143000	4.116654000
P	9.442485000	2.217423000	3.678918000
P	10.070466000	3.506299000	0.163293000
O	8.405812000	5.767974000	2.598815000
N	12.211605000	2.123324000	2.087214000
C	10.473803000	0.708935000	3.779104000
H	10.004978000	-0.008922000	4.455790000
H	10.454426000	0.282777000	2.772421000
C	11.887488000	0.965595000	4.173980000
C	12.371555000	0.514027000	5.394052000
H	11.680862000	0.054922000	6.094068000
C	13.720949000	0.592004000	5.699928000
H	14.092192000	0.233778000	6.651043000
C	14.592146000	1.084681000	4.740976000
H	15.659956000	1.093725000	4.935452000
C	14.135290000	1.570872000	3.524276000
C	12.754472000	1.592470000	3.249860000
C	15.108050000	2.046510000	2.486169000
H	15.568639000	2.992738000	2.799191000
H	15.951166000	1.350459000	2.426935000
C	14.479803000	2.192983000	1.130061000
C	13.073723000	2.217468000	1.007866000
C	15.274744000	2.307988000	-0.001220000
H	16.353294000	2.295792000	0.119842000

C	14.725116000	2.440204000	-1.266985000
H	15.360527000	2.538486000	-2.137136000
C	13.346034000	2.404101000	-1.401105000
H	12.895619000	2.444821000	-2.387515000
C	12.520551000	2.265098000	-0.293281000
C	11.049822000	2.090373000	-0.467401000
H	10.703365000	1.222549000	0.103520000
H	10.811393000	1.919282000	-1.517505000
C	9.439027000	2.786971000	5.448271000
H	10.509302000	2.738343000	5.676875000
C	8.997825000	4.226668000	5.655656000
H	9.616153000	4.927662000	5.097220000
H	9.088051000	4.486268000	6.712246000
H	7.955878000	4.389959000	5.377516000
C	8.705323000	1.846748000	6.392543000
H	7.622353000	1.939296000	6.294159000
H	8.950938000	2.095346000	7.426053000
H	8.965271000	0.798305000	6.245622000
C	7.753295000	1.526404000	3.363239000
H	7.483725000	1.003839000	4.285505000
C	6.730867000	2.627399000	3.131712000
H	6.660592000	3.306291000	3.980896000
H	5.739536000	2.199081000	2.974884000
H	6.966741000	3.233663000	2.256791000
C	7.780996000	0.490162000	2.244640000
H	8.370929000	0.810037000	1.384416000
H	6.769989000	0.287469000	1.887813000
H	8.201932000	-0.455749000	2.580846000
C	8.312318000	3.124625000	-0.316845000
H	7.956565000	2.566289000	0.550231000
C	7.440463000	4.366939000	-0.431727000
H	7.537869000	5.034209000	0.422267000
H	6.391434000	4.072486000	-0.495597000
H	7.664105000	4.938901000	-1.331655000
C	8.148064000	2.220570000	-1.530328000
H	8.537216000	2.668798000	-2.443782000
H	7.086266000	2.028786000	-1.696071000
H	8.630621000	1.253148000	-1.400864000
C	10.678589000	4.892711000	-0.911852000
H	11.759389000	4.796855000	-0.748488000
C	10.415477000	4.704718000	-2.398840000
H	10.709113000	3.721064000	-2.763164000
H	10.987545000	5.439531000	-2.967660000
H	9.366261000	4.857549000	-2.651952000
C	10.274580000	6.284362000	-0.451780000
H	9.207570000	6.470706000	-0.566724000
H	10.795470000	7.031873000	-1.052209000
H	10.538776000	6.468856000	0.588969000
C	9.294113000	5.023925000	2.524542000

C	12.261177000	5.184469000	3.322307000
S	11.504893000	6.883663000	3.451494000
C	13.709273000	5.198233000	2.915015000
H	13.928485000	5.993368000	2.207032000
H	14.322872000	5.332489000	3.807929000
H	13.970386000	4.246004000	2.469889000
C	12.680558000	7.600950000	4.640839000
H	13.669837000	7.652450000	4.182878000
H	12.338849000	8.631564000	4.745690000
C	12.722050000	6.906579000	5.981038000
H	13.076445000	5.879355000	5.897936000
H	11.734218000	6.871446000	6.439315000
H	13.393681000	7.431511000	6.662450000
H	11.767489000	4.880820000	1.729927000

$TS_{6,9}$

Ru	10.650849000	3.677937000	2.508600000
O	11.916305000	3.811325000	4.324103000
P	9.395233000	2.085259000	3.668341000
P	9.937893000	3.533603000	0.318186000
O	8.789913000	5.926392000	3.175384000
N	12.038140000	2.028078000	1.984226000
C	10.259072000	0.471302000	3.462499000
H	9.750209000	-0.298190000	4.043490000
H	10.157657000	0.216800000	2.402483000
C	11.702393000	0.548749000	3.839730000
C	12.208120000	-0.152123000	4.928101000
H	11.532158000	-0.750594000	5.530171000
C	13.562248000	-0.127626000	5.225754000
H	13.946490000	-0.690026000	6.066684000
C	14.422423000	0.612461000	4.422674000
H	15.484837000	0.632640000	4.643374000
C	13.944112000	1.347605000	3.351029000
C	12.569358000	1.332576000	3.054220000
C	14.816799000	2.227368000	2.506292000
H	14.814691000	3.242769000	2.927958000
H	15.857004000	1.895865000	2.530873000
C	14.301434000	2.314193000	1.103414000
C	12.908049000	2.266645000	0.933997000
C	15.124090000	2.490415000	0.003465000
H	16.198637000	2.527772000	0.149912000
C	14.592987000	2.635781000	-1.272003000
H	15.244849000	2.774742000	-2.124588000
C	13.217875000	2.579734000	-1.444831000
H	12.797616000	2.644528000	-2.442759000
C	12.370418000	2.382559000	-0.361615000
C	10.897611000	2.206646000	-0.513966000

H	10.592384000	1.279314000	-0.021669000
H	10.600491000	2.147169000	-1.561995000
C	9.612289000	2.357541000	5.497200000
H	10.697749000	2.247683000	5.577726000
C	9.266200000	3.750685000	5.998463000
H	9.770796000	4.528734000	5.431781000
H	9.586230000	3.849542000	7.037419000
H	8.196804000	3.955387000	5.975996000
C	8.976668000	1.290854000	6.378175000
H	7.891210000	1.381446000	6.421615000
H	9.343333000	1.402041000	7.400295000
H	9.217276000	0.276148000	6.064651000
C	7.594255000	1.728052000	3.369153000
H	7.494274000	1.874636000	2.295236000
C	7.146009000	0.302659000	3.657321000
H	7.678754000	-0.428614000	3.050996000
H	6.085171000	0.203299000	3.419277000
H	7.266794000	0.021998000	4.702091000
C	6.690742000	2.751908000	4.042097000
H	6.610808000	2.580124000	5.114809000
H	5.682018000	2.684598000	3.630879000
H	7.033028000	3.776945000	3.896532000
C	8.186952000	3.185097000	-0.196032000
H	8.127562000	3.554266000	-1.225009000
C	7.904141000	1.686322000	-0.237227000
H	8.367462000	1.213913000	-1.101676000
H	6.830580000	1.502137000	-0.301200000
H	8.273321000	1.157455000	0.642589000
C	7.178887000	3.967898000	0.632946000
H	7.299896000	3.796910000	1.699695000
H	6.160513000	3.681002000	0.364323000
H	7.265067000	5.041649000	0.480265000
C	10.394513000	5.022801000	-0.691325000
H	11.425099000	5.199598000	-0.374027000
C	10.406181000	4.785468000	-2.194631000
H	11.135829000	4.040784000	-2.500909000
H	10.666424000	5.715054000	-2.703803000
H	9.429828000	4.481761000	-2.576578000
C	9.555938000	6.251050000	-0.372171000
H	8.569746000	6.183701000	-0.834540000
H	10.032779000	7.143488000	-0.780693000
H	9.412509000	6.417688000	0.693464000
C	9.501985000	5.044424000	2.911212000
C	12.438150000	4.935231000	4.214277000
O	12.414143000	4.901434000	2.078735000
C	13.825594000	5.204715000	4.654872000
H	13.800049000	5.768140000	5.590805000
H	14.383718000	4.285077000	4.810312000
H	14.333901000	5.835268000	3.924920000

C	12.529513000	6.217077000	1.638059000
H	11.604071000	6.596864000	1.183585000
H	12.713180000	6.901724000	2.488762000
C	13.676942000	6.344514000	0.658912000
H	13.516009000	5.719279000	-0.221665000
H	14.608802000	6.008823000	1.116891000
H	13.811458000	7.376161000	0.327979000
H	11.811989000	5.829769000	4.070939000

$TS_{9,10}$

Ru	-0.512475000	0.587617000	0.217141000
O	0.531725000	1.029237000	1.997516000
P	-1.602679000	-1.049294000	1.359218000
P	-1.144727000	0.306433000	-2.004275000
O	-2.760430000	2.509792000	0.630994000
N	1.110864000	-0.961369000	-0.188409000
C	-0.528884000	-2.533231000	1.396636000
H	-0.993701000	-3.282659000	2.040863000
H	-0.541945000	-2.918854000	0.373477000
C	0.882673000	-2.278870000	1.799852000
C	1.418491000	-2.826057000	2.956438000
H	0.767596000	-3.371618000	3.632210000
C	2.775945000	-2.727591000	3.225727000
H	3.189459000	-3.164985000	4.124966000
C	3.602169000	-2.098788000	2.305166000
H	4.671877000	-2.053417000	2.482729000
C	3.089557000	-1.509515000	1.158225000
C	1.703892000	-1.547538000	0.916476000
C	3.975468000	-0.808868000	0.167701000
H	4.219597000	0.200452000	0.528563000
H	4.942027000	-1.315965000	0.098506000
C	3.338691000	-0.719068000	-1.188202000
C	1.934194000	-0.788508000	-1.287430000
C	4.104834000	-0.566699000	-2.333128000
H	5.184224000	-0.511570000	-2.234533000
C	3.521025000	-0.480266000	-3.589141000
H	4.132407000	-0.353844000	-4.472932000
C	2.144686000	-0.600158000	-3.697647000
H	1.676735000	-0.598341000	-4.676471000
C	1.349210000	-0.780116000	-2.572617000
C	-0.114175000	-1.043875000	-2.706152000
H	-0.385504000	-1.940830000	-2.139100000
H	-0.375285000	-1.215516000	-3.750129000
C	-1.682823000	-0.605586000	3.160568000
H	-0.617437000	-0.623695000	3.414032000
C	-2.192617000	0.792737000	3.466497000
H	-1.561308000	1.552720000	3.013884000

H	-2.174953000	0.954518000	4.545775000
H	-3.221237000	0.947702000	3.138807000
C	-2.406543000	-1.644807000	4.004531000
H	-3.488789000	-1.592325000	3.872639000
H	-2.208121000	-1.457891000	5.060853000
H	-2.092630000	-2.668010000	3.798728000
C	-3.248730000	-1.780319000	0.939982000
H	-3.502362000	-2.385272000	1.814835000
C	-4.323109000	-0.713203000	0.797220000
H	-4.482913000	-0.178375000	1.733125000
H	-5.275431000	-1.165169000	0.515180000
H	-4.077941000	0.035281000	0.044772000
C	-3.142719000	-2.733916000	-0.249929000
H	-2.445399000	-2.389850000	-1.014987000
H	-4.114068000	-2.865764000	-0.729245000
H	-2.798408000	-3.718654000	0.061572000
C	-2.890702000	-0.078317000	-2.528095000
H	-3.264371000	-0.659953000	-1.687737000
C	-3.766645000	1.162287000	-2.621047000
H	-3.650627000	1.827141000	-1.765639000
H	-4.816744000	0.867678000	-2.663306000
H	-3.561548000	1.736497000	-3.523975000
C	-3.025172000	-0.949820000	-3.767824000
H	-2.608969000	-0.481058000	-4.659110000
H	-4.082289000	-1.135775000	-3.967773000
H	-2.547106000	-1.921123000	-3.648495000
C	-0.560928000	1.751336000	-3.022985000
H	0.509760000	1.733085000	-2.781929000
C	-0.701687000	1.574354000	-4.528053000
H	-0.304550000	0.628226000	-4.890711000
H	-0.158214000	2.367798000	-5.044504000
H	-1.742779000	1.647008000	-4.844349000
C	-1.108769000	3.104947000	-2.596130000
H	-2.116664000	3.276144000	-2.969238000
H	-0.484324000	3.904393000	-2.999566000
H	-1.134958000	3.225648000	-1.516379000
C	-1.875916000	1.767197000	0.483929000
C	1.641410000	1.576198000	1.488953000
O	1.251983000	2.061045000	0.087220000
C	2.259602000	2.617060000	2.384630000
H	3.059246000	3.174356000	1.897233000
H	1.518241000	3.305044000	2.783880000
H	2.703955000	2.088022000	3.227562000
C	1.405372000	3.420916000	-0.291647000
H	2.443745000	3.718743000	-0.111227000
H	1.278676000	3.429621000	-1.376341000
C	0.453496000	4.399782000	0.353171000
H	0.685560000	4.585821000	1.398588000
H	-0.577113000	4.053072000	0.304078000

H	0.503597000	5.356310000	-0.169167000
H	2.409216000	0.843022000	1.213339000

$TS_{10,1}$

Ru	10.671597000	3.714501000	2.394744000
O	11.941540000	4.247088000	4.103711000
P	9.457508000	2.143667000	3.681976000
P	10.088393000	3.477015000	0.200795000
O	8.592925000	5.830091000	2.746687000
N	12.205484000	2.031141000	2.091594000
C	10.436919000	0.597013000	3.734687000
H	9.947751000	-0.125698000	4.391482000
H	10.400363000	0.202046000	2.715766000
C	11.858629000	0.799182000	4.132099000
C	12.338403000	0.276839000	5.325792000
H	11.641279000	-0.205082000	6.003784000
C	13.689598000	0.314774000	5.630964000
H	14.056477000	-0.101233000	6.560044000
C	14.567033000	0.850559000	4.700825000
H	15.634573000	0.840026000	4.896713000
C	14.115108000	1.409004000	3.514273000
C	12.735514000	1.453137000	3.236800000
C	15.086619000	1.957669000	2.512335000
H	15.491476000	2.915536000	2.865183000
H	15.964122000	1.306521000	2.450944000
C	14.476119000	2.117329000	1.149766000
C	13.071049000	2.146291000	1.018536000
C	15.279133000	2.246897000	0.026056000
H	16.356941000	2.228945000	0.153062000
C	14.736766000	2.402238000	-1.240756000
H	15.377658000	2.511505000	-2.105615000
C	13.358102000	2.376723000	-1.383848000
H	12.914546000	2.439231000	-2.372294000
C	12.523915000	2.221726000	-0.284477000
C	11.051436000	2.066686000	-0.471154000
H	10.692023000	1.186828000	0.072780000
H	10.817170000	1.928007000	-1.527123000
C	9.491753000	2.663940000	5.466766000
H	10.564164000	2.586428000	5.678455000
C	9.086981000	4.107919000	5.715221000
H	9.716940000	4.803197000	5.164748000
H	9.194453000	4.340146000	6.776579000
H	8.046662000	4.303189000	5.451705000
C	8.749448000	1.718916000	6.399171000
H	7.667868000	1.843366000	6.321780000
H	9.018214000	1.932783000	7.434784000
H	8.977836000	0.668157000	6.220276000

C	7.741262000	1.518254000	3.371041000
H	7.453546000	0.996629000	4.288270000
C	6.765876000	2.663726000	3.154082000
H	6.719015000	3.328417000	4.016229000
H	5.758167000	2.281592000	2.982444000
H	7.036030000	3.276001000	2.293147000
C	7.724025000	0.494694000	2.240835000
H	8.320742000	0.802362000	1.380643000
H	6.704258000	0.332756000	1.887957000
H	8.112812000	-0.469417000	2.564051000
C	8.326421000	3.147197000	-0.301130000
H	7.958788000	2.556422000	0.538953000
C	7.484485000	4.413893000	-0.352282000
H	7.606262000	5.037794000	0.531872000
H	6.427297000	4.151427000	-0.420982000
H	7.717897000	5.022644000	-1.225308000
C	8.143254000	2.304528000	-1.555020000
H	8.538460000	2.786411000	-2.448326000
H	7.078085000	2.137861000	-1.726709000
H	8.609831000	1.324206000	-1.469732000
C	10.727854000	4.882797000	-0.834899000
H	11.806363000	4.752379000	-0.680542000
C	10.455012000	4.753999000	-2.326273000
H	10.717833000	3.775187000	-2.725579000
H	11.046953000	5.490384000	-2.872573000
H	9.410050000	4.946788000	-2.569798000
C	10.366872000	6.270305000	-0.327854000
H	9.303931000	6.488900000	-0.424128000
H	10.900340000	7.021600000	-0.912769000
H	10.645846000	6.413648000	0.714941000
C	9.399566000	5.005980000	2.613670000
C	12.330521000	5.169970000	3.324763000
O	11.691202000	6.406732000	3.423719000
C	13.770913000	5.226839000	2.893616000
H	13.951431000	6.030484000	2.183077000
H	14.406698000	5.372508000	3.768918000
H	14.042438000	4.286183000	2.430500000
C	12.391487000	7.407053000	4.168228000
H	13.364742000	7.612722000	3.709164000
H	11.787258000	8.305266000	4.040953000
C	12.539773000	7.059894000	5.626509000
H	13.160434000	6.176420000	5.776142000
H	11.569678000	6.864124000	6.081565000
H	13.006589000	7.886600000	6.162087000
H	11.831665000	4.811071000	1.825206000

$TS_{6,1''}$

Ru	10.532420000	3.667956000	2.446897000
O	13.491869000	4.400957000	4.309197000
P	9.348100000	2.138712000	3.657425000
P	9.824686000	3.435369000	0.212991000
O	8.495174000	5.779437000	3.043897000
N	11.989545000	2.042808000	1.960141000
C	10.179048000	0.512476000	3.433722000
H	9.657100000	-0.251168000	4.011002000
H	10.066059000	0.270469000	2.371716000
C	11.623906000	0.557587000	3.808078000
C	12.109911000	-0.177396000	4.883144000
H	11.417871000	-0.771275000	5.471084000
C	13.463043000	-0.191855000	5.181119000
H	13.833828000	-0.777835000	6.011916000
C	14.338383000	0.544218000	4.392883000
H	15.400052000	0.539782000	4.616565000
C	13.883196000	1.311078000	3.333144000
C	12.508308000	1.335542000	3.032692000
C	14.800479000	2.175173000	2.522426000
H	14.888496000	3.141495000	3.034284000
H	15.807359000	1.752158000	2.487588000
C	14.272855000	2.407656000	1.141053000
C	12.882060000	2.339513000	0.941314000
C	15.104693000	2.726279000	0.078792000
H	16.174425000	2.783491000	0.253156000
C	14.595513000	2.992364000	-1.186322000
H	15.257585000	3.258518000	-1.999956000
C	13.231362000	2.872705000	-1.400655000
H	12.820230000	3.015879000	-2.395054000
C	12.377241000	2.514034000	-0.364386000
C	10.935560000	2.231602000	-0.607647000
H	10.680238000	1.261708000	-0.172312000
H	10.710315000	2.193450000	-1.675604000
C	9.616414000	2.431498000	5.476062000
H	10.706839000	2.320725000	5.526037000
C	9.281611000	3.828205000	5.974734000
H	9.738945000	4.605875000	5.367121000
H	9.658736000	3.948929000	6.991564000
H	8.209748000	4.017390000	6.008058000
C	9.000069000	1.372040000	6.379108000
H	7.914760000	1.461025000	6.432540000
H	9.378889000	1.495323000	7.395004000
H	9.239908000	0.355383000	6.071974000
C	7.533809000	1.833074000	3.384766000
H	7.424917000	2.003777000	2.314765000
C	7.069126000	0.409942000	3.658312000
H	7.576940000	-0.320787000	3.030352000
H	6.001716000	0.330593000	3.443722000
H	7.209730000	0.112645000	4.696636000

C	6.651860000	2.856988000	4.084434000
H	6.603333000	2.687698000	5.159490000
H	5.631563000	2.781518000	3.704332000
H	6.983070000	3.881512000	3.921148000
C	8.153586000	2.876821000	-0.353790000
H	8.152175000	3.030118000	-1.436275000
C	7.978362000	1.379380000	-0.108052000
H	8.443294000	0.791186000	-0.897442000
H	6.920991000	1.111011000	-0.081812000
H	8.423649000	1.046488000	0.830857000
C	7.049892000	3.740036000	0.237437000
H	7.082211000	3.774550000	1.326393000
H	6.068236000	3.360995000	-0.051696000
H	7.114772000	4.769844000	-0.113500000
C	10.187583000	4.974365000	-0.773164000
H	11.283864000	4.946465000	-0.798770000
C	9.689732000	4.921074000	-2.209643000
H	9.949056000	3.993372000	-2.719379000
H	10.130690000	5.736579000	-2.784764000
H	8.607017000	5.043598000	-2.265116000
C	9.771897000	6.273929000	-0.104556000
H	8.689944000	6.358442000	0.001534000
H	10.102416000	7.123162000	-0.705333000
H	10.208321000	6.387475000	0.885730000
C	9.292977000	4.964451000	2.810721000
C	12.502514000	4.909323000	3.804824000
O	12.418822000	4.883094000	2.283879000
C	11.918227000	6.192619000	4.353464000
H	11.682673000	6.050367000	5.405522000
H	12.679290000	6.971775000	4.294438000
H	11.026115000	6.528377000	3.831016000
C	12.899082000	6.024086000	1.577872000
H	12.727019000	5.802209000	0.522003000
H	12.303633000	6.911953000	1.825597000
C	14.364376000	6.263717000	1.830500000
H	14.951365000	5.387480000	1.559432000
H	14.566818000	6.493646000	2.875768000
H	14.708582000	7.105312000	1.229055000
H	11.430729000	4.018959000	3.930521000

TS$_{1,9}$

P	-2.325659000	7.804637000	2.944371000
P	0.374119000	6.057623000	4.899883000
O	-2.392871000	7.752307000	6.898289000
N	0.776908000	8.380335000	2.747318000
C	-1.582895000	8.074582000	1.289207000
H	-0.931074000	7.211235000	1.130594000

H	-2.369207000	8.042482000	0.532019000
C	-0.773319000	9.318369000	1.167717000
C	-1.167089000	10.353558000	0.332187000
H	-2.124141000	10.282472000	-0.174788000
C	-0.336690000	11.440745000	0.101129000
H	-0.647191000	12.238299000	-0.560977000
C	0.913929000	11.468584000	0.700312000
H	1.591206000	12.292824000	0.500346000
C	1.320823000	10.466880000	1.570411000
C	0.456506000	9.394243000	1.858481000
C	2.656660000	10.524407000	2.251490000
H	3.410563000	10.939698000	1.576496000
H	2.615730000	11.241518000	3.085945000
C	3.096805000	9.184332000	2.761522000
C	4.431620000	8.927884000	3.037049000
H	5.160159000	9.713046000	2.861541000
C	4.845195000	7.700335000	3.533984000
H	5.889065000	7.517391000	3.752109000
C	3.902806000	6.699751000	3.713729000
H	4.214168000	5.717688000	4.054653000
C	2.560769000	6.913547000	3.424208000
C	2.126702000	8.186867000	2.987820000
C	1.576186000	5.795639000	3.532529000
H	2.093953000	4.844522000	3.660746000
H	0.972462000	5.732541000	2.621426000
C	-3.271070000	6.234215000	2.682329000
H	-4.064892000	6.486969000	1.974289000
C	-3.930492000	5.769206000	3.972240000
H	-3.219318000	5.670104000	4.792498000
H	-4.411614000	4.799639000	3.831982000
H	-4.698543000	6.466969000	4.304664000
C	-2.390704000	5.176916000	2.018141000
H	-2.343771000	5.319332000	0.939793000
H	-2.784592000	4.174850000	2.196562000
H	-1.360975000	5.193297000	2.379442000
C	-3.601285000	9.150895000	3.014997000
H	-3.000427000	10.019338000	2.725745000
C	-4.144499000	9.422127000	4.408113000
H	-4.671557000	8.564025000	4.827332000
H	-4.859309000	10.246327000	4.368868000
H	-3.350771000	9.710310000	5.092070000
C	-4.728209000	8.979077000	2.008276000
H	-4.376866000	8.753461000	1.001206000
H	-5.308276000	9.901043000	1.944355000
H	-5.421973000	8.190562000	2.305492000
C	1.522722000	6.095921000	6.366827000
H	2.242680000	6.847471000	6.020880000
C	0.913653000	6.619944000	7.658283000
H	0.430318000	7.586110000	7.526604000

H	1.699896000	6.750642000	8.404459000
H	0.181368000	5.936220000	8.085470000
C	2.291251000	4.805080000	6.609414000
H	1.657345000	4.022287000	7.025989000
H	3.089019000	4.983495000	7.332705000
H	2.760627000	4.412354000	5.708316000
C	-0.621931000	4.489906000	5.014721000
H	-1.447346000	4.691656000	4.333559000
C	-1.235471000	4.295463000	6.393729000
H	-0.497257000	3.980968000	7.130781000
H	-1.996522000	3.514153000	6.351767000
H	-1.718565000	5.197102000	6.769070000
C	0.065109000	3.227271000	4.517613000
H	0.378093000	3.307259000	3.477355000
H	-0.630500000	2.387852000	4.578341000
H	0.939412000	2.960294000	5.109324000
C	-1.707174000	7.881128000	5.968531000
Ru	-0.607421000	8.102106000	4.528737000
O	-1.141997000	10.211604000	4.306317000
H	0.726972000	10.956325000	3.952930000
C	-0.198204000	11.066117000	4.542929000
H	-1.002655000	12.806672000	3.656348000
C	-0.641393000	12.497453000	4.637264000
H	0.173072000	13.163693000	4.919878000
H	-1.455961000	12.597178000	5.352724000
O	0.419125000	10.696906000	6.009865000
H	0.783351000	8.550313000	5.488065000
H	0.542929000	9.474298000	5.746078000
H	2.260895000	11.354740000	5.327425000
C	1.711638000	11.247281000	6.272232000
H	1.571373000	12.250565000	6.683617000
C	2.471130000	10.368579000	7.229473000
H	2.691490000	9.395561000	6.788071000
H	3.420742000	10.832951000	7.491395000
H	1.909646000	10.209077000	8.149691000

$TS_{6,3}$

S	-0.577270000	10.837814000	3.895140000
H	0.706210000	10.372971000	4.416832000
O	1.409809000	9.256648000	5.100602000
P	-2.192303000	7.884642000	2.881365000
P	0.434557000	6.199831000	4.838362000
O	-2.136286000	8.352269000	6.800671000
N	0.861853000	8.276794000	2.508822000
C	-1.523674000	7.959842000	1.168957000
H	-0.912309000	7.059110000	1.069288000
H	-2.350919000	7.884194000	0.460690000

C	-0.674105000	9.149915000	0.890852000
C	-1.032585000	10.122346000	-0.032161000
H	-1.993238000	10.047883000	-0.531817000
C	-0.159425000	11.152452000	-0.352532000
H	-0.440819000	11.898969000	-1.083686000
C	1.085184000	11.206105000	0.261316000
H	1.777023000	12.003695000	0.011413000
C	1.453600000	10.273000000	1.218486000
C	0.563997000	9.239599000	1.557647000
C	2.741127000	10.349862000	1.982388000
H	3.514573000	10.860305000	1.404358000
H	2.583059000	10.973779000	2.875912000
C	3.198799000	8.993270000	2.426545000
C	4.533535000	8.685360000	2.629695000
H	5.286071000	9.433993000	2.404149000
C	4.916114000	7.451159000	3.140873000
H	5.961925000	7.225578000	3.303645000
C	3.943132000	6.506101000	3.427071000
H	4.229979000	5.528897000	3.801524000
C	2.598219000	6.774955000	3.203515000
C	2.209520000	8.037930000	2.718663000
C	1.547328000	5.743729000	3.444147000
H	1.995838000	4.767668000	3.629000000
H	0.898101000	5.656552000	2.567270000
C	-3.197644000	6.326671000	2.802362000
H	-4.001183000	6.552929000	2.097808000
C	-3.848669000	6.015802000	4.145030000
H	-3.157495000	6.093141000	4.984383000
H	-4.264362000	5.006493000	4.151101000
H	-4.669547000	6.701541000	4.353099000
C	-2.392428000	5.179047000	2.189837000
H	-2.441567000	5.203376000	1.102458000
H	-2.783985000	4.212151000	2.510474000
H	-1.333865000	5.204059000	2.450438000
C	-3.467895000	9.242636000	2.823220000
H	-2.870193000	10.073936000	2.432114000
C	-4.005751000	9.650203000	4.182635000
H	-4.598455000	8.860471000	4.645688000
H	-4.655089000	10.521734000	4.083357000
H	-3.210944000	9.908930000	4.875065000
C	-4.613136000	8.990649000	1.853927000
H	-4.284823000	8.649772000	0.872783000
H	-5.170235000	9.915934000	1.699462000
H	-5.321871000	8.258782000	2.244527000
C	1.626051000	6.258161000	6.258835000
H	2.321733000	7.020650000	5.890216000
C	1.037023000	6.752269000	7.572326000
H	0.393884000	7.619432000	7.448662000
H	1.842223000	7.041220000	8.249647000

H	0.461906000	5.980274000	8.079687000
C	2.406510000	4.970782000	6.484768000
H	1.778810000	4.186040000	6.908551000
H	3.209475000	5.153913000	7.201149000
H	2.870930000	4.581591000	5.580476000
C	-0.675612000	4.729389000	5.096761000
H	-1.518183000	4.957070000	4.449983000
C	-1.230570000	4.639839000	6.510168000
H	-0.493116000	4.256047000	7.214042000
H	-2.076339000	3.949934000	6.527712000
H	-1.586664000	5.598896000	6.886483000
C	-0.107315000	3.396638000	4.632182000
H	0.133285000	3.394595000	3.569993000
H	-0.848123000	2.611002000	4.794312000
H	0.790559000	3.107583000	5.177013000
C	-1.085609000	11.654415000	5.452989000
H	-2.160791000	11.518723000	5.560771000
H	-0.612158000	11.131958000	6.283249000
C	-0.734578000	13.122946000	5.442573000
H	-1.237005000	13.645845000	4.629461000
H	0.336268000	13.281697000	5.320257000
H	-1.036505000	13.593112000	6.379462000
C	1.769141000	9.661972000	6.388524000
H	0.894190000	9.804264000	7.046003000
H	2.380636000	8.889328000	6.877938000
C	2.575935000	10.939977000	6.330469000
H	1.994117000	11.759293000	5.903710000
H	3.459599000	10.801303000	5.706386000
H	2.907149000	11.246000000	7.323922000
C	-1.473414000	8.326618000	5.844408000
Ru	-0.425105000	8.307405000	4.346520000

$TS_{3,11}$

S	-1.141579000	11.012490000	3.726302000
P	-2.311245000	7.931603000	2.919152000
P	0.620165000	6.817605000	5.072771000
O	-2.151542000	8.840100000	6.859719000
N	0.746482000	8.619485000	2.467392000
C	-1.621985000	7.835938000	1.222618000
H	-0.930715000	6.986678000	1.264623000
H	-2.419864000	7.585774000	0.522452000
C	-0.895790000	9.053687000	0.768968000
C	-1.377431000	9.822726000	-0.281052000
H	-2.327881000	9.558326000	-0.733669000
C	-0.641229000	10.885839000	-0.779503000
H	-1.018135000	11.474975000	-1.604593000
C	0.597014000	11.165904000	-0.221556000

H	1.193846000	11.982697000	-0.613471000
C	1.085254000	10.437584000	0.852002000
C	0.327111000	9.383627000	1.384638000
C	2.388667000	10.781431000	1.507539000
H	3.094885000	11.183978000	0.776832000
H	2.228255000	11.603716000	2.219707000
C	2.983404000	9.601556000	2.218423000
C	4.346257000	9.499061000	2.449881000
H	4.997686000	10.286503000	2.085715000
C	4.883896000	8.424363000	3.143920000
H	5.948686000	8.363461000	3.323705000
C	4.039010000	7.417174000	3.581826000
H	4.446270000	6.549412000	4.090636000
C	2.672292000	7.474960000	3.339627000
C	2.116332000	8.592549000	2.680642000
C	1.776885000	6.346451000	3.727930000
H	2.360503000	5.474064000	4.024079000
H	1.135210000	6.064406000	2.885891000
C	-2.947242000	6.204251000	3.162886000
H	-1.996831000	5.659358000	3.210322000
C	-3.744666000	5.566072000	2.032625000
H	-4.746492000	5.978979000	1.948179000
H	-3.856505000	4.498633000	2.229887000
H	-3.257375000	5.663293000	1.064746000
C	-3.659868000	6.049037000	4.498206000
H	-3.130297000	6.544294000	5.312733000
H	-3.768759000	4.995135000	4.760461000
H	-4.663830000	6.471937000	4.455155000
C	-3.734177000	9.105893000	2.742936000
H	-3.219023000	9.965446000	2.296032000
C	-4.319270000	9.550608000	4.075522000
H	-4.788365000	8.725677000	4.612510000
H	-5.090790000	10.302476000	3.905412000
H	-3.565597000	9.996329000	4.719688000
C	-4.835165000	8.670479000	1.786592000
H	-4.460184000	8.263303000	0.848432000
H	-5.461873000	9.528008000	1.538921000
H	-5.487094000	7.925675000	2.242942000
C	1.797512000	7.172434000	6.468328000
H	2.475779000	7.869539000	5.959045000
C	1.188743000	7.911436000	7.650586000
H	0.659631000	8.811020000	7.344810000
H	1.979653000	8.214537000	8.337844000
H	0.493130000	7.293223000	8.215809000
C	2.626304000	5.984606000	6.935484000
H	2.034229000	5.278312000	7.517385000
H	3.430190000	6.333019000	7.585133000
H	3.090714000	5.440622000	6.114337000
C	-0.305297000	5.258966000	5.499896000

H	-1.130360000	5.274371000	4.787372000
C	-0.920779000	5.324704000	6.891263000
H	-0.168808000	5.199364000	7.669678000
H	-1.646963000	4.520390000	7.016688000
H	-1.438527000	6.265441000	7.076907000
C	0.448280000	3.954202000	5.276809000
H	0.777057000	3.837906000	4.245712000
H	-0.208522000	3.112775000	5.503531000
H	1.322035000	3.859272000	5.918198000
C	0.328339000	10.782770000	4.608892000
H	0.884095000	9.163511000	5.072121000
H	1.233666000	10.857559000	4.008237000
C	-1.519765000	8.798505000	5.888063000
Ru	-0.514064000	8.745495000	4.357834000
C	0.477607000	11.378760000	5.979898000
H	-0.395086000	11.184569000	6.600831000
H	1.365627000	11.002804000	6.490741000
H	0.582063000	12.461586000	5.894695000

Ethanethiol

S	-0.807923000	11.015079000	3.619222000
C	-0.616461000	13.084004000	5.495933000
C	-1.378699000	11.831970000	5.141938000
H	-1.027782000	13.541849000	6.396974000
H	0.434667000	12.868292000	5.687858000
H	-0.660732000	13.818846000	4.693380000
H	-1.360474000	11.108866000	5.956961000
H	-2.428434000	12.052954000	4.948079000
H	0.442917000	10.775175000	4.036857000

Ethanol

O	-0.786070000	11.162655000	3.910596000
C	-0.588498000	13.039128000	5.458054000
C	-1.310593000	11.771718000	5.080679000
H	-1.042203000	13.502734000	6.334248000
H	0.457742000	12.841048000	5.698724000
H	-0.612353000	13.760844000	4.641916000
H	-1.306405000	11.065302000	5.921237000
H	-2.357926000	11.978796000	4.854904000
H	0.143385000	10.974809000	4.076845000

Aldehyde

O	-1.312495000	11.416020000	3.837503000
C	-0.621759000	13.069988000	5.422470000
C	-1.231426000	11.788720000	4.978763000

H	-1.359614000	13.652713000	5.977211000
H	-1.631518000	11.157023000	5.806282000
H	0.188760000	12.864887000	6.124396000
H	-0.246137000	13.651598000	4.584745000

Thioester

S	0.815089000	0.200323000	-0.184987000
O	-1.306672000	1.340776000	0.886404000
C	0.145847000	-2.371062000	-0.853565000
C	-0.423909000	-1.122686000	-0.225402000
C	-0.154238000	1.492109000	0.564965000
C	0.602287000	2.769998000	0.755049000
H	1.623831000	2.718238000	0.385866000
H	-0.609420000	-3.156001000	-0.870195000
H	0.465105000	-2.198384000	-1.880734000
H	1.003128000	-2.749833000	-0.298196000
H	-0.751212000	-1.298336000	0.798646000
H	-1.285126000	-0.748697000	-0.777582000
H	0.076826000	3.575310000	0.242996000
H	0.619363000	3.017675000	1.816029000

Hydrogen

H	0.054068000	-0.004785000	0.000000000
H	0.795232000	-0.070375000	0.000000000

Ester

O	0.165688000	0.086952000	-0.030117000
O	-1.572024000	1.064601000	1.007156000
C	0.221481000	-2.144413000	-0.846977000
C	-0.616234000	-1.117860000	-0.147307000
C	-0.446232000	1.117718000	0.579412000
C	0.456680000	2.304924000	0.645470000
H	0.771344000	2.598526000	-0.354839000
H	-0.339631000	-3.071467000	-0.953307000
H	0.509581000	-1.810455000	-1.842828000
H	1.129768000	-2.364255000	-0.287575000
H	-0.914870000	-1.440728000	0.852029000
H	-1.532293000	-0.889183000	-0.695492000
H	-0.050004000	3.135756000	1.124708000
H	1.360053000	2.061411000	1.203013000

Hemithioacetal

S	0.734038000	0.228588000	-0.244855000

O	-1.462052000	1.838678000	-0.144341000
C	0.504406000	2.743640000	0.776514000
C	-0.309247000	1.493784000	0.584548000
C	-0.427375000	-1.164711000	-0.204745000
C	0.245448000	-2.434997000	-0.667958000
H	-2.043624000	1.071640000	-0.171410000
H	-0.590986000	1.061369000	1.552651000
H	1.397003000	2.543085000	1.364785000
H	-0.090279000	3.495284000	1.293496000
H	-1.281695000	-0.940132000	-0.848245000
H	-0.799986000	-1.274251000	0.816736000
H	1.084439000	-2.698612000	-0.025294000
H	0.624452000	-2.337606000	-1.684802000
H	-0.459323000	-3.265962000	-0.656434000
H	0.808099000	3.155975000	-0.184847000

Thioaldehyde

S	-1.367941000	11.250271000	3.537255000
C	-0.617135000	13.086863000	5.462505000
C	-1.225510000	11.811662000	5.044594000
H	-1.348735000	13.679601000	6.018880000
H	-1.609975000	11.209563000	5.873540000
H	0.195265000	12.894392000	6.168894000
H	-0.240160000	13.668597000	4.625702000

Hexanethiol

S	-0.802424000	11.026846000	3.626640000
H	0.446783000	10.783180000	4.046753000
C	-1.374876000	11.841254000	5.147783000
H	-2.425947000	12.063066000	4.956115000
H	-1.357003000	11.119698000	5.965651000
C	-0.618364000	13.100434000	5.512445000
H	-0.650967000	13.799994000	4.673257000
H	0.439401000	12.858583000	5.655338000
C	-1.161808000	13.764092000	6.766880000
H	-2.217608000	14.016341000	6.617887000
H	-1.145125000	13.043460000	7.589989000
C	-0.393354000	15.015214000	7.157449000
H	0.646425000	14.744270000	7.370340000
H	-0.351057000	15.688655000	6.296604000
C	-0.972907000	15.763092000	8.348656000
H	-0.396283000	16.677809000	8.505135000
H	-1.987749000	16.093319000	8.106695000
C	-0.996254000	14.959768000	9.633876000
H	-0.002692000	14.584232000	9.886306000
H	-1.660501000	14.097798000	9.566626000

H	-1.339247000	15.560929000	10.475527000

3-phenyl-1-propanol

O	-0.178949000	11.214804000	3.990115000
C	-0.949895000	13.048075000	5.422609000
C	-1.143375000	11.623615000	4.948248000
H	-1.697453000	13.260003000	6.192154000
H	0.022427000	13.128429000	5.921592000
H	-1.153053000	10.940248000	5.806266000
H	-2.109651000	11.516510000	4.450134000
H	0.684604000	11.259661000	4.414086000
C	-1.041262000	14.059240000	4.296964000
H	-0.342469000	13.771543000	3.506690000
H	-2.029418000	13.980943000	3.830336000
C	-0.795222000	15.496271000	4.663515000
C	-0.606799000	15.921008000	5.975621000
C	-0.758771000	16.460837000	3.654649000
C	-0.388828000	17.260764000	6.270586000
C	-0.543280000	17.796405000	3.943710000
C	-0.356210000	18.203963000	5.257908000
H	-0.628817000	15.201454000	6.784520000
H	-0.901544000	16.150192000	2.625216000
H	-0.244014000	17.564883000	7.299911000
H	-0.519031000	18.523483000	3.141401000
H	-0.185744000	19.248022000	5.487454000

Thioester (exptl)

O	0.215895000	10.331431000	3.165474000
C	0.703262000	12.068776000	4.749586000
C	0.785199000	10.682044000	4.168346000
H	0.520739000	11.991536000	5.824402000
H	1.694956000	12.523250000	4.662893000
C	-0.346373000	12.941806000	4.076159000
H	-0.131121000	12.995201000	3.008313000
H	-1.321140000	12.459610000	4.166580000
C	-0.390297000	14.317628000	4.670989000
C	0.428805000	15.333097000	4.182850000
C	-1.218892000	14.601382000	5.754019000
C	0.418711000	16.595092000	4.756108000
C	-1.232558000	15.861965000	6.330627000
C	-0.412720000	16.863745000	5.833201000
H	1.077843000	15.129423000	3.337910000
H	-1.865048000	13.822567000	6.144608000
H	1.059051000	17.372332000	4.358033000
H	-1.888184000	16.063738000	7.168510000
H	-0.424617000	17.849729000	6.279788000

S	1.847861000	9.594419000	5.110717000
C	1.686821000	8.089730000	4.119228000
H	1.691996000	8.401462000	3.074207000
H	2.600574000	7.522085000	4.298743000
C	0.453226000	7.271140000	4.439248000
H	-0.434286000	7.893728000	4.304014000
H	0.466752000	6.986131000	5.494687000
C	0.346388000	6.030884000	3.569034000
H	1.244579000	5.416703000	3.694809000
H	0.339278000	6.328089000	2.515256000
C	-0.884309000	5.194397000	3.866269000
H	-0.878892000	4.900420000	4.921431000
H	-1.780435000	5.811377000	3.741163000
C	-1.001920000	3.953982000	2.998589000
H	-0.105555000	3.339348000	3.125512000
H	-1.005274000	4.250939000	1.945544000
C	-2.234537000	3.128791000	3.300877000
H	-3.147534000	3.706190000	3.147020000
H	-2.241091000	2.787430000	4.337308000
H	-2.298609000	2.244652000	2.667134000

TS$_{3,1EXP}$

Ru	10.531897000	3.231464000	2.889660000
O	13.265965000	2.961549000	5.198780000
P	9.130678000	1.513247000	3.593228000
P	10.114182000	3.604131000	0.579560000
O	8.670346000	5.397928000	3.782979000
N	11.974150000	1.650266000	2.199199000
C	9.960288000	-0.062007000	3.146138000
H	9.364080000	-0.904104000	3.498401000
H	9.958767000	-0.084679000	2.050919000
C	11.356759000	-0.159627000	3.661293000
C	11.703215000	-1.117595000	4.608092000
H	10.927893000	-1.759143000	5.014259000
C	13.022229000	-1.295230000	4.993640000
H	13.281930000	-2.052491000	5.721859000
C	14.008295000	-0.506927000	4.414628000
H	15.046987000	-0.645276000	4.695694000
C	13.689568000	0.480800000	3.498625000
C	12.347988000	0.688809000	3.128778000
C	14.714970000	1.397701000	2.910152000
H	14.788423000	2.284699000	3.553479000
H	15.705696000	0.938733000	2.912948000
C	14.323396000	1.828360000	1.532110000
C	12.948947000	1.974456000	1.262321000

C	15.258801000	2.118267000	0.552727000
H	16.312214000	2.001910000	0.785007000
C	14.869615000	2.561960000	-0.703306000
H	15.608512000	2.787175000	-1.461198000
C	13.518363000	2.683775000	-0.983318000
H	13.199042000	2.969725000	-1.979800000
C	12.553723000	2.384165000	-0.027719000
C	11.102155000	2.383005000	-0.371207000
H	10.671395000	1.412294000	-0.107964000
H	10.950543000	2.536418000	-1.441266000
C	9.101016000	1.393203000	5.451809000
H	10.169263000	1.232369000	5.646332000
C	8.692969000	2.668049000	6.173152000
H	9.219620000	3.542450000	5.798200000
H	8.922671000	2.578112000	7.236485000
H	7.624739000	2.865695000	6.095744000
C	8.341440000	0.197993000	6.008235000
H	7.262011000	0.328008000	5.926349000
H	8.565505000	0.085529000	7.070478000
H	8.605869000	-0.742062000	5.526495000
C	7.384767000	1.315026000	2.984043000
H	7.461024000	1.673485000	1.957184000
C	6.857859000	-0.111020000	2.916252000
H	7.453531000	-0.742361000	2.258645000
H	5.841958000	-0.101889000	2.516435000
H	6.812827000	-0.593220000	3.891410000
C	6.416270000	2.235900000	3.711833000
H	6.172058000	1.857893000	4.704421000
H	5.478867000	2.306470000	3.157376000
H	6.802619000	3.249015000	3.828030000
C	8.431617000	3.507849000	-0.216545000
H	8.512519000	4.105431000	-1.130959000
C	8.082479000	2.083021000	-0.629567000
H	8.671860000	1.748007000	-1.481148000
H	7.031323000	2.018979000	-0.915231000
H	8.244085000	1.359487000	0.171390000
C	7.354908000	4.132026000	0.659614000
H	7.309495000	3.664855000	1.641102000
H	6.374088000	4.019409000	0.193724000
H	7.514655000	5.194562000	0.828215000
C	10.757216000	5.239264000	-0.040248000
H	11.715272000	5.344241000	0.482110000
C	11.029998000	5.282524000	-1.535439000
H	11.814113000	4.594021000	-1.838417000
H	11.353086000	6.284971000	-1.821833000
H	10.136684000	5.059784000	-2.122294000
C	9.851180000	6.398786000	0.350484000
H	8.962302000	6.429694000	-0.281767000
H	10.370913000	7.347147000	0.204431000

H	9.514896000	6.360240000	1.386581000
C	9.380686000	4.542384000	3.432390000
C	12.416456000	3.768450000	4.925544000
S	12.579251000	4.638144000	3.097416000
C	11.765560000	4.694925000	5.921343000
H	10.908367000	5.181851000	5.465754000
H	11.376615000	4.071770000	6.728446000
C	12.055050000	6.380383000	3.240304000
H	11.371516000	6.551981000	2.412210000
H	11.465649000	6.512021000	4.148814000
C	13.207774000	7.365680000	3.205225000
H	13.685877000	7.331338000	2.221828000
H	13.975780000	7.060030000	3.920121000
H	11.151178000	2.993326000	4.467699000
C	12.755879000	8.783323000	3.530757000
H	12.311471000	8.791633000	4.531766000
H	13.639766000	9.422375000	3.594839000
C	11.760893000	9.381030000	2.549240000
H	10.907879000	8.706589000	2.433854000
H	12.223893000	9.450963000	1.557906000
C	11.235148000	10.746470000	2.969638000
H	10.665298000	10.634050000	3.897369000
H	10.517132000	11.094311000	2.223093000
C	12.308974000	11.797931000	3.166118000
H	12.960622000	11.562597000	4.008449000
H	12.942444000	11.893903000	2.281961000
H	11.875770000	12.778268000	3.363239000
C	12.796425000	5.695254000	6.437111000
H	13.329084000	5.257233000	7.284436000
H	13.571347000	5.825463000	5.675058000
C	12.292985000	7.063564000	6.809234000
C	13.231147000	8.056496000	7.100514000
C	10.945075000	7.409101000	6.845125000
C	12.840260000	9.349090000	7.400517000
C	10.548896000	8.707543000	7.141712000
C	11.492332000	9.683293000	7.415446000
H	14.286374000	7.806348000	7.074180000
H	10.183751000	6.666428000	6.638837000
H	13.589082000	10.101414000	7.614895000
H	9.494182000	8.952124000	7.156175000
H	11.182684000	10.695703000	7.641182000

TS$_{7,1EXP}$

Ru	10.698150000	3.928216000	2.444984000
O	11.851803000	4.447580000	4.211630000

P	9.622802000	2.236661000	3.734821000
P	10.138206000	3.640539000	0.238283000
O	8.332582000	5.734219000	2.723862000
N	12.380432000	2.373502000	2.117318000
C	10.780459000	0.822256000	3.822378000
H	10.379157000	0.065432000	4.500290000
H	10.789133000	0.399242000	2.814279000
C	12.171903000	1.195305000	4.206152000
C	12.701334000	0.796324000	5.425375000
H	12.055481000	0.293446000	6.138134000
C	14.044384000	0.981629000	5.714443000
H	14.451347000	0.666919000	6.666369000
C	14.867816000	1.518213000	4.736754000
H	15.934948000	1.602310000	4.915863000
C	14.363345000	1.951575000	3.518652000
C	12.979170000	1.879713000	3.268682000
C	15.285536000	2.456105000	2.446911000
H	15.693897000	3.442548000	2.709441000
H	16.170031000	1.813489000	2.392332000
C	14.619998000	2.514003000	1.102966000
C	13.210544000	2.491561000	1.014410000
C	15.386702000	2.585467000	-0.050514000
H	16.467388000	2.600178000	0.048121000
C	14.806854000	2.656719000	-1.307587000
H	15.420701000	2.724928000	-2.196326000
C	13.425234000	2.604551000	-1.406086000
H	12.951046000	2.601154000	-2.382079000
C	12.627165000	2.490233000	-0.274577000
C	11.159372000	2.264800000	-0.414050000
H	10.858320000	1.383997000	0.163323000
H	10.904942000	2.082658000	-1.458404000
C	9.555928000	2.774033000	5.512648000
H	10.623719000	2.791522000	5.757255000
C	9.018638000	4.177118000	5.742651000
H	9.596631000	4.928791000	5.207257000
H	9.079610000	4.419219000	6.805486000
H	7.971553000	4.277908000	5.454933000
C	8.871369000	1.767536000	6.424963000
H	7.787592000	1.778747000	6.297103000
H	9.069571000	2.019500000	7.467776000
H	9.215179000	0.744327000	6.272646000
C	8.001587000	1.416894000	3.382948000
H	7.779361000	0.821581000	4.273323000
C	6.889653000	2.439237000	3.209553000
H	6.755165000	3.053158000	4.099561000
H	5.939798000	1.939832000	3.011969000
H	7.080618000	3.119386000	2.378956000
C	8.120905000	0.456111000	2.204775000
H	8.682898000	0.880352000	1.371234000

H	7.132641000	0.188159000	1.827710000
H	8.622680000	-0.468069000	2.486255000
C	8.391331000	3.211692000	-0.245137000
H	8.050322000	2.640256000	0.619537000
C	7.484874000	4.429152000	-0.358678000
H	7.562351000	5.098618000	0.495217000
H	6.444795000	4.104736000	-0.422980000
H	7.692226000	5.007233000	-1.258584000
C	8.254912000	2.309557000	-1.463449000
H	8.635754000	2.773344000	-2.372870000
H	7.199095000	2.091347000	-1.634380000
H	8.762290000	1.354343000	-1.338007000
C	10.704620000	5.060372000	-0.816735000
H	11.787471000	4.995854000	-0.647566000
C	10.460111000	4.880174000	-2.307727000
H	10.799726000	3.914775000	-2.680715000
H	11.002945000	5.646233000	-2.863873000
H	9.406836000	4.989103000	-2.566408000
C	10.253179000	6.433257000	-0.344156000
H	9.187240000	6.597552000	-0.497092000
H	10.779113000	7.204924000	-0.908948000
H	10.470419000	6.603587000	0.710090000
C	9.267066000	5.050812000	2.635102000
C	12.197071000	5.391806000	3.433056000
S	11.340276000	7.034177000	3.645278000
C	13.649426000	5.483929000	3.026459000
H	14.212267000	5.720724000	3.936501000
H	13.943886000	4.475275000	2.751528000
C	12.503824000	7.755389000	4.841930000
H	13.475881000	7.897027000	4.361201000
H	12.109219000	8.757054000	5.023229000
C	12.648779000	6.989131000	6.141284000
H	12.923437000	5.951493000	5.932516000
H	11.678122000	6.936588000	6.642133000
H	11.734321000	5.104312000	1.844237000
C	13.991134000	6.456342000	1.911027000
H	13.372884000	6.225849000	1.038075000
H	13.723147000	7.472986000	2.208805000
C	15.440798000	6.386315000	1.533806000
C	15.832504000	5.917599000	0.284692000
C	16.432443000	6.755155000	2.443071000
C	17.175026000	5.812378000	-0.049377000
C	17.773750000	6.652030000	2.114416000
C	18.150250000	6.176483000	0.865241000
H	15.072447000	5.619560000	-0.429164000
H	16.145877000	7.128308000	3.421458000
H	17.456342000	5.436245000	-1.025625000
H	18.528929000	6.943153000	2.833846000
H	19.198481000	6.092924000	0.608034000

C	13.689276000	7.604656000	7.060848000
H	14.651455000	7.646977000	6.537050000
H	13.426843000	8.646607000	7.273449000
C	13.860078000	6.845981000	8.363810000
H	14.109260000	5.802343000	8.144191000
H	12.900982000	6.809772000	8.891398000
C	14.917727000	7.435239000	9.279789000
H	14.667257000	8.478304000	9.496010000
H	15.874234000	7.468895000	8.749364000
C	15.079059000	6.668916000	10.575350000
H	14.148339000	6.649282000	11.144652000
H	15.364599000	5.632042000	10.391077000
H	15.843153000	7.107756000	11.216560000

ENERGY DATA

Structure	$E^{M06\text{-}L}_{dioxane}$	$E^{\omega B97M\text{-}V}_{dioxane}$	Imaginary Frequency	G *T = 393.15K* *1M except H_2 1 atm*
Energy Unit	Hartree	Hartree	cm^{-1}	kcal
***mer* Ru-1**	-1999.810797880	-1999.28016800428	-	-1254230.27585482
***fac* Ru-1**	-1999.801449520	-1999.26490851728	-	-1254219.70578762
**Ru-2**	-2477.877288940	-2477.29208568457	-	-1554142.54583807
**Ru-3**	-2476.686334650	-2476.10297105878	-	-1553407.58054920
**Ru-4**	-2954.751616380	-2954.11677281301	-	-1853320.85898582
**Ru-5**	-2154.909836280	-2154.33224427516	-	-1351479.36432243
**Ru-6**	-2153.706515730	-2153.12961731405	-	-1350734.74710722
**Ru-7**	-2630.576701440	-2629.95979826629	-	-1649918.09778665
**Ru-9**	-2307.605967980	-2306.99610280496	-	-1447253.21004146
**Ru-10**	-2307.587193350	-2306.97379216063	-	-1447242.09143268
**Ru-11**	-2476.662431010	-2476.08392690273	-	-1553396.08574142
**Ru-12**	-2631.790253900	-2631.16141720427	-	-1650657.77711903
$\mathbf{TS_{2,3'}}$	-2477.859020340	-2477.26959168525	-874.0267	-1554130.49954822
$\mathbf{TS_{2,3''}}$	-2631.748003250	-2631.11244241051	-492.9003	-1650635.24340026
$\mathbf{TS_{5,6}}$	-2154.871955050	-2154.28792319834	-1158.3399	-1351453.84536572
$\mathbf{TS_{6,1'}}$	-2153.696470480	-2153.12689213441	-552.9576	-1350734.24247726
$\mathbf{TS_{3,2}}$	-3108.598431910	-3107.91661407403	-767.5004	-1949798.78155686
$\mathbf{TS_{3,1}}$	-2630.547867020	-2629.92464442510	-473.5311	-1649900.51194658
$\mathbf{TS_{3,7}}$	-2630.574171460	-2629.95142374035	-137.2286	-1649913.28509010
$\mathbf{TS_{7,1}}$	-2630.537425060	-2629.92311187594	-521.3326	-1649899.55088570
$\mathbf{TS_{6,9}}$	-2307.595660070	-2306.97602922064	-107.5160	-1447242.00550160
$\mathbf{TS_{9,10}}$	-2307.594057650	-2306.98343267240	-51.4226	-1447243.25201796

**$TS_{10,1}$**	-2307.578402130	-2306.97254082900	-549.6418	-1447243.66752719
**$TS_{6,1''}$**	-2153.696470480	-2153.12689213441	-552.9576	-1350734.24247726
**$TS_{1,9}$**	-2308.768741550	-2308.14608760681	-1032.4375	-1447965.68300821
**$TS_{6,3}$**	-2631.767979580	-2631.13579953637	-701.1032	-1650646.32717070
**$TS_{3,11}$**	-2476.644778980	-2476.08533066612	-266.6858	-1553372.70147006
**EtSH**	-478.041678694	-477.98466865965	-	-299913.06547421
**EtOH**	-155.078401777	-155.03230402020	-	-97253.58235227
**Acetaldehyde**	-153.874569585	-153.83309722599	-	-96516.03726822
**Thioester**	-630.740857761	-630.65298004647	-	-395696.20827687
**Hydrogen**	-1.17096075594	-1.16071352637	-	-732.41513652
**Ester**	-307.791394577	-307.71269173245	-	-193044.20714894
**Hemithioacetal**	-631.922542586	-631.83584544011	-	-396423.70731798
**Thioaldehyde**	-476.813458464	-476.76581691957	-	-299161.28663748
**Hexanethiol**	-635.328131878	-635.20750309832	-	-398508.58065564
**3-phenyl-1-propanol**	-425.500724530	-425.39031119483	-	-266846.71876348
**Thioester (exptl)**	-1058.452091960	-1058.23728185302	-	-663884.38182892
**$TS_{3,1EXP}$**	-3058.260809650	-3057.50891230777	-471.0095	-1918086.68379268
**$TS_{7,1EXP}$**	-3058.257126710	-3057.51356641902	-509.0142	-1918091.96811578

REFERENCES

(1) Zou, Y.-Q.; von Wolff, N.; Anaby, A.; Xie, Y.; Milstein, D. "Ethylene glycol as an efficient and reversible liquid-organic hydrogen carrier" *Nat. Catal.* **2019**, *2*, 415-422.

(2) Gaussian 16, Revision C.01, Frisch, M.J.; Trucks, G. W.; Schlegel, H. B.; Scuseria, G. E.; Robb, M. A.; Cheeseman, J. R.; Scalmani, G.; Barone, V.; Petersson, G. A.; Nakatsuji, H.; Li, X.; Caricato, M.; Marenich, A. V.; Bloino, J.; Janesko, B. G.; Gomperts, R.; Mennucci, B.; Hratchian, H. P.; Ortiz, J. V.; Izmaylov, A. F.; Sonnenberg, J. L.; Williams-Young, D.; Ding, F.; Lipparini, F.; Egidi, F.; Goings, J.; Peng, B.; Petrone, A.; Henderson, T.; Ranasinghe, D.; Zakrzewski, V. G.; Gao, J.; Rega, N.; Zheng, G.; Liang, W.; Hada, M.; Ehara, M.; Toyota, K.; Fukuda, R.; Hasegawa, J.; Ishida, M.; Nakajima, T.; Honda, Y.; Kitao, O.; Nakai, H.; Vreven, T.; Throssell, K.; Montgomery, Jr. J. A.; Peralta, J. E.; Ogliaro, F.; Bearpark, M. J.; Heyd, J. J.; Brothers, E. N.; Kudin, K.N.; Staroverov, V. N.; Keith, T. A.; Kobayashi, R.; Normand, J.; Raghavachari, K.; Rendell, A. P.; Burant, J. C.; Iyengar, S. S.; Tomasi, J.; Cossi, M.; Millam, J. M.; Klene, M.; Adamo, C.; Cammi, R..; Ochterski, J. W.; Martin, R. L.; Morokuma, K.; Farkas, O.; Foresman, J. B.; Fox, D. J. Gaussian, Inc., Wallingford CT, 2016.

(3) Zhao, Y.; Truhlar, D. G. "A new local density functional for main-group thermochemistry, transition metal bonding, thermochemical kinetics, and noncovalent interactions" *J. Chem. Phys.* **2006**, *125*, 194101.

(4) Weigend, F.; Ahlrichs, R. "Balanced basis sets of split valence, triple zeta valence and quadruple zeta valence quality for H to Rn: Design and assessment of accuracy" *PCCP* **2005**, *7*, 3297-3305.

(5) Weigend, F. "Accurate Coulomb-fitting basis sets for H to Rn" *PCCP* **2006**, *8*, 1057-1065.

(6) Grimme, S.; Antony, J.; Ehrlich, S.; Krieg, H. "A consistent and accurate ab initio parametrization of density functional dispersion correction (DFT-D) for the 94 elements H-Pu" *J. Chem. Phys.* **2010**, *132*, 154104.

(7) Neese, F. "Software update: the ORCA program system, version 4.0" *WIREs Computational Molecular Science* **2018**, *8*, e1327.

(8) Mardirossian, N.; Head-Gordon, M. "ωB97M-V: A combinatorially optimized, range-separated hybrid, meta-GGA density functional with VV10 nonlocal correlation" *J. Chem. Phys.* **2016**, *144*, 214110.

(9) (a) Vydrov, O. A.; Van Voorhis, T. "Nonlocal van der Waals density functional: The simpler the better" *J. Chem. Phys.* **2010**, *133*, 244103. (b) Hujo, W.; Grimme, S. "Performance of the van der Waals Density Functional VV10 and (hybrid)GGA Variants for Thermochemistry and Noncovalent Interactions" *J. Chem. Theory and Comp.* **2011**, *7*, 3866-3871.

(10) Hellweg, A.; Hättig, C.; Höfener, S.; Klopper, W. "Optimized accurate auxiliary basis sets for RI-MP2 and RI-CC2 calculations for the atoms Rb to Rn" *Theor. Chem. Acc.* **2007**, *117*, 587-597.

(11) Iron, M. A.; Janes, T. "Evaluating Transition Metal Barrier Heights with the Latest Density Functional Theory Exchange–Correlation Functionals: The MOBH35 Benchmark Database" *J. Phys. Chem. A* **2019**, *123*, 3761-3781.

(12) Marenich, A. V.; Cramer, C. J.; Truhlar, D. G. "Universal Solvation Model Based on Solute Electron Density and on a Continuum Model of the Solvent Defined by the Bulk Dielectric Constant and Atomic Surface Tensions" *J. Phys. Chem. B* **2009**, *113*, 6378-6396.

(13) Cramer, C.J. *Essentials of Computational Chemistry: Theories and Models. 2nd Edition*; John Wiley & Sons Ltd: West Sussex, England, 2004.

(14) For some discussion or examples of standard state corrections, see: (a) Sparta, M.; Riplinger, C.; Neese, F. "Mechanism of Olefin Asymmetric Hydrogenation Catalyzed by Iridium Phosphino-Oxazoline: A Pair Natural Orbital Coupled Cluster Study" *J. Chem. Theory and Computation* **2014**, *10*, 1099-1108. (b) Hopmann, K. H. "How Accurate is DFT for Iridium-Mediated Chemistry?" *Organometallics* **2016**, *35*, 3795-3807. (c) Gusev, D. G. "Revised Mechanisms of the Catalytic Alcohol Dehydrogenation and Ester Reduction with the Milstein PNN Complex of Ruthenium" *Organometallics* **2020**, *39*, 258-270.

(15) Munkerup, K.; Thulin, M.; Tan, D.; Lim, X.; Lee, R.; Huang, K.-W. "Importance of thorough conformational analysis in modeling transition metal-mediated reactions: Case studies on pincer complexes containing phosphine groups" *J. Saudi Chem. Soc.* **2019**, *23*,

1206-1218.

(16) Krogh-Jespersen, K.; Czerw, M.; Summa, N.; Renkema, K. B.; Achord, P. D.; Goldman, A. S. "On the Mechanism of (PCP)Ir-Catalyzed Acceptorless Dehydrogenation of Alkanes: A Combined Computational and Experimental Study" *J. Am. Chem. Soc.* **2002**, *124*, 11404-11416.
